# Effective Formation of New C(sp^2^)−S Bonds via Photoactivation of Alkylamine‐based Electron Donor‐Acceptor Complexes

**DOI:** 10.1002/chem.202203353

**Published:** 2022-12-05

**Authors:** Jorge C. Herrera‐Luna, María Carmen Pérez‐Aguilar, Leon Gerken, Olga García Mancheño, M. Consuelo Jiménez, Raúl Pérez‐Ruiz

**Affiliations:** ^1^ Departamento de Química Universitat Politècnica de València (UPV) Camí de Vera S/N 46022 Valencia Spain; ^2^ Organic Chemistry Institute University of Münster Corrensstrasse 36 48149 Münster Germany

**Keywords:** EDA complex, heteroarene thiolation, single-electron transfer, sustainability, visible-light

## Abstract

A novel visible light promoted formation of C_Aryl‐_S bonds through electron donor‐acceptor (EDA) complexes of alkylamines with 5‐ and 6‐membered (hetero)arene halides is presented. This represents the first EDA‐based thiolation method not relying on π‐π or a thiolate‐anion‐π interactions and provides a facile access to heteroarene radicals, which can be suitably trapped by disulfide derivatives to form the corresponding versatile arylsulfides. Mechanistic investigations on the aspects of the whole process were conducted by spectroscopic measurements, demonstrating the hypothesized EDA complex formation. Moreover, the strength of this method has been proven by a gram‐scale synthesis of thiolated products and the late‐stage derivatization of an anticoagulant drug.

## Graphical abstract

**An effective approach** for the direct thiolation of five‐membered heteroarenes involving visible‐light‐absorbing EDA complexes is reported. The reaction takes place from an EDA complex between an alkylamine and the heteroarene halide. Selective photolysis to the EDA complex has allowed to generate the heteroarene radical that is suitably trapped by disulfide derivatives, providing a potent synthetic versatility.

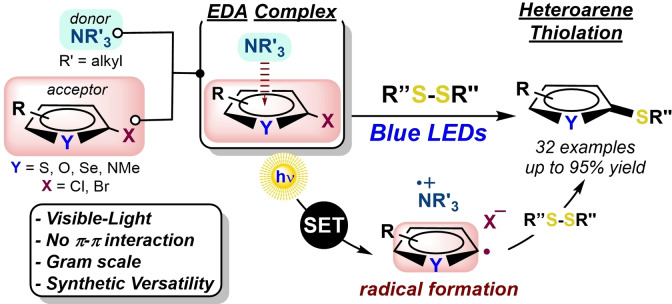

## Introduction

**Scheme 1:**
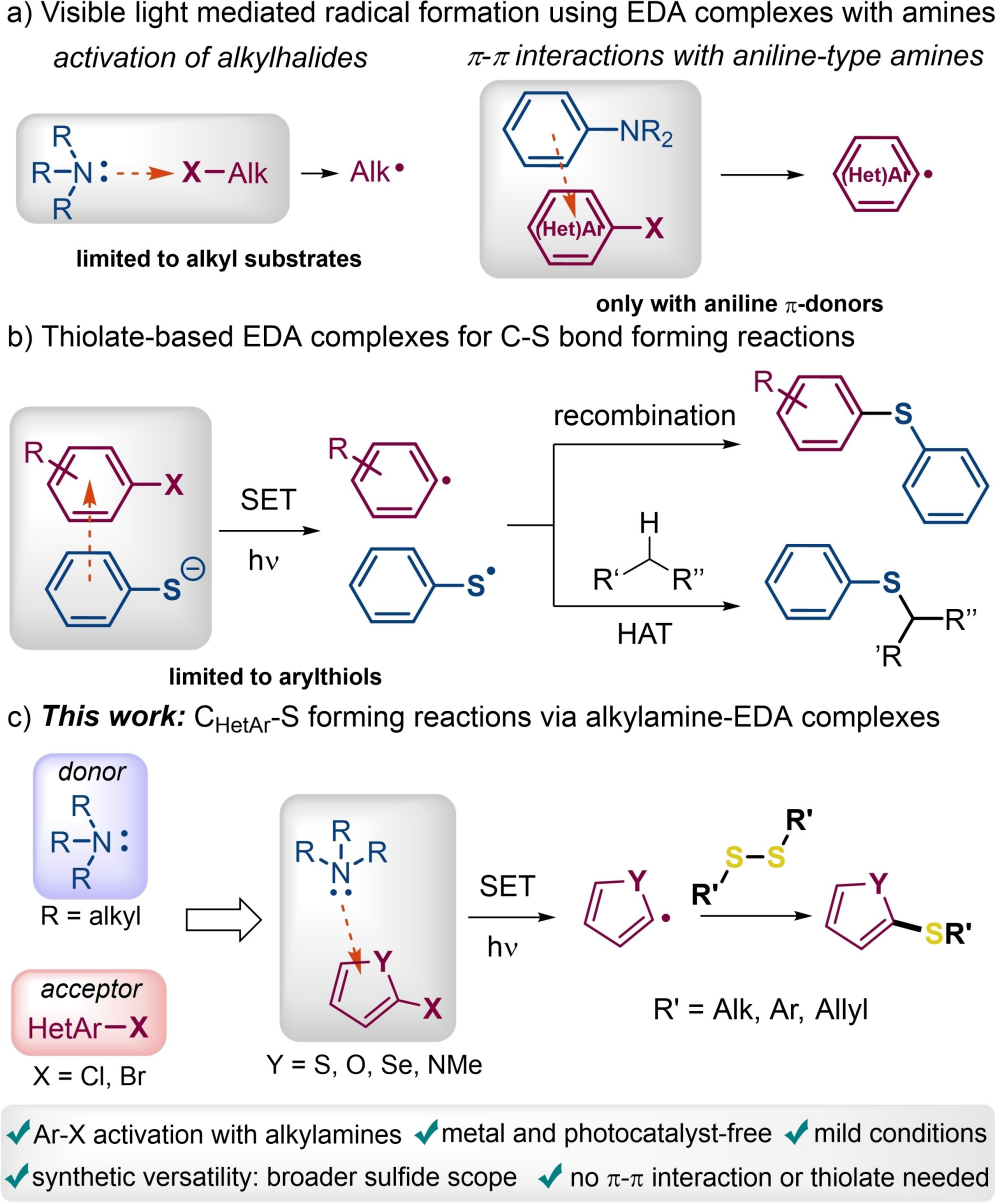
Amine‐based EDA complexes and C−S bond forming approaches.

The electron donor‐acceptor (EDA) complex photochemistry allows the mild and selective formation of radicals under visible light irradiation and has recently provided fresh prospects in synthetic chemistry, emerging as an active area of research.^[1]^ This approach relies on the association of electron‐rich (donors) and an electron‐poor (acceptors) compounds to give rise to a new molecular aggregation in the ground state, the so‐called EDA complex.^[2]^ Appearance of a new (and usually weak) absorption band at longer wavelengths (visible light regime), which is not present in the spectra of the individual partners, is a general feature of these species. Irradiation that triggers the intracomplex single‐electron‐transfer (SET) event, generating the desired radical intermediates under mild conditions. This approach has been hence employed for the controlled generation of C‐centered radicals by the activation of colorless organic compounds with visible light, opening new opportunities in organic synthesis. In particular, it has been well‐established that tertiary alkylamines such as *N,N‐*diisopropylethylamine (DIPEA, the Hünig's base)^[3]^ can be involved in EDA complexes with alkylhalides, generating the corresponding alkyl radicals in subsequent SET reactions under visible light (Scheme 1a, left).^[4]^ Moreover, electron rich aromatic amines such as anilines can also form EDA complexes by π‐π‐interactions with aromatic substrates (Scheme 1a, right).^[5]^ However, these methods are limited in the type of substrates or N‐based donors that can be involved. Therefore, many scientists have been recently devoted to the development of more general EDA‐based synthetic strategies, being new C‐heteroatom bond forming reactions of particular interest.

In this regard, organosulfur compounds occupy a pivotal role in nature^[6]^ and drug discovery,^[7]^ for which there is a continuous demand on new, efficient methods towards C−S forming‐bond reactions. Besides many potent, classical coupling approaches based on transition metal catalysts that often still suffer from catalyst deactivation, harsh conditions or multistep synthesis,^[8]^ photocatalysis have recently emerged as a powerful alternative strategy for the formation of C−S bonds induced by visible light. In this vein, the photocatalytic thiolene reaction between thiols and alkenes or alkynes mediated by visible light towards aliphatic thioethers represents one of the most prominent approaches.^[9]^ Regarding aromatic thioethers, the thiolation of rings such as benzene derivatives,^[10]^ indoles^[11]^ and benzimidazoles^[12]^ have also been successfully achieved using visible light photoredox catalysis.

In spite of the efforts directed to investigate the fabrication of new C−S bonds, novel methodologies that implies the EDA complex photochemistry using visible light in the absence of both photoredox catalysts and transition metals remains still scarce (Scheme 1b).^[13]^ Furthermore, the existing approaches rely on EDAs formed by π‐π interactions between the donor and acceptor or a thiolate‐anion‐π interaction, as well as the often incorporation of both the donor and acceptor units in the product, which limit the structural diversity of the methods. To overcome some of the current limitations, we envisioned the use of simple tertiary alkylamines as donors for the formation of EDA complexes with (hetero)arylhalides towards C(sp^2^)‐functionalization.

In the present work, a simple and effective approach for the direct thiolation of five‐membered heteroarenes involving visible‐light‐absorbing EDA complexes between heteroarene halides and an alkylamine is reported for the first time. After generation of the heteroarene radical upon light irradiation, this open shell intermediate could be now trapped by an external sulfur‐substrate, in this case a disulfide derivative, providing a remarkable synthetic versatility (Scheme 1c). Our strategy would evade not only π‐π interactions but also the need to select highly polarized reagents with donor and acceptor properties that ultimately end up in the product skeleton.

## Results and Discussion

### Searching for optimal conditions

To address the stated hypothesis, we focused on the photocoupling reaction between 2‐acetyl‐5‐chlorothiophene (**1 a**) and dimethyl disulfide (**2 a**) using DIPEA as sacrificial donor (Table 1). For initial optimizations, an aerated anhydrous acetonitrile (anhACN) solution of **1 a** (0.1 mmol), **2 a** (0.3 mmol) and DIPEA (0.3 mmol) was photolyzed with blue LEDs (λ∼457 nm) for 2.5 h observing no changes in the starting materials (entry 1). This result could indicate the deactivation of any excited intermediate in the process by dissolved molecular oxygen, which rapidly diffuses into the organic medium. Accordingly, a nearly complete conversion of **1 a** was observed in argon atmosphere affording the desired product **3 a** with very good yield (entry 2). A set of control experiments documented the essential role of the donor and light in this coupling reaction (entries 3–4). Utilization of more eco‐friendly standard analytical grade ACN led to similar result than that obtained in anhACN (entry 5 vs. entry 2), whereas other solvents did not improve the corresponding yields of **3 a** (entries 6–8). Changing the sacrificial donor (e. g. use of Et_3_N, DIPA or DABCO) did not provide better outputs (entries 9–11). On the basis of literature data,^[14]^ the model reaction was carried out in the presence of K_2_CO_3_ in order to enhance the selectivity. Complete conversion of **1 a** was then observed after 2.5 h of irradiation, leading to the desired product **3 a** in 89 % yield (entry 12); indeed, the amount for both donor (DIPEA) and base (K_2_CO_3_) were optimized to 0.6 equiv. and 1.5 equiv.; respectively (entry 13), obtaining an excellent yield and selectivity (93 % for both cases).

**Table 1:** Optimizing the reaction conditions.^[a]^

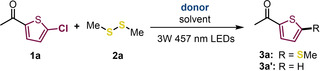					
Ent.	Solvent	Donor	Conv.^[b]^ %	**3 a**/**3 a’** ^[b]^	**3 a** Yield^[b]^ %
1^[c]^	anhACN	DIPEA	0	–	0
2	anhACN	DIPEA	98	76/24	74
3	anhACN	–	0	–	0
4^[d]^	anhACN	DIPEA	0	–	0
5	ACN	DIPEA	96	75/25	72
6	DMA	DIPEA	91	15/85	14
7	Acetone	DIPEA	97	54/46	52
8	MeOH	DIPEA	95	3/97	3
9	ACN	Et_3_N	21	81/19	17
10	ACN	DIPA	87	68/32	59
11	ACN	DABCO	0	–	0
12^[e]^	ACN	DIPEA+K_2_CO_3_	100	89/11	89
**13** ^[f]^	**ACN**	**DIPEA+K_2_CO_3_**	**100**	**93/7**	**93(70)** ^[g]^
14^[h]^	ACN	DIPEA+K_2_CO_3_	33	0/100	–
15^[i]^	CHCl_3_	DIPEA	0	–	–
16^[j]^	ACN	DIPEA+K_2_CO_3_	68	84/16	57
17^[k]^	ACN	DIPEA+K_2_CO_3_	91	75/25	68

[a] **1 a** (0.1 mmol), **2 a** (0.3 mmol), DIPEA (0.3 mmol) in 3 mL of N_2_/solvent; irradiation with 3 W blue (∼457 nm) LEDs at 23 °C for 2.5 h unless otherwise indicated. [b] Conv=**1 a** conversion; determined by GC‐FID using internal 1‐dodecanonitrile. Estimated error from randomly duplicated experiments independently ±3 % (further detail in the Supporting Information). [c] Under aerobic conditions. [d] Heating (50 °C) in dark. [e] DIPEA (0.1 mmol)+K_2_CO_3_ (0.3 mmol). [f] DIPEA (0.06 mmol)+K_2_CO_3_ (0.15 mmol). [g] Isolated yield in parentheses. [h] DIPEA (0.06 mmol)+K_2_CO_3_ (0.15 mmol) without **2 a**. [i] Without **2 a**.[j] 2‐Acetyl‐5‐bromothiophene (0.1 mmol), **2 a** (0.3 mmol), DIPEA (0.06 mmol)+K_2_CO_3_ (0.15 mmol). [k] 2‐Acetyl‐5‐iodothiophene (0.1 mmol), **2 a** (0.3 mmol), DIPEA (0.06 mmol)+K_2_CO_3_ (0.15 mmol).

To check whether the dechlorination of **1 a** could be mediated by the photochemistry of an EDA complex with DIPEA using blue LEDs at room temperature, we performed the model reaction in the absence of **2 a** (entry 14). Although prolongated irradiation time was required, the corresponding photoreduced compound **3 a’** was obtained as sole product within a 33 % conversion.

Furthermore, the interaction between **1 a** and DIPEA was also demonstrated by employing chloroform (CHCl_3_) as solvent, as the occurrence of CHCl_3_ may or may not deactivate the **1 a** dehalogenation process. The result clearly indicated that formation of **3 a’** was negligible (entry 15), and CHCl_3_ totally inhibited the process; in other words, the interaction between CHCl_3_ and DIPEA was predominant under these conditions.^[4b]^

Finally, the thioaryl bromide or iodide derivative were also submitted to this procedure, obtaining the coupling product **3 a** in good yields (entries 16–17). Therefore, the optimized conditions involved low loadings of all components related to **1 a** (3 equiv. of **2 a**, 1.5 equiv. of K_2_CO_3_ and catalytic amounts of DIPEA, 0.6 equiv.) and irradiation in the visible region at 457 nm with a blue LED in an anaerobic ACN solution for 2.5 h (see Supporting Information for further details).

### EDA complex formation in the ground state

In order to confirm the formation of the EDA complex between **1 a** and DIPEA in an unambiguous way, absorption spectroscopic studies of different mixtures in acetonitrile at room temperature were performed. Thus, the UV‐visible absorption spectra of **1 a** at a fixed concentration were recorded in the presence of increasing amounts of DIPEA, and then difference spectra (**1 a**+DIPEA)‐**1 a** were obtained. A new broad band was clearly observed from 345 nm to 500 nm, which was attributed to the EDA complex absorption (see Figure S1 in the Supporting Information). The equilibrium constant of EDA complex formation (K_EDA_) was estimated spectrophotometrically by the Benesi‐Hildebrand procedure [Equation (1), see typical plot in the inset of Figure S1 in the Supporting Information].^[15]^

(1) $$\left[1\;a\right]/\mathrm{Abs}{}_{\mathrm{EDA}}=\left[1/\left(K{}_{\mathrm{EDA}}\;\epsilon {}_{\mathrm{EDA}}\left[\mathrm{DIPEA}\right]\right)\right]+\left(1/\epsilon {}_{\mathrm{EDA}}\right)$$

**Figure 1:**
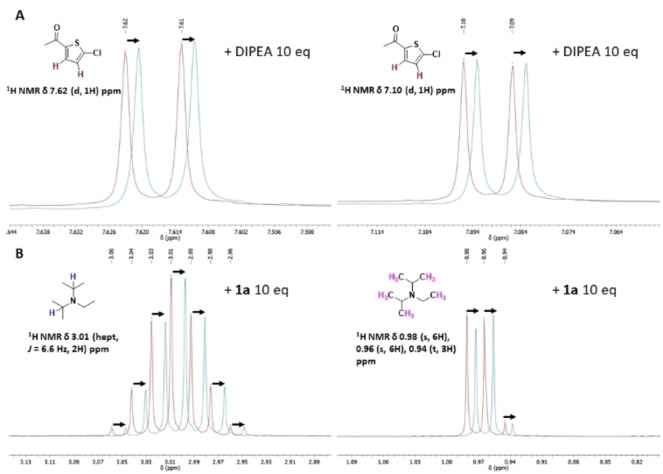
A: Relevant part of the ^1^H NMR spectra of the starting material **1 a** (0.033 M) in the absence (red) and in the presence of 10 equiv. of DIPEA (green). B: Relevant part of the ^1^H NMR spectrum of DIPEA (0.033 M) in the absence (red) and in the presence of 10 equiv. of 1a (green). Measurements were carried out in deuterated acetonitrile CD_3_CN.

Here, Abs_EDA_ means the absorbance due to the EDA band at 457 nm, at different concentrations of DIPEA, and ϵ_EDA_ represents the molar absorption coefficient. The ϵ_EDA_ value in acetonitrile was calculated from the intercept and found to be 0.2 M^−1^cm^−1^. The corresponding K_EDA_ value, as determined from the slope, was 9.53 M^−1^. Hence, this value was found to be compatible with previous similar systems,[^5^, ^16^] indicating a significant intermolecular interaction between **1 a** and DIPEA in the ground state. To further support this feature, NMR titration experiments showed that the ^1^H NMR resonance signals of the protons at the ring in the thiophene‐type halide **1 a** shifted to upfield upon addition of 10 equiv. of DIPEA (Figure 1A). Assuming that this interaction could also induce some effects on DIPEA, the most relevant part of the ^1^H NMR spectrum revealed again an upfield‐shifted variation of the signals corresponded to the protons at the centered CH and protons at the methyl of the isopropyl groups of DIPEA in the presence of 10 equiv. of **1 a** (Figure 1B).

### Single electron transfer (SET) upon direct excitation of the EDA complex

**Figure 2:**
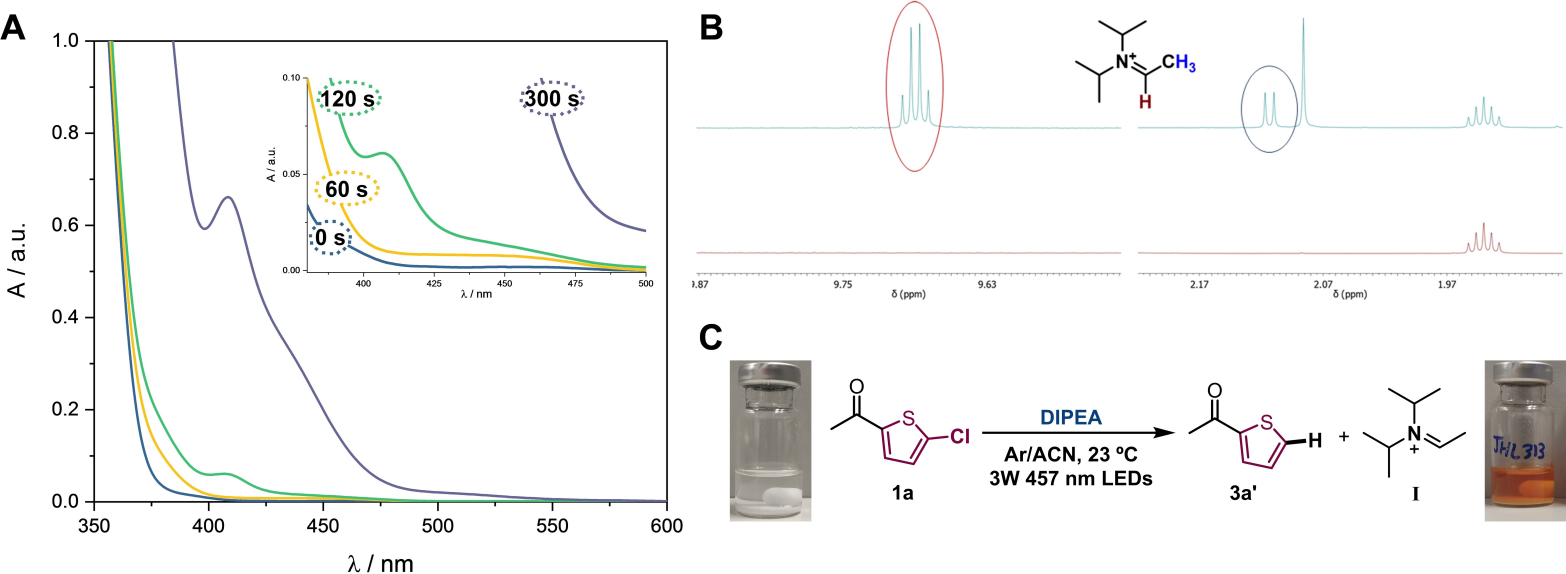
A: Time‐dependent UV‐Vis. spectra of dechlorination reaction of **1 a** (0.033 M) in the presence of DIPEA (0.1 M) recorded in Ar/ACN under blue light (λ ∼457 nm). B: Time‐dependent ^1^H NMR of 1a (0.016 mmol) in the presence of DIPEA (10 equiv.) before (bottom) and after (top) irradiation at 420 nm during 90 min in Ar/CD_3_CN. C: Photograph of the mixture solution containing **1 a**+DIPEA showing coloration caused by formation of the iminium ion (Table 1, entry 14).

As above mentioned, the involvement of DIPEA in other EDA complex systems and subsequent SET processes upon visible light irradiation was already reported.^[4]^ In these cases, color changes from colorless to deep yellow were observed in the corresponding dehalogenation reactions which was safely attributed to the formation of streptocyanine dyes^[17]^ through an iminium ion intermediate. Interestingly, formation of the iminium ion from DIPEA was directly detected by monitoring time‐resolved ^1^H NMR spectroscopy in CDCl_3_ where its characteristic peaks were observed at δ=9.79 ppm and δ=2.20 ppm. This fact proved unequivocally that a SET process had proceeded from the EDA complex irradiation. Inspired by these findings, the question arose whether our EDA complex system behaved at similar manner. As a matter of fact, new absorption band of a mixture of **1 a** and DIPEA during blue light irradiation (λ∼457 nm) started to evolve, and after 300 seconds the solution was deep yellow (Figure 2A). The long wavelength absorption band with maximum at 412 nm exactly matched as previously detected.^[4b]^ In addition, a ^1^H NMR experiment showed unequivocally the formation of the corresponding iminium ion due to appearance of the characteristic peaks (Figure 2B). Therefore, some conclusions could be drawn from these data: *i*) leading to the corresponding radical ion pairs a SET process occurred upon direct photolysis of the EDA complex, and *ii*) the unstable radical anion **1 a**⋅^−^ fragmented rapidly to afford the thiophene‐type radical that was capable to abstract a H‐atom from the DIPEA radical cation, giving rise the formal reduction product **3 a’** and the corresponding iminium ion (**I**). The latter was confirmed by the steady‐state irradiation (Table 1, entries 14 and 15) that indeed an orange‐colored solution was obtained (Figure 2C).

### Mechanism

**Scheme 2:**
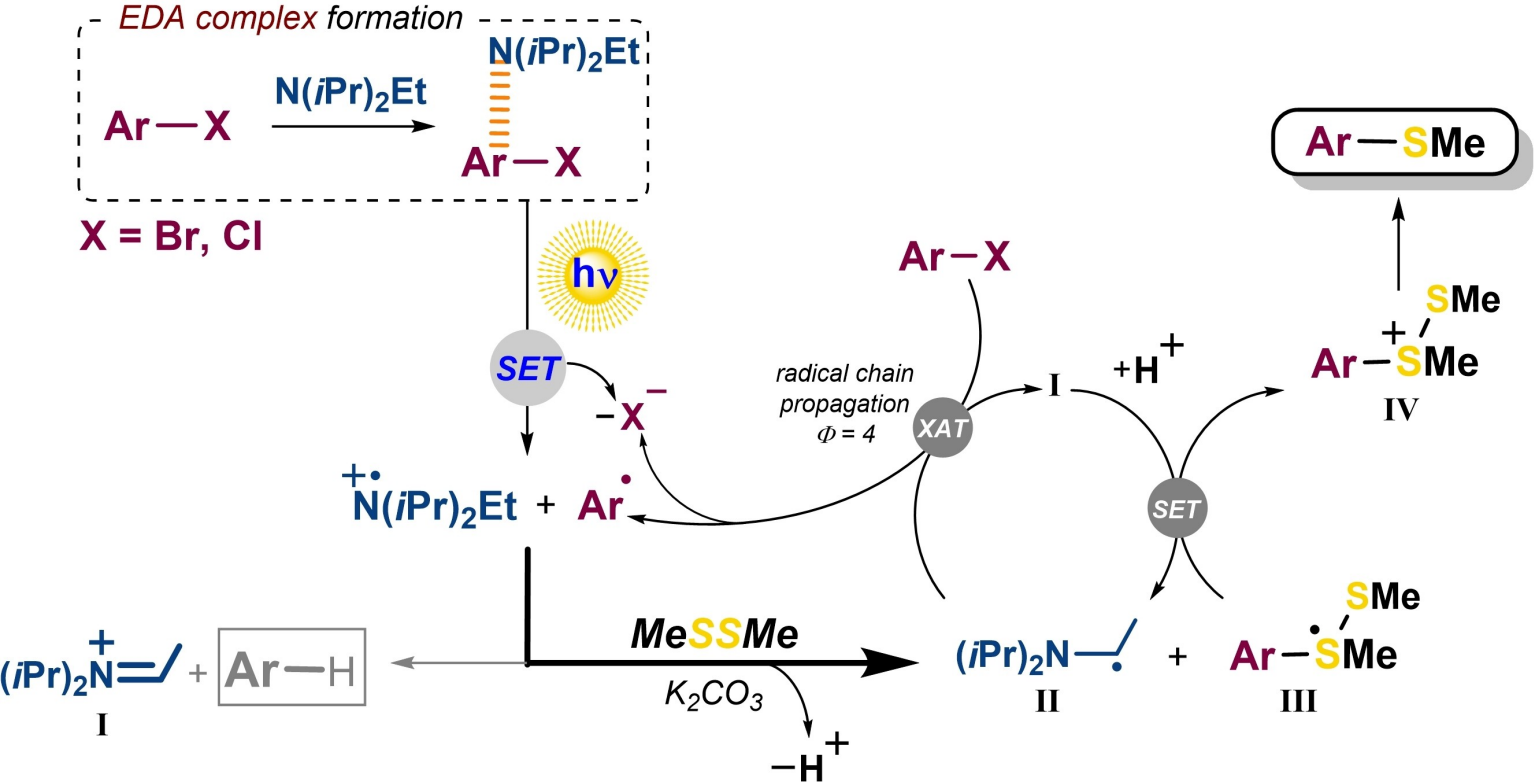
Visible‐light‐powered thiolation of heteroarenes through EDA complexes.

With these premises, we proposed a plausible general mechanism for the thiolation of **1 a** through EDA complex (Scheme 2). First, the interaction between the thiophene‐type halide **1 a** and DIPEA in the ground state led to the formation of an EDA complex which was capable to absorb at the visible region (400‐500 nm). Upon visible‐light irradiation, a SET process occurred from the donor moiety (DIPEA) to the acceptor (**1 a**), generating the DIPEA radical cation (DIPEA⋅^+^) and **1 a** radical anion (**1 a**⋅^−^). The latter then underwent fast irreversible fragmentation to give the anion Cl^−^ and the aryl radical Ar⋅ that could be properly trapped by a nucleophile agent such as **2 a** to produce the resultant trivalent sulfur‐based radical adduct **III**.

The selectivity of the process was controlled by the addition of K_2_CO_3_ as base that effectively deprotonated the DIPEA⋅^+^ to form the α‐aminoalkyl radical **II** and might quenched the possible HCl formed upon chloride fragmentation from the SET. This species might be now involved in the homolytic sp^2^ C−Cl bond cleavage of **1 a** via halogen‐atom transfer (XAT)^[18a]^ creating again Ar⋅ and the iminium ion **I**. A chain‐propagating process could be therefore taken place and, certainly, the quantum yield of the model reaction was found to be 4 (see details in the Supporting Information).^[18b]^

Based on previous data,[^10a^, ^10e^] systems close to adduct **III** were found to act as reducing agents to promote electron transfer to several photocatalyst radical cations such as eosinY⋅^+^ (*E°*[EY⋅^+^/EY]=+0.78 V vs. SCE) or *fac‐*Ir(ppy)_3_⋅^+^ ([*E°*[Ir^IV^/Ir^III^]=+0.76 V vs. SCE).

Given thus similar reduction potential of DIPEA⋅^+^ (*E°*[DIPEA⋅^+^/DIPEA]=+0.86 V vs. SCE),^[19]^ oxidation of adduct I**II** by the protonated **I** gave rise intermediate **IV** and DIPEA, allowing both the electronic balance of the whole process and employment of the amine in substoichiometric amounts (see optimal conditions); indeed, catalytic amounts of DIPEA (0.2 mol %) produced significant 72 % yield of **3 a** (see details in the Supporting Information). This fact was supported by the employment of DABCO as donor (Table 1, entry 11) and resulting in a negligible **3 a** formation since this amine contains two bridgehead nitrogen atoms, and therefore the acidity of its radical cation is markedly lower. Finally, the electrophilic species **IV** evolved to the desired thioether product by S−S bond cleavage.

### Scope

**Scheme 3:**
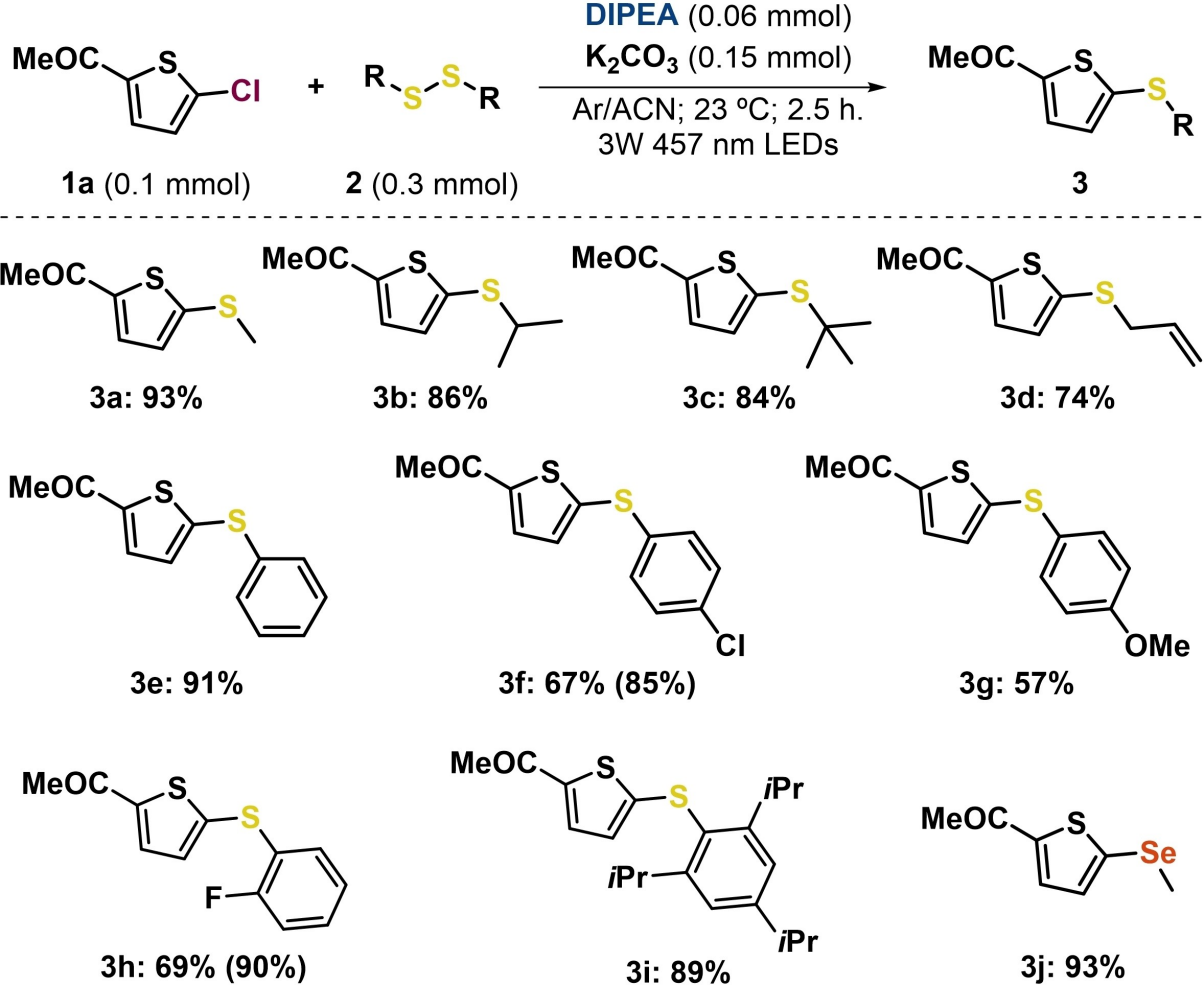
Reaction scope of **1 a** and disulfide derivatives through EDA complexes. Full conversion of compound **1 a** in all cases unless otherwise indicated in brackets. For detailed information on the reaction conditions, see the Supporting Information.

Having standardized the reaction conditions and understood the methodology, the substrate scope for the thiolation of heteroarene halides through an EDA complex was explored (Scheme 3). As the first step, the starting material **1 a** was then submitted to blue irradiation in the presence of a diverse set of commercially available disulfide derivatives bearing different alkyl chains such as isopropyl (*i*Pr), *tert‐*butyl or allyl under the standard reaction conditions. Gratifyingly, the corresponding coupling products (**3 b**–**3 d**) were obtained in high yields (74–86 %) together with full conversion of the starting material. Disulfide derivatives with aromatic rings also efficiently underwent the present reaction to afford the corresponding products **3 e**–**3 i** from good to excellent yields (57 %–91 %). Functional groups such as halogens (F, Cl), OMe or *i*Pr could be well tolerated under mild reaction conditions. In addition, the thiolation of **1 a** worked equally well with substrates possessing substituents at *ortho* and *para* positions (**3 f** and **3 h**). To further demonstrate the utility of the method, we performed our EDA complex strategy using the dimethyl diselenide as trapping agent, affording the desired product **3 j** in 93 % yield.

**Scheme 4:**
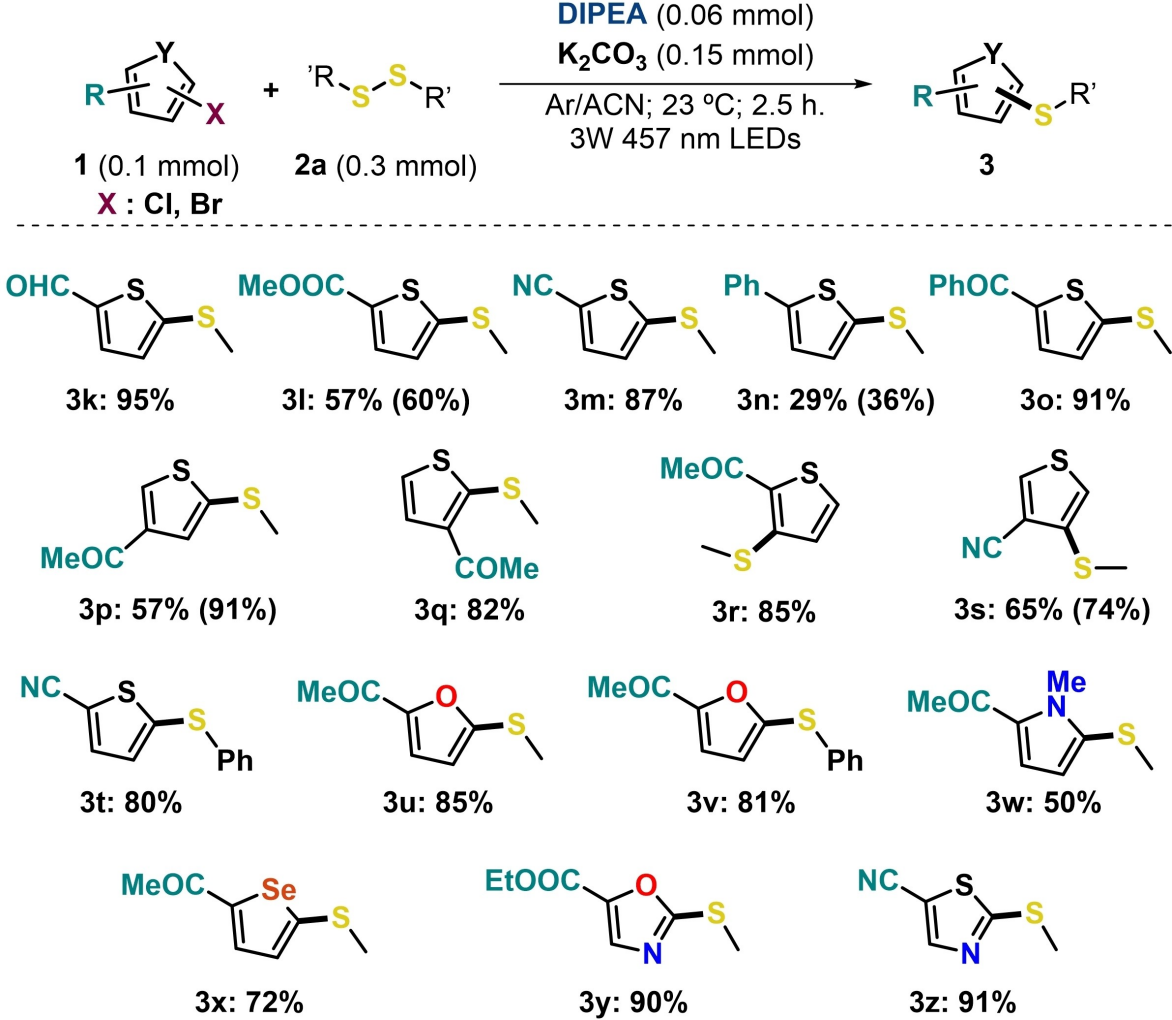
Reaction scope of 5‐membered heteroarene halides **1** through EDA complexes. Full conversion of starting materials **1** in all cases unless otherwise indicated in brackets. For detailed information on the reaction conditions, see the Supporting Information.

Encouraged by these results, we set out to study the substrate scope of this milder protocol to encompass 5‐membered thiophenes bearing different substitutions (Scheme 4). The derivatives presenting an aldehyde, ester, nitrile, phenyl or benzoyl group were capable to react with **2 a** under the previously optimized conditions, providing the corresponding products **3 k**–**3 o** in moderate to excellent yields (29 %‐95 %). Moreover, the reaction tolerated all possible positions of the halogen leaving group, as well as activating unit. Thus, the products **3 p**–**3 t** were successfully obtained in good to high yields from the thiolation of thiophene halides having the acetyl or the nitrile group shifted from position 2 to 3 or 4 in the heteroarene ring, or when the halogen atom is also in position 2 or 3. The feasibility of this novel procedure was also explored with other five‐membered haloheterocycles such as furan, pyrrole, selenophene, oxazole or thioxazole halides. The outcomes indicated that the coupling reaction of **2 a** or **2 e** with the corresponding heteroarenes brilliantly succeeded (**3 u**–**3 z**), with excellent yields in some cases (for instance, 90 % and 91 % for **3 y** and **3 z**, respectively).

**Scheme 5:**
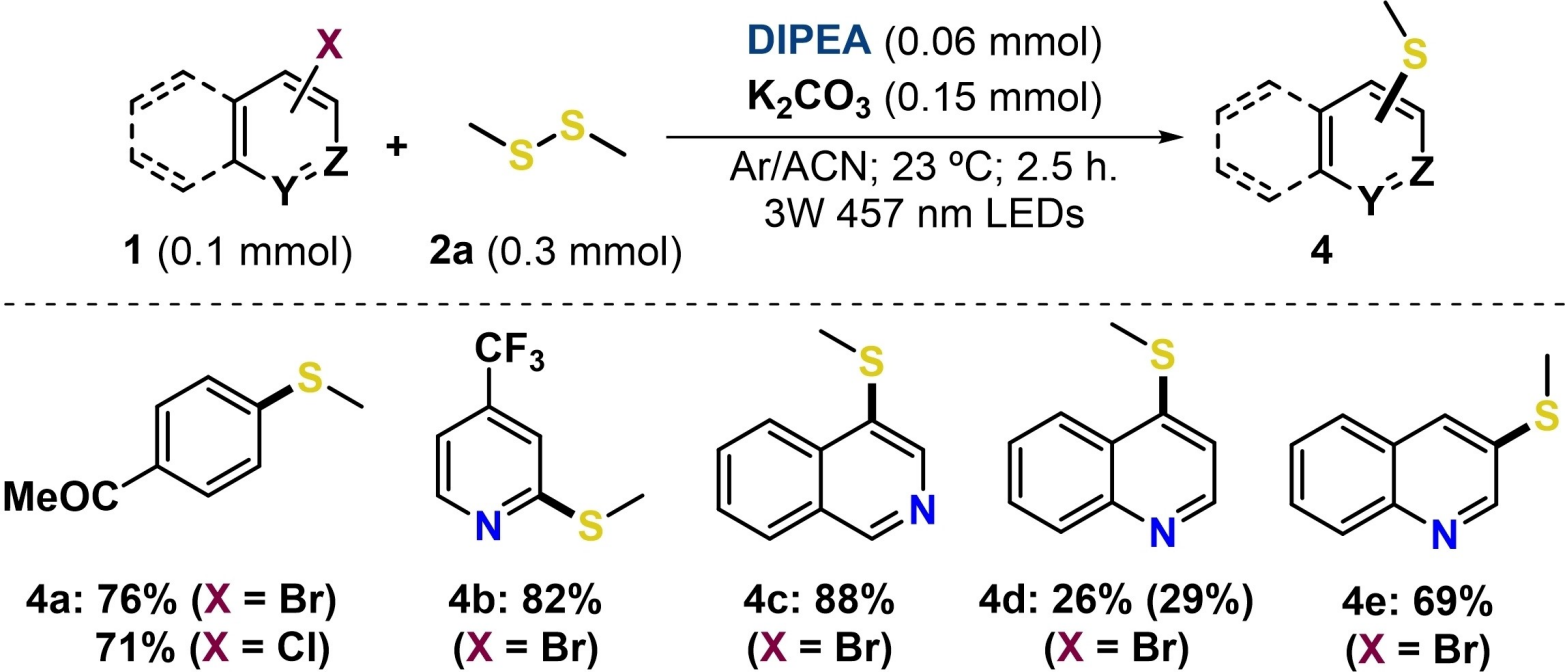
Reaction scope of 6‐membered (hetero)arene halides and dimethyl disulfide through EDA complexes. Full conversion of starting materials **1** in all cases unless otherwise indicated in brackets. For detailed information on the reaction conditions, see the Supporting Information.

To check the generality of our procedure, the study was expanded to the thiolation of 6‐membered ring (hetero)aryl halides (Scheme 5). To our delight, the simple 4‐bromo and 4‐chloro acetophenones were also reacted under the standard conditions using the DIPEA‐K_2_CO_3_ system, leading to the thioanisole derivative **4 a** in 76 % and 71 %, respectively. Moreover, other types of 6‐membered nitrogen‐containing haloarenes were also compatible under the optimized conditions. Hence, the reaction of 2‐bromo‐4‐trifluoromethyl pyridine, 4‐bromo isoquinoline and 4‐bromo quinoline provided the corresponding heteroaromatic methylsulfide products **4 b**–**4 d**, which were obtained in moderate to high yields (26 %–88 %).

**Scheme 6:**
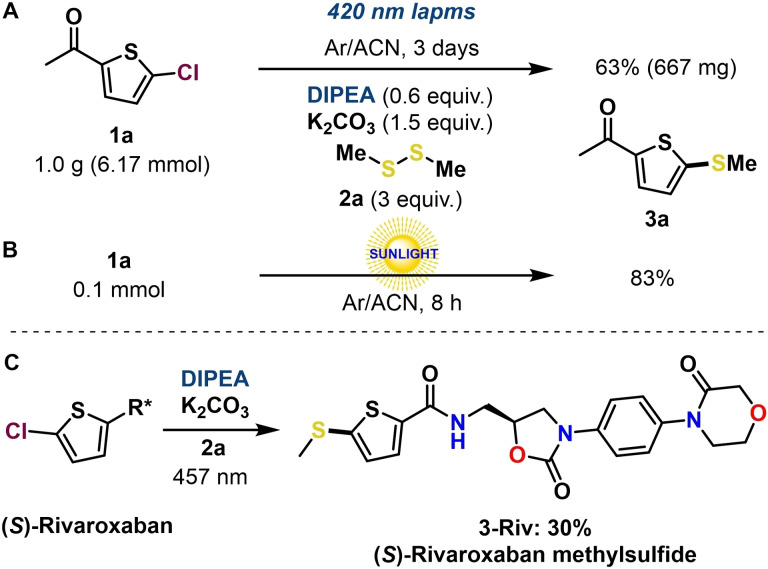
**A**: Upscaling of the model reaction. **B**: Use of sunlight irradiation. **C**: Application of a late stage thiolation. See the Supporting Information for detailed information.

In addition, the practicability and scalability of this protocol were successfully demonstrated by performing the model reaction at 1 gram scale under the standard conditions for 3 days, resulting in a **3 a** yield of 63 % (Scheme 6A; see Supporting Information for more details). Moreover, the reaction could also be performed under sunlight irradiation, which led to the desired product **3 a** in 83 % yield after 8 h of a whole sunny day (Scheme 6B; see Supporting Information for details).

Finally, the synthetic potential of the developed photochemical transformation through EDA complex was demonstrated by applying this method to the late stage thiolation of (*S*)‐rivaroxaban, an oral anticoagulant agent for the prevention and treatment of thromboembolic disorders.^[20]^ The corresponding thioether product **3‐Riv** was isolated in 30 % yield (Scheme 6C).

## Conclusion

In summary, a simple and effective metal‐free, visible light‐mediated thiolation of heteroarenes has been successfully achieved through an EDA complex approach with substoichiometric amounts of easily available trialkylamines. Selective photolysis to the EDA complex leads to the generation of the heteroarene radical that is suitably trapped by a disulfide derivative. This simple approach provides a potent, versatile, synthetic technique for the effective formation of new C(sp^2^)−S bonds that avoids the need of arylthiolates and/or π‐π interactions for the generation of the photoactive EDA complex. In particular, DIPEA shows the best performance as an electron donor in the presence of K_2_CO_3_ as base, allowing for the dehalogenative thiolation of a number of 5‐ and 6‐membered (hetero)arene halides up to 95 % yield. Mechanistic aspects of the whole process have been demonstrated by spectroscopic measurements, whereas the strength of this novel method has been proven by a gram‐scale experiment, the efficient use of sunlight irradiation, and the late‐stage derivatization of the anticoagulant drug (*S*)‐rivaroxaban.

## Experimental Section

**General procedure**: A vial (10 mL) with a stir bar was loading with the corresponding five‐membered heteroarene halide (100 μmol, 1.0 equiv.) and anhydrous K_2_CO_3_ (20.8 mg, 150 μmol, 1.5 equiv.). Then, DIPEA (10.5 μL, 60 μmol, 0.6 equiv.) and 1‐dodecanenitrile (22.1 μL, 100 μmol, 1.0 equiv.) were injected with a micro syringe. The vial was hermetically sealed with a cap septum. After that, the mixture was purged with argon bubbling for 8 minutes. The reaction was irradiated with an external blue LED (3 W 455–460 nm) through the plain bottom side of the vial at 22 °C until totally conversion of the starting material. Finally, brine (1 mL) was added, and the aqueous phase was extracted with ethyl acetate (3×2 mL). The reaction was examined by GC‐FID analysis. The organic phase was dried over anhydrous magnesium sulfate, filtered, and concentrated in vacuum. The crude was purified via TLC plastic sheet (20×20 cm) or flash column chromatography utilizing a hexane/ethyl acetate mixture as the mobile phase.

## Conflict of interest

The authors declare no conflict of interest.

1

## Supporting information

As a service to our authors and readers, this journal provides supporting information supplied by the authors. Such materials are peer reviewed and may be re‐organized for online delivery, but are not copy‐edited or typeset. Technical support issues arising from supporting information (other than missing files) should be addressed to the authors.

Supporting Information

## Data Availability

The data that support the findings of this study are available from the corresponding author upon reasonable request.
