# Supplementary material for: Effective Formation of New C(sp2)−S Bonds via Photoactivation of Alkylamine‐based Electron Donor‐Acceptor Complexes
Source: Chemistry. 2022 Dec 5;29(6):e202203353. doi: 10.1002/chem.202203353 (PMC10107790; doi:10.1002/chem.202203353)

Chemistry–A European Journal

Supporting Information

Effective Formation of New C(sp^2)–S Bonds via Photoactivation of Alkylamine-based Electron Donor-Acceptor Complexes

Jorge C. Herrera-Luna, María Carmen Pérez-Aguilar, Leon Gerken, Olga García Mancheño,* M. Consuelo Jiménez,* and Raúl Pérez-Ruiz*

TABLE OF CONTENTS

- Materials and methods S2
- Standard procedure S3
- Optimization S4
- EDA complex absorption spectrum S6
- Reaction quantum yield determination S7
- Sunlight irradiation and 1 gram scale S8
- Compound Characterization S9
- NMR spectra S19

Materials and methods

All reagents (≥97% purity) and solvents (≥99% purity) were purchased from commercial suppliers (Merck, TCI, Apollo Scientific, Fluorochem, Scharlab) and used as received unless otherwise indicated. Reactions were carried out in Metria®-Crimp Headspace clear vial flat bottom (10 mL, Ø 20 mm) sealed with Metria®-aluminium crimp cap with moulded septum butyl/natural PTFE (Ø 20 mm).

Irradiation was performed with a 3W blue LEDs (455-460 nm) from Avonec. TLC was performed on commercial SiO_2-coated aluminium and plastic sheets (DC60 F254, Merck). Visualization was done by UV-light (254nm).

Product were isolated materials after column flash chromatography or TLC on silica gel (Merck, mesh 35-70, 60 Å pore size) and their corresponding yields were determined by quantitative GC-FID measurements on an Agilent 8860 GC-System with N_2 as carrier gas. 1-Dodecanenitrile was employed as an internal standard in the GC-FID quantitative measurements; yield products were estimated as: [conversion × selectivity]/mass balance.

Determination of purity and structure confirmation of the literature known products was performed by ^{1}H NMR, ^{13}C NMR, and high-resolution mass spectrometry (HRMS) in case of unknown products. NMR spectral data were measured on a Bruker Advance 400 (400 MHz for ^{1}H, 101 MHz for ^{13}C) spectrometer at 20 °C. Chemical shifts are reported in δ/ppm, coupling constants *J* are given in Hertz. Solvent residual peaks were used as internal standard for all NMR measurements. The quantification of ^{1}H cores was obtained from integrations of appropriate resonance signals. Abbreviations used in NMR spectra: s – singlet, d – doublet, t – triplet, q – quartet, m – multiplet, dd – doublet of doublet, ddd – doublet of doublet of doublet, td – triplet of doublet and dq – doublet of quartet.

HRMS was carried out was performed in the mass facility of SCSIE University of Valencia. Absorption spectra were recorded on a JASCO V-630 spectrophotometer. The fluorescence spectra were recorded on an FS5 Edinburgh instrument spectrofluorometer with a SC-05 standard cuvette holder module.

Standard procedure

A vial (10 mL) with a stir bar was loading with the corresponding five-membered heteroarene halide (100 µmol, 1.0 equiv.) and anhydrous K_2CO_3 (20.8 mg, 150 µmol, 1.5 equiv.). Then, DIPEA (10.5 µL, 60 µmol, 0.6 equiv.) and 1-dodecanenitrile (22.1 µL, 100 µmol, 1.0 equiv.) were injected with a microsyringe. The vial was hermetically sealed with a cap septum. After that, the mixture was purged with argon bubbling for 8 minutes. The reaction was irradiated with an external blue LED (Avonec 3W 455-460 nm) through the plain bottom side of the vial at 22 °C until totally conversion of the starting material.

Finally, brine (1 mL) was added, and the aqueous phase was extracted with ethyl acetate (3 x 2 mL). The reaction was examined by GC-FID analysis. The organic phase was dried over anhydrous magnesium sulfate, filtered, and concentrated in vacuum. The crude was purified via TLC plastic sheet (20 x 20 cm) or flash column chromatography utilizing a hexane/ethyl acetate mixture as the mobile phase.

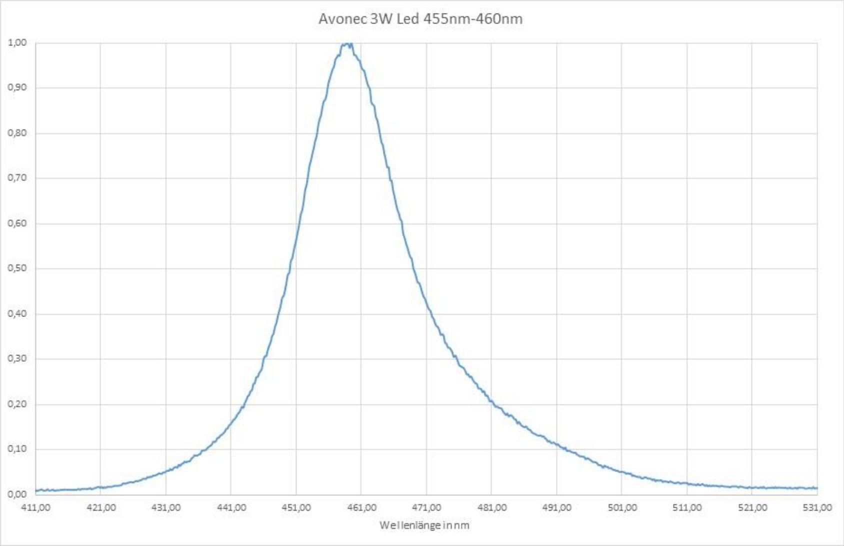

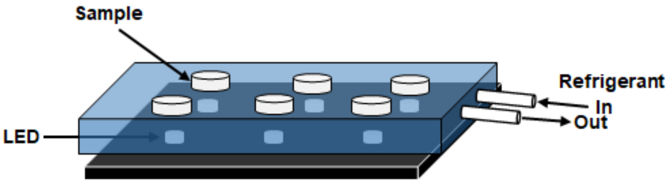

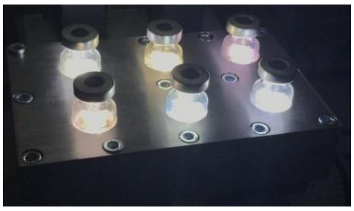

Irradiation setup

<u>Optimization</u>

Table S1. Searching of for the optimal conditions: Solvent and donor screening.[a]

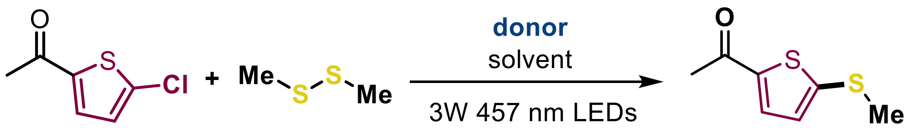

1a **2a** **3a**

Entry	Solvent / mL	Donor / Equiv.	*1a Conv / %*	*3a Yield / %*
1	Anh. ACN	-	-	-
2[b]	Anh. ACN	DIPEA (3)	-	-
3[c]	Anh. ACN	DIPEA (3)	-	-
4[d]	Anh. ACN	-	-	-
5	Anh. ACN	DIPEA (3)	98.1	74.6
6[e]	Anh. ACN	DIPEA (3)	-	-
7	**ACN**	**DIPEA** (3)	**96.2**	**72.6**
8	$CHCl_3$	DIPEA (3)	-	-
9	DMF	DIPEA (3)	63.6	12.2
10	DMA	DIPEA (3)	91.4	14
11	Acetone	DIPEA (3)	96.8	52
12	Toluene	DIPEA (3)	0	0
13	DMSO	DIPEA (3)	0	0
14	ACN/H_2O 9/1	DIPEA (3)	100	0
15	EtOAc	DIPEA (3)	0	0
16	MeOH	DIPEA (3)	95.2	2.4
17	ACN	Et_3N (3)	21.6	17.5
18	ACN	DIPA (3)	87	59
19	ACN	**DBU** (3)	100	78.6
20	ACN	DABCO (3)	1.5	1.3
21	ACN	K_2CO_3 (3)	0	0
22	ACN	Ph_3N (3)	47.8	26.4
23	ACN	Ph_3P (3)	8.9	8.9

[a]**1a** (16.2 mg, 0.1 mmol), solvent 4 mL; dimethyl disulfide (26.7 µL, 0.3 mmol, 3 equiv.) and irradiation with 3W blue LEDs (455-460 nm) at 22 °C for 2.5 hours unless otherwise indicated. [b]Reaction carried out without dimethyl disulfide and no irradiation source. [c] Reaction carried out without dimethyl disulfide and no irradiation source at 50 °C. [d]Reaction carried out without dimethyl disulfide, without DIPEA and no irradiation source. [e]Reaction carried out with 3W 515 nm LEDs.

Table S2. Donor-Base combination screening.[a]

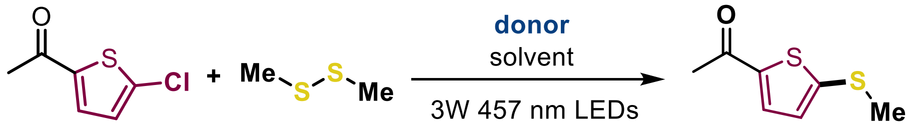

1a **2a** **3a**

Entry	Donor / Equiv.	Base / Equiv.	*1a Conv / %*	*3a Yield / %*
1	**DBU (1)**	**K_2CO_3 (3)**	**100**	**89**
2	**DIPEA (1)**	**K_2CO_3 (3)**	**100**	**89.2**
3	DIPEA (0.2)	K_2CO_3 (3)	76.7	72.3
4	DIPEA (0.6)	K_2CO_3 (3)	100	91.0
5	DIPEA (1.2)	K_2CO_3 (3)	100	90.4
6	DIPEA (0.6)	K_2CO_3 (2)	100	90.6
7	**DIPEA (0.6)**	**K_2CO_3 (1.5)**	**100**	**91.1**
8	DIPEA (0.6)	K_2CO_3 (1.2)	96.3	88.1
9	DIPEA (0.6)	K_2CO_3 (1.5)	85	79.4
10[b]	DIPEA (0.6)	K_2CO_3 (1.5)	72	68.6
11[c]	DIPEA (0.6)	K_2CO_3 (1.5)	81.4	76.4
12[d]	**DIPEA (0.6)**	**K_2CO_3 (1.5)**	**100**	**93.3**
13[e]	DIPEA (0.6)	K_2CO_3 (1.5)	100	91.1
14[f]	DIPEA (0.6)	K_2CO_3 (1.5)	100	90.6
15[g]	DIPEA (0.6)	K_2CO_3 (1.5)	100	88.5
16[h]	DIPEA (0.6)	K_2CO_3 (1.5)	*48.7*	*46.2*
17[i]	DIPEA (0.6)	K_2CO_3 (1.5)	56.5	53.1
18[j]	DIPEA (0.6)	K_2CO_3 (1.5)	87	81.5
19[k]	DIPEA (0.6)	K_2CO_3 (1.5)	90.2	85.1
20[l]	**DIPEA (0.6)**	**K_2CO_3 (1.5)**	**100**	**93**

[a]**1a** (16.2 mg, 0.1 mmol), solvent 4 mL; dimethyl disulfide (26.7 µL, 0.3 mmol, 3 equiv.) and irradiation with 3W blue LEDs (455-460 nm) at 22 °C for 2.5 hours unless otherwise indicated. [b]ACN 1 mL. [c]ACN 2 mL. [d]ACN 3 mL. [e]ACN 4 mL. [f]ACN 5 mL. [g]ACN 8 mL. [h]ACN 3 mL in 15 min of irradiation. [i]ACN 3 mL in 30 min of irradiation. [j]ACN 3 mL in 1 hour of irradiation. [k]ACN 3 mL in 1.5 hours of irradiation. [l]ACN 3 mL in 2 hours of irradiation.

EDA complex absorption spectrum

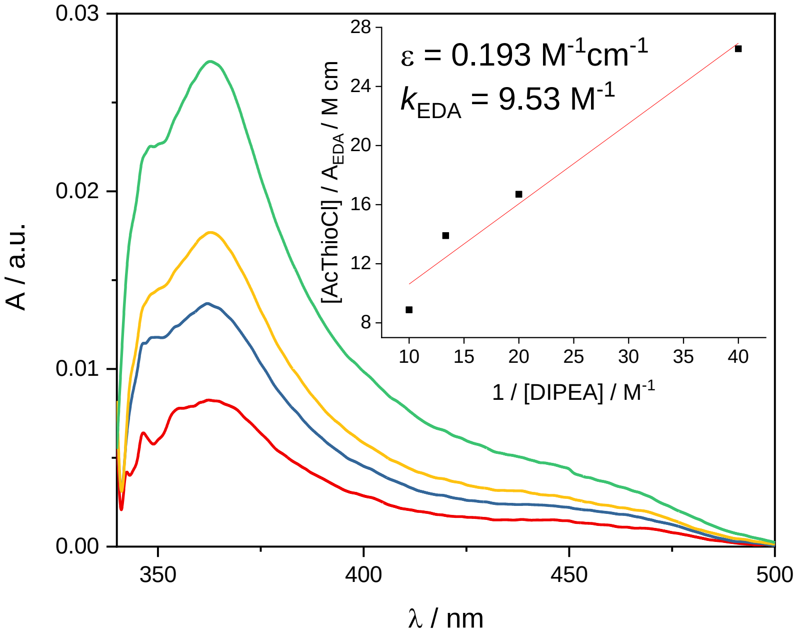

Figure S1. EDA complex formation recorded with the difference among the absorption spectrum of 2-acetyl-5-chlorothiophene 33 mM with increasing amounts of DIPEA, the absorption spectrum of 2-acetyl-5-chlorothiophene 33 mM, and the absorption spectra of the corresponding amount of DIPEA in ACN. a) spectrum of a mixture of 2-acetyl-5-chlorothiophene 33 mM + DIPEA 24.75 mM minus spectrum of 2-acetyl-5-chlorothiophene 33 mM, and minus spectrum of DIPEA 24.75 mM in ACN (red), b) spectrum of a mixture of 2-acetyl-5-chlorothiophene 33 mM + DIPEA 49.5 mM minus spectrum of 2-acetyl-5-chlorothiophene 33 mM, and minus spectrum of DIPEA 49.5 mM in ACN (blue), c) spectrum of a mixture of 2-acetyl-5-chlorothiophene 33 mM + DIPEA 74.25 mM minus spectrum of 2-acetyl-5-chlorothiophene 33 mM, and minus spectrum of DIPEA 74.25 mM in ACN (yellow), and d) spectrum of a mixture of 2-acetyl-5-chlorothiophene 33 mM + DIPEA 99 mM minus spectrum of 2-acetyl-5-chlorothiophene 33 mM, and minus spectrum of DIPEA 99 mM in ACN (green).

Reaction quantum yield ϕ determination

Determination of the photon flux at 457 nm via ferrioxalate actinometry

Following the procedure of Yoon[1] it is determined the photon flux of the LEDs (λ_{max}= 457 nm) by standard ferrioxalate actinometry. A 0.15 M solution of ferrioxalate was prepared with potassium ferrioxalate trihydrate (737 mg) and H_2SO_4 (10 mL of a 0.05 M solution). A solution of 1,10-phenanthroline (25 mg) was prepared with sodium acetate (5.63 g) in H_2SO_4 (25 mL of a 0.5 M). Both solutions were stored in the dark.

To determine the photon flux of the LED, the ferrioxalate solution (1 mL) is irradiated in a quartz cuvette for 90 seconds with 3W blue LEDs (λ_{max}= 457 nm). After irradiation, the phenanthroline solution (175 µL) was added to the ferrioxalate solution and the mixture was allowed to stir in the dark for 1 h with the aim of coordinate the ferrous ions to the phenanthroline. The absorbance of the solution was measured at 510 nm. It is measured the absorbance at 510 nm of a non-irradiated sample. The conversion was calculated according to the following formula:

$$mol\ Fe^{2+} = \frac{V \times \Delta A(510\ nm)}{l \times \varepsilon}$$

where *V* is the total volume (0.001175 L) of the solution with the phenanthroline addition, *ΔA* is the difference in absorbance at 510 nm between the irradiated and non-irradiated solutions, *l* is the path length (1.00 cm), and *ε* is the molar absorptivity of the ferrioxalate actinometer at 510 nm (11100 $L \cdot mol^{-1}\ cm^{-1}$).

The photon flux can be calculated with the following equation:

$$Photon\ flux = \frac{mol\ Fe^{2+}}{\Phi \times t \times f}$$

where *Φ* is the quantum yield for the ferrioxalate actinometer (0.85 at λ_{exc}= 457 nm),[2] *t* is the irradiation time (90 s), and *f* is the fraction of light absorbed at λ_{ex} = 457 nm by the ferrioxalate actinometer. This value is calculated using the following equation, where A(457 nm) is the absorbance of the ferrioxalate solution at 457 nm. An absorption spectrum gave an A(457 nm) value of about 2.02, indicating that the fraction of absorbed light (*f*) is around 0.99.

$$f = 1 - 10^{-A(457\ nm)}$$

The photon flux was calculated (average of three experiments) to be 3.19144×10^{-9} einsteins s^{-1}.

Determination of the reaction quantum yield

A vial (10 mL) with a stir bar was loading with 2-acetyl-5-chlorothiophene (16.2 mg, 100 µmol, 1.0 equiv.), anhydrous K_2CO_3 (20.8 mg, 150 µmol, 1.5 equiv.), DIPEA (10.5 µL, 60 µmol, 0.6 equiv.), 1-dodecanenitrile (22.1 µL, 100 µmol, 1.0 equiv.) and dimethyl disulfide (26.7 µL, 300 µmol, 3.0 equiv.). The vial was hermetically sealed with a cap septum and purged with argon bubbling for 8 minutes. The vial was irradiated with an external blue LED (Avonec 3W 455-460 nm) through the plain bottom side of the vial at 22 °C for 2.5 hours. The yield was determined by GC-FID with 1-dodecanenitrile as internal standard (0.093 mmol, 93%).

$$\Phi = \frac{mol\ of\ formed\ product}{photon\ flux \times t \times f}$$

with a photon flux of 3.19144×10^{-9} einsteins s^{-1}, t = 9000s and f > 0.999.

The reaction quantum yield (*Φ*) was determined as 4.09.

---

[1] Cismesia, M. A.; Yoon, T. P. *Chem. Sci.* **2015**, *6*, 5426

[2] Hamai, S.; Hirayama, F. *J. Phys. Chem.* **1983**, *87*, 83.

Sunlight irradiation and gram scale

Sunlight irradiation

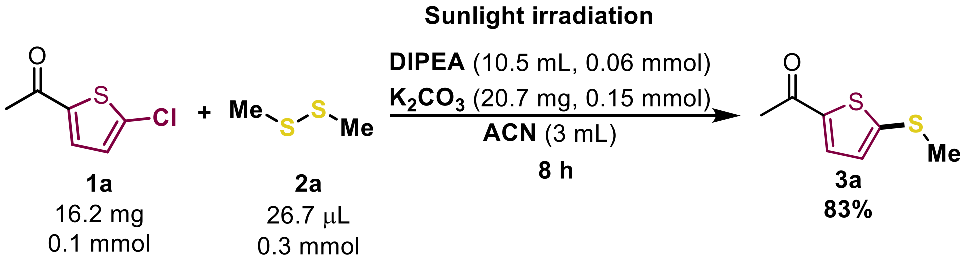

The compound **3a** was prepared according to the general procedure using 2-acetyl-5-chlorothiophene (16.2 mg, 100 µmol, 1.0 equiv.), dimethyl disulfide (26.7 µL, 300 µmol, 3 equiv.), dodecanenitrile (22.15 µL, 100 µmol, 1.0 equiv.) as internal standard and DIPEA (10.5 µL, 60 µmol, 0.6 equiv.) in ACN 3 mL. The mixture was irradiated for 8 hours, giving an 83% product yield according to GC-FID analysis (65% isolated yield as brown solid).

Location: Camí de Vera S/N, Chemistry Department, Universitat Politècnica de València (UPV), Valencia, Spain (coordinate: 39.482917, -0.341642), temperature: 27 °C, from 9:00 to 17:00. Date: 24/06/2022

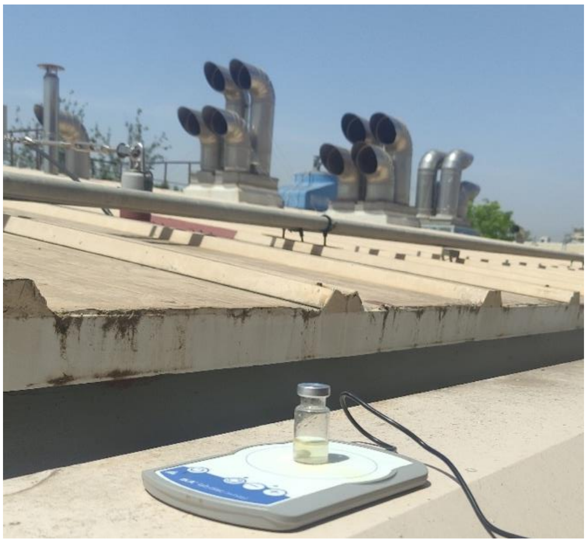

Gram scale

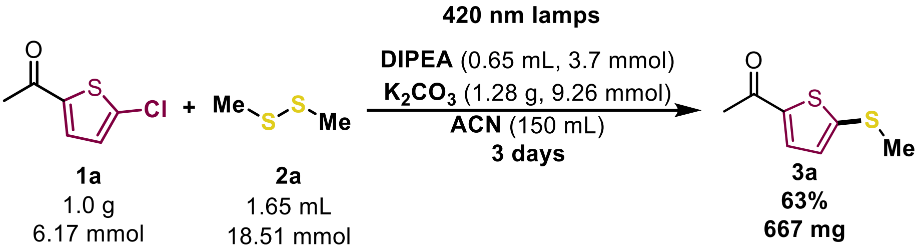

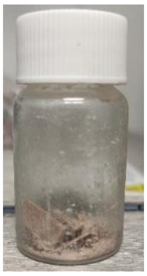

To scale-up the procedure from 16.2 mg (0.1 mmol to 6.17 mmol), we performed the reaction following the general procedure in a 250 mL flask under 420 nm lamps irradiation for 3 days using 2-acetyl-5-chlorothiophene (1.0 g, 6.17 mmol, 1.0 equiv.), dimethyl disulfide (1.65 mL, 18.51 mmol, 3 equiv.), DIPEA (0.65 mL, 3,7 mmol, 0.6 equiv.) and K_2CO_3 (1.28 g, 9.26 mmol, 1.5 equiv.) in ACN 150 mL. Then, brine (100 mL) was added, and the aqueous phase was extracted with ethyl acetate (3 x 100 mL). The organic phase was dried over anhydrous magnesium sulfate, filtered, and concentrated in vacuum. The crude was purified via flash column chromatography using a hexane/ethyl acetate mixture as the mobile phase obtaining 667 mg, 62.7% isolated yield.

COMPOUND CHARACTERIZATION

3a. 1-(5-(Methylthio)thiophen-2-yl)ethan-1-one

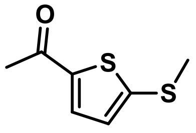

The compound (CAS: 22102-48-3) was prepared according to the general procedure using 2-acetyl-5-chlorothiophene (16.2 mg, 100 µmol, 1.0 equiv.), dimethyl disulfide (26.7 µL, 300 µmol, 3 equiv.), dodecanenitrile (22.15 µL, 100 µmol, 1.0 equiv.) as internal standard, K_2CO_3 (20.8 mg, 150 µmol, 1.5 equiv.) and DIPEA (10.5 µL, 60 µmol, 0.6 equiv.) in ACN 3 mL. The mixture was irradiated for 2 hours, giving a 93% product yield according to GC-FID analysis (77% isolated yield as brown powder).

^{1}H NMR (400 MHz, $CDCl_3$) δ 7.53 (d, *J* = 4.0 Hz, 1H), 6.92 (d, *J* = 4.0 Hz, 1H), 2.59 (s, 3H), 2.50 (s, 3H) ppm.
^{13}C NMR (100 MHz, $CDCl_3$) δ 189.6 (C, s), 150.5 (C, s), 143.5 (C, s), 133.2 (CH, s), 126.8 (CH, s), 26.4 (CH_3, s), 19.6 (CH_3, s) ppm.
HRMS (EI): m/z $(M+H)^+$ = calcd. for $C_7H_9OS_2$: 173.0089, found: 173.0089.

3b. 1-(5-(Isopropylthio)thiophen-2-yl)ethan-1-one

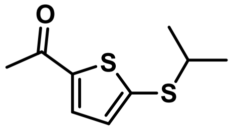

The compound (CAS: 98954-26-8) was prepared according to the general procedure using 2-acetyl-5-chlorothiophene (16.2 mg, 100 µmol, 1.0 equiv.), diisopropyl disulfide (49.8 µL, 300 µmol, 3 equiv.), dodecanenitrile (22.15 µL, 100 µmol, 1.0 equiv.) as internal standard, K_2CO_3 (20.8 mg, 150 µmol, 1.5 equiv.) and DIPEA (10.5 µL, 60 µmol, 0.6 equiv.) in ACN 3 mL. The mixture was irradiated for 2.5 hours, giving an 86% product yield according to GC-FID analysis (78% isolated yield as brown-yellow oil).

^{1}H NMR (400 MHz, $CDCl_3$) δ 7.55 (d, *J* = 3.9 Hz, 1H), 7.04 (d, *J* = 3.9 Hz, 1H), 3.34 (dt, *J* = 13.4, 6.7 Hz, 1H), 2.52 (s, 3H), 1.34 (s, 3H), 1.33 (s, 3H) ppm.
^{13}C NMR (100 MHz, $CDCl_3$) δ 190.0 (C, s), 146.0 (C; s), 145.6 (C, s), 132.8 (CH, s), 132.7 (CH, s), 41.9 (CH, s), 26.6 (CH_3, s), 23.2 (CH_3, s) ppm.
HRMS (EI): m/z $(M+H)^+$ = calcd. for $C_9H_{13}OS_2$: 201.0402, found: 201.0400.

3c. 1-(5-(*tert*-Butylthio)thiophen-2-yl)ethan-1-one

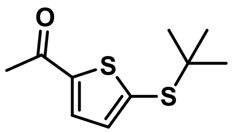

The compound (CAS: 2567707-98-4) was prepared according to the general procedure using 2-acetyl-5-chlorothiophene (16.2 mg, 100 µmol, 1.0 equiv.), dimethyl disulfide (26.7 µL, 300 µmol, 3 equiv.), dodecanenitrile (22.15 µL, 100 µmol, 1.0 equiv.) as internal standard, K_2CO_3 (20.8 mg, 150 µmol, 1.5 equiv.) and DIPEA (10.5 µL, 60 µmol, 0.6 equiv.) in ACN 3 mL. The mixture was irradiated for 4 hours, giving an 84% product yield according to GC-FID analysis (72% isolated yield as white powder).

^{1}H NMR (400 MHz, $CDCl_3$) δ 7.59 (d, *J* = 3.8 Hz, 1H), 7.13 (d, *J* = 3.8 Hz, 1H), 2.54 (s, 3H), 1.35 (s, 9H) ppm.
^{13}C NMR (100 MHz, $CDCl_3$) δ 190.2 (C, s), 148.0 (C, s), 142.4 (C, s), 137.3 (CH, s), 132.3 (CH, s), 48.3 (C, s), 30.86 (CH_3, s), 26.82 (CH_3, s) ppm.
HRMS (EI): m/z $(M+H)^+$ = calcd. for $C_{10}H_{15}OS_2$: 215.0559, found: 215.0552.

3d. 1-(5-(Allylthio)thiophen-2-yl)ethan-1-one

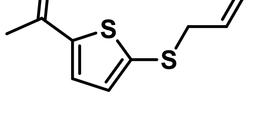

The compound was prepared according to the general procedure using 2-acetyl-5-chlorothiophene (16.2 mg, 100 µmol, 1.0 equiv.), diallyl disulfide (54.4 µL, 300 µmol, 3 equiv.), dodecanenitrile (22.15 µL, 100 µmol, 1.0 equiv.) as internal standard, K_2CO_3 (20.8 mg, 150 µmol, 1.5 equiv.) and DIPEA (10.5 µL, 60 µmol, 0.6 equiv.) in ACN 3 mL.

The mixture was irradiated for 2.5 hours, giving a 74% product yield according to GC-FID analysis (65% isolated yield as brown-yellow oil).

^{1}H NMR (400 MHz, $CDCl_3$) δ 7.55 (d, *J* = 3.8 Hz, 1H), 6.84 (dt, *J* = 3.8, 0.9 Hz, 1H), 5.97 (ddt, *J* = 16.7, 10.0, 6.7 Hz, 1H), 5.17 (ddq, *J* = 9.8, 6.7, 1.4 Hz, 2H), 3.59 (dd, *J* = 6.7, 1.1 Hz, 2H), 2.51 (s, 3H) ppm.
^{13}C NMR (100 MHz, $CDCl_3$) δ 190.7 (C, s), 153.0 (C, s), 142.9 (C, s), 135.2 (CH, s), 133.0 (CH, s), 126.2 (CH, s), 117.6 (CH, s), 34.9 (CH_2, s), 26.7 (CH_3, s) ppm.
HRMS (EI): m/z $(M+H)^+$ = calcd. for $C_9H_{11}OS_2$: 199.0246, found: 199.0244.

3e. 1-(5-(Phenylthio)thiophen-2-yl)ethan-1-one

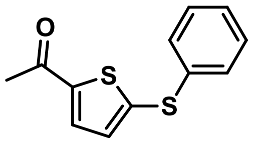

The compound (CAS: 90680-26-5) was prepared according to the general procedure using 2-acetyl-5-chlorothiophene (16.2 mg, 100 µmol, 1.0 equiv.), diphenyl disulfide (66.2 mg, 300 µmol, 3 equiv.), dodecanenitrile (22.15 µL, 100 µmol, 1.0 equiv.) as internal standard, K_2CO_3 (20.8 mg, 150 µmol, 1.5 equiv.) and DIPEA (10.5 µL, 60 µmol, 0.6 equiv.) in ACN 3 mL. The mixture was irradiated for 2.5 hours, giving a 91% product yield according to GC-FID analysis (79% isolated yield as yellow powder).

^{1}H NMR (400 MHz, $CDCl_3$) δ 7.56 (d, *J* = 3.9 Hz, 1H), 7.43 – 7.39 (m, 2H), 7.36 – 7.29 (m, 3H), 7.09 (d, *J* = 3.9 Hz, 1H), 2.50 (s, 3H) ppm.
^{13}C NMR (100 MHz, $CDCl_3$) δ 190.0 (C, s), 146.3 (C, s), 145.7 (C, s), 135.2 (C, s), 132.8 (CH, s), 132.1 (CH, s), 130.8 (CH, s), 129.6 (CH, s), 128.2 (CH, s), 26.6 (CH_3, s) ppm.
HRMS (EI): m/z $(M+H)^+$ = calcd. for $C_{12}H_{11}OS_2$: 235.0246, found: 235.0239.

3f. 1-(5-((4-Chlorophenyl)thio)thiophen-2-yl)ethan-1-one

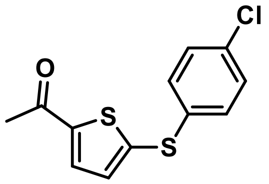

The compound was prepared according to the general procedure using 2-acetyl-5-chlorothiophene (16.2 mg, 100 µmol, 1.0 equiv.), 1,2-bis(4-chlorophenyl) disulfide (88.8 mg, 300 µmol, 3 equiv.), dodecanenitrile (22.15 µL, 100 µmol, 1.0 equiv.) as internal standard, K_2CO_3 (20.8 mg, 150 µmol, 1.5 equiv.) and DIPEA (10.5 µL, 60 µmol, 0.6 equiv.) in ACN 3 mL. The mixture was irradiated for 22 hours, giving a 67% product yield according to GC-FID analysis (59% isolated yield as pale-yellow powder).

^{1}H NMR (400 MHz, $CDCl_3$) δ 7.56 (d, *J* = 3.9 Hz, 1H), 7.34 – 7.27 (m, 4H), 7.11 (d, *J* = 3.9 Hz, 1H), 2.51 (s, 3H) ppm.
^{13}C NMR (100 MHz, $CDCl_3$) δ 190.0 (C, s), 146.8 (C, s), 144.5 (C, s), 134.4 (C, s), 133.9 (C, s), 132.8 (CH, s), 132.7 (CH, s), 131.9 (CH, s), 129.8 (CH, s), 26.7 (CH_3, s) ppm.
HRMS (EI): m/z $(M+H)^+$ = calcd. for $C_{12}H_{10}ClOS_2$: 268.9856, found: 268.9854.

3g. 1-(5-((4-Methoxyphenyl)thio)thiophen-2-yl)ethan-1-one

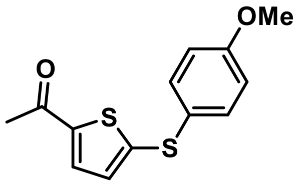

The compound (CAS: 1542495-11-3) was prepared according to the general procedure using 2-acetyl-5-chlorothiophene (16.2 mg, 100 µmol, 1.0 equiv.), 1,2-bis(4-methoxyphenyl) disulfide (86.1 mg, 300 µmol, 3 equiv.), dodecanenitrile (22.15 µL, 100 µmol, 1.0 equiv.) as internal standard, K_2CO_3 (20.8 mg, 150 µmol, 1.5 equiv.) and DIPEA (10.5 µL, 60 µmol, 0.6 equiv.) in ACN 3 mL. The mixture was irradiated for 15 hours, giving a 57% product yield according to GC-FID analysis (51% isolated yield as yellow powder).

^{1}H NMR (400 MHz, $CDCl_3$) δ 7.49 (d, *J* = 3.9 Hz, 1H), 7.48 – 7.44 (m, 2H), 6.93 (d, *J* = 3.9 Hz, 1H), 6.92 – 6.88 (m, 2H), 3.82 (s, 3H), 2.46 (s, 3H) ppm.

^{13}C **NMR** (100 MHz, $CDCl_3$) δ 189.8 (C, s), 160.7 (C, s), 150.3 (C, s), 144.5 (C, s), 135.1 (CH, s), 133.0 (CH, s), 128.4 (CH, s), 124.2 (C, s), 115.4 (CH, s), 55.6 (CH_3, s), 26.4 (CH_3, s) ppm.
HRMS (EI): m/z $(M+H)^+$ = calcd. for $C_{13}H_{13}O_2S_2$: 265.0351, found: 265.0351.

3h. 1-(5-((4-Methoxyphenyl)thio)thiophen-2-yl)ethan-1-one

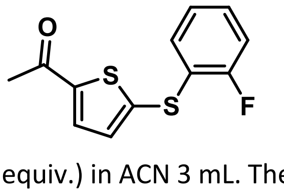

The compound was prepared according to the general procedure using 2-acetyl-5-chlorothiophene (16.2 mg, 100 µmol, 1.0 equiv.), 2,2'-difluorodiphenyldisulfide (56.4 µL, 300 µmol, 3.0 equiv.), dodecanenitrile (22.15 µL, 100 µmol, 1.0 equiv.) as internal standard, K_2CO_3 (41.5 mg, 300 µmol, 3.0 equiv.) and DIPEA (17.4 µL, 100 µmol, 1.0 equiv.) in ACN 3 mL. The mixture was irradiated for 24 hours, giving a 69% product yield according to GC-FID analysis (68% isolated yield as pale-yellow oil).

^{1}H **NMR** (400 MHz, $CDCl_3$) δ 7.55 (d, *J* = 4.0 Hz, 1H), 7.40 – 7.28 (m, 2H), 7.18 – 7.02 (m, 3H), 2.50 (s, 3H) ppm.
^{13}C **NMR** (100 MHz, $CDCl_3$) 190.0 (C, s), 162.3-159.8 (C, d, *J* = 248.1 Hz), 146.6 (C), 143.7 (C, d, *J* = 1.4 Hz), 133.3 (CH, s), 132.8 (CH, s), 132.6 (CH, s), 130.7 (CH, d, *J* = 7.9 Hz), 125.1 (CH, d, *J* = 3.9 Hz), 122.2 (C, d, *J* = 17.6 Hz), 116.4 (CH, d, *J*= 21.9 Hz), 26.6 (CH_3, s) ppm.
^{19}F **NMR** (377 MHz) δ -108.71 ppm.
HRMS (EI): m/z $(M+H)^+$ = calcd. for $C_{12}H_{10}FOS_2$: 253.0152, found: 253.0152.

3i. 1-(5-((2,4,6-Triisopropylphenyl)thio)thiophen-2-yl)ethan-1-one

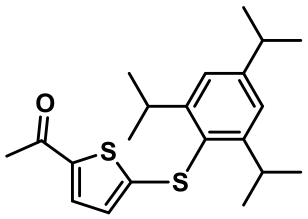

The compound was prepared according to the general procedure using 2-acetyl-5-chlorothiophene (16.2 mg, 100 µmol, 1.0 equiv.), bis(2,4,6-triisopropylphenyl) disulfide (141.3 mg, 300 µmol, 3.0 equiv.), dodecanenitrile (22.15 µL, 100 µmol, 1.0 equiv.) as internal standard, K_2CO_3 (41.5 mg, 300 µmol, 3.0 equiv.) and DIPEA (17.4 µL, 100 µmol, 1.0 equiv.). in ACN 3 mL. The mixture was irradiated for 24 hours, giving an 89% product yield according to GC-FID analysis (53% isolated yield as colorless oil).

^{1}H **NMR** (400 MHz, $CDCl_3$) δ 7.45 (d, *J* = 4.0 Hz, 1H), 7.10 (s, 2H), 6.71 (d, *J* = 4.0 Hz, 1H), 3.67 (hept, *J* = 6.8 Hz, 2H), 2.92 (hept, *J* = 6.9 Hz, 1H), 2.44 (s, 3H), 1.28 (d, *J* = 6.9 Hz, 6H), 1.19 (d, *J* = 6.9 Hz, 12H) ppm.
^{13}C **NMR** (100 MHz, $CDCl_3$) δ 189.6 (C, s), 153.9 (C, s), 153.2 (CH, s), 151.9 (C, s), 142.3 (C, s), 133.5 (CH, s), 125.4 (C, s), 124.5 (CH, s), 122.8 (CH, s), 34.5 (CH, s), 32.0 (CH, s), 26.2 (CH_3, s), 24.4 (CH_3, s), 24.0 (CH_3, s) ppm.
HRMS (EI): m/z $(M+Na)^+$ = calcd. for $C_{21}H_{28}OS_2Na$: 383.1471, found: 383.1474.

3j. 1-(5-(Methylselanyl)thiophen-2-yl)ethan-1-one

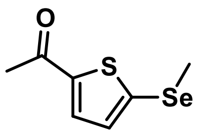

The compound (CAS: 29421-88-3) was prepared according to the general procedure using 2-acetyl-5-chlorothiophene (16.2 mg, 100 µmol, 1.0 equiv.), dimethyl diselenide (29.6 µL, 300 µmol, 3 equiv.), dodecanenitrile (22.15 µL, 100 µmol, 1.0 equiv.) as internal standard, K_2CO_3 (20.8 mg, 150 µmol, 1.5 equiv.) and DIPEA (10.5 µL, 60 µmol, 0.6 equiv.) in ACN 3 mL. The mixture was irradiated for 5 hours, giving a 93% product yield according to GC-FID analysis (82% isolated yield as yellow oil).

^{1}H **NMR** (400 MHz, $CDCl_3$) δ 7.51 (d, *J* = 3.9 Hz, 1H), 7.08 (d, *J* = 3.9 Hz, 1H), 2.51 (s, 3H), 2.46 (s, 3H) ppm.
^{13}C **NMR** (100 MHz, $CDCl_3$) δ 189.7 (C, s), 146.6 (C, s), 139.0 (C, s), 133.2 (CH, s), 131.4 (CH, s), 26.6 (CH_3, s), 10.8 (CH_3, s) ppm.
HRMS (EI): m/z $(M+H)^+$ = calcd. for C_7H_9OSSe: 220.9534, found: 220.9533.

3k. 5-(Methylthio)thiophene-2-carbaldehyde

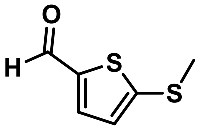

The compound (CAS: 24445-35-0) was prepared according to the general procedure using 5-bromo-2-thiophenecarbaldehyde (12.5 µL, 100 µmol, 1.0 equiv.), dimethyl disulfide (26.7 µL, 300 µmol, 3 equiv.), dodecanenitrile (22.15 µL, 100 µmol, 1.0 equiv.) as internal standard, K_2CO_3 (20.8 mg, 150 µmol, 1.5 equiv.) and DIPEA (10.5 µL, 60 µmol, 0.6 equiv.) in ACN 3 mL. The mixture was irradiated for 2.5 hours, giving a 95% product yield according to GC-FID analysis (82% isolated yield as brown-yellow powder).

^{1}H NMR (400 MHz, $CDCl_3$) δ 9.74 (s, 1H), 7.61 (d, *J* = 4.0 Hz, 1H), 6.98 (d, *J* = 4.0 Hz, 1H), 2.62 (s, 3H) ppm.
^{13}C NMR (100 MHz, $CDCl_3$) δ 181.5 (C, s), 153.1 (C, s), 142.4 (C, s), 137.3 (CH, s), 126.2 (CH, s), 19.2 (CH_3, s) ppm.
HRMS (EI): m/z $(M+H)^+$ = calcd. for $C_6H_7OS_2$: 158.9933, found: 158.9930.

3l. Methyl 5-(methylthio)thiophene-2-carboxylate

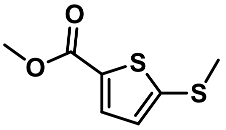

The compound (CAS: 773873-78-2) was prepared according to the general procedure using methyl 5-chlorothiophene-2-carboxylate (13.2 µL, 100 µmol, 1.0 equiv.), dimethyl disulfide (26.7 µL, 300 µmol, 3 equiv.), dodecanenitrile (22.15 µL, 100 µmol, 1.0 equiv.) as internal standard, K_2CO_3 (20.8 mg, 150 µmol, 1.5 equiv.) and DIPEA (10.5 µL, 60 µmol, 0.6 equiv.) in ACN 3 mL. The mixture was irradiated for 22 hours, giving a 57% product yield according to GC-FID analysis (49% isolated yield as yellow powder).

^{1}H NMR (400 MHz, $CDCl_3$) δ 7.64 (d, *J* = 3.9 Hz, 1H), 6.93 (d, *J* = 3.9 Hz, 1H), 3.86 (s, 3H), 2.57 (s, 3H) ppm.
^{13}C NMR (100 MHz, $CDCl_3$) δ 162.2 (C, s), 147.5 (C, s), 134.1 (CH, s), 132.7 (C, s), 127.9 (CH, s), 52.3 (CH_3, s), 20.2 (CH_3, s) ppm.
HRMS (EI): m/z $(M+H)^+$ = calcd. for $C_7H_9O_2S_2$: 189.0038, found: 189.0034.

3m. 5-(Methylthio)thiophene-2-carbonitrile

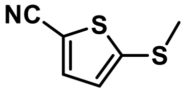

The compound (CAS: 175205-78-4) was prepared according to the general procedure using 5-bromo-2-thiophenecarbonitrile (11.1 µL, 100 µmol, 1.0 equiv.), dimethyl disulfide (26.7 µL, 300 µmol, 3 equiv.), dodecanenitrile (22.15 µL, 100 µmol, 1.0 equiv.) as internal standard, K_2CO_3 (20.8 mg, 150 µmol, 1.5 equiv.) and DIPEA (10.5 µL, 60 µmol, 0.6 equiv.) in ACN 3 mL. The mixture was irradiated for 2.5 hours, giving an 87% product yield according to GC-FID analysis (76% isolated yield as brown-yellow oil).

^{1}H NMR (400 MHz, $CDCl_3$) δ 7.47 (d, *J* = 3.9 Hz, 1H), 6.93 (d, *J* = 3.9 Hz, 1H), 2.59 (s, 3H) ppm.
^{13}C NMR (100 MHz, $CDCl_3$) δ 147.2 (C, s), 138.0 (CH, s), 127.5 (CH, s), 114.0 (C, s), 109.1 (C, s), 20.33 (CH_3, s) ppm.
HRMS (EI): m/z $(M+H)^+$ = calcd. for $C_6H_6NS_2$: 155.9936, found: 155.9929.

3n. 2-(Methylthio)-5-phenylthiophene

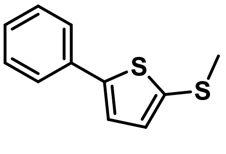

The compound (CAS: 478945-57-2) was prepared according to the general procedure 2-bromo-5-phenylthiophene (23.91 mg, 100 µmol, 1.0 equiv.), dimethyl disulfide (26.7 µL, 300 µmol, 3 equiv.), dodecanenitrile (22.15 µL, 100 µmol, 1.0 equiv.) as internal standard, K_2CO_3 (41.5 mg, 0.3 mmol, 3.0 equiv.) and DIPEA (17.4 µL, 0.10 mol, 1.0 equiv.) in ACN 3 mL. The mixture was irradiated for 24 hours, giving a 29% product yield according to GC-FID analysis (23% isolated yield as colorless oil).

^{1}H NMR (400 MHz, CD_2Cl_2) δ 7.58–7.54 (m, 2H), 7.41–7.35 (m, 2H), 7.29 (d, *J* = 7.4 Hz, 1H), 7.19 (d, *J* = 3.8 Hz, 1H), 7.05 (d, *J* = 3.8 Hz, 1H), 2.52 (s, 3H) ppm.
^{13}C NMR (100 MHz, CD_2Cl_2) δ 146.7 (C, s), 137.3 (C, s), 134.4 (C, s), 132.2 (CH, s), 129.4 (CH, s), 128.1 (CH, s), 125.9 (CH, s), 123.8 (CH, s), 22.3 (CH_3, s) ppm.
HRMS (EI): m/z $(M)^+$ = calcd. for $C_{11}H_{10}S_2$: 206.0218, found: 206.0218.

3o. (5-(Methylthio)thiophen-2-yl)(phenyl)methanone

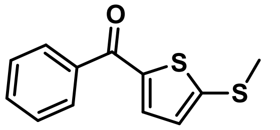

The compound (CAS: 22108-34-5) was prepared according to the general procedure using (5-bromothiophen-2-yl)(phenyl)methanone (27.3 mg, 100 µmol, 1.0 equiv.), dimethyl disulfide (26.7 µL, 300 µmol, 3 equiv.), dodecanenitrile (22.15 µL, 100 µmol, 1.0 equiv.) as internal standard, K_2CO_3 (20.8 mg, 150 µmol, 1.5 equiv.) and DIPEA (10.5 µL, 60 µmol, 0.6 equiv.) in ACN 3 mL. The mixture was irradiated for 2 hours, giving a 91% product yield according to GC-FID analysis (85% isolated yield as yellow powder).

^{1}H NMR (400 MHz, $CDCl_3$) δ 7.82 (dd, *J* = 8.3, 1.3 Hz, 2H), 7.57 (ddd, *J* = 6.8, 4.6, 1.3 Hz, 1H), 7.51 – 7.46 (m, 3H), 6.95 (d, *J* = 4.0 Hz, 1H), 2.63 (s, 3H) ppm.
^{13}C NMR (100 MHz, $CDCl_3$) δ 187.1 (C, s), 151.2 (C, s), 142.6 (C, s), 138.1 (C, s), 135.7 (CH, s), 132.2 (CH, s), 129.1 (CH, s), 128.6 (CH, s), 126.6 (CH, s), 19.5 (CH_3, s) ppm.
HRMS (EI): m/z $(M+H)^+$ = calcd. for $C_{12}H_{11}OS_2$: 235.0246, found: 235.0248.

3p. 1-(5-(Methylthio)thiophen-3-yl)ethan-1-one

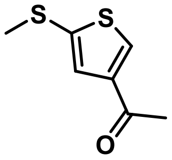

The compound was prepared according to the general procedure using 3-acetyl-5-chlorothiophene (16.2 mg, 100 µmol, 1.0 equiv.), dimethyl disulfide (26.7 µL, 300 µmol, 3 equiv.), dodecanenitrile (22.15 µL, 100 µmol, 1.0 equiv.) as internal standard, K_2CO_3 (20.8 mg, 150 µmol, 1.5 equiv.) and DIPEA (10.5 µL, 60 µmol, 0.6 equiv.) in ACN 3 mL. The mixture was irradiated for 24 hours, giving a 57% product yield according to GC-FID analysis (51% isolated yield as yellow powder).

^{1}H NMR (400 MHz, $CDCl_3$) δ 7.95 (d, *J* = 1.4 Hz, 1H), 7.45 (d, *J* = 1.4 Hz, 1H), 2.51 (s, 3H), 2.49 (s, 3H) ppm.
^{13}C NMR (100 MHz, $CDCl_3$) δ 191.9 (C, s), 142.9 (C, s), 139.6 (C, s), 134.0 (CH, s), 129.7 (CH, s), 27.3 (CH_3, s), 21.7 (CH_3, s) ppm.
HRMS (EI): m/z $(M+H)^+$ = calcd. for $C_7H_9OS_2$: 173.0089, found: 173.0084.

3q. 1-(2-(Methylthio)thiophen-3-yl)ethan-1-one

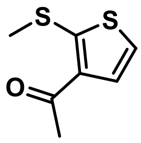

The compound (CAS: 42108-16-7) was prepared according to the general procedure using 3-acetyl-2-bromothiophene (20.5 mg, 100 µmol, 1.0 equiv.), dimethyl disulfide (26.7 µL, 300 µmol, 3 equiv.), dodecanenitrile (22.15 µL, 100 µmol, 1.0 equiv.) as internal standard, K_2CO_3 (20.8 mg, 150 µmol, 1.5 equiv.) and DIPEA (10.5 µL, 60 µmol, 0.6 equiv.) in ACN 3 mL. The mixture was irradiated for 17 hours, giving an 82% product yield according to GC-FID analysis (75% isolated yield as pale-yellow powder).

^{1}H NMR (400 MHz, $CDCl_3$) δ 7.38 (d, *J* = 5.5 Hz, 1H), 7.10 (d, *J* = 5.5 Hz, 1H), 2.58 (s, 3H), 2.50 (s, 3H) ppm.
^{13}C NMR (100 MHz, $CDCl_3$) δ 192.6 (C, s), 153.4 (C, s), 134.21 (C, s), 129.3 (CH, s), 121.6 (CH, s), 28.7 (CH_3, s), 18.7 (CH_3, s) ppm.
HRMS (EI): m/z $(M+H)^+$ = calcd. for $C_7H_9OS_2$: 173.0089, found: 173.0088.

3r. 1-(3-(Methylthio)thiophen-2-yl)ethan-1-one

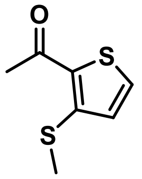

The compound (CAS: 74598-24-6) was prepared according to the general procedure using 2-acetyl-3-bromothiophene (21.1 mg, 100 µmol, 1.0 equiv.), dimethyl disulfide (26.7 µL, 300 µmol, 3 equiv.), dodecanenitrile (22.15 µL, 100 µmol, 1.0 equiv.) as internal standard, K_2CO_3 (20.8 mg, 150 µmol, 1.5 equiv.) and DIPEA (10.5 µL, 60 µmol, 0.6 equiv.) in ACN 3 mL. The mixture was irradiated for 6 hours, giving an 85% product yield according to GC-FID analysis (77% isolated yield as white powder).

^{1}H NMR (400 MHz, $CDCl_3$) δ 7.53 (d, *J* = 5.2 Hz, 1H), 7.03 (d, *J* = 5.2 Hz, 1H), 2.54 (s, 3H), 2.52 (s, 3H) ppm.
^{13}C NMR (100 MHz, $CDCl_3$) δ 189.9 (C, s), 145.6 (C, s), 131.2 (CH, s), 126.4 (CH, s), 28.9 (CH_3, s), 16.73 (CH_3, s) ppm.
HRMS (EI): m/z $(M+H)^+$ = calcd. for $C_7H_9OS_2$: 173.0089, found: 173.0089.

3s. 4-(Methylthio)thiophene-3-carbonitrile

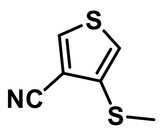

The compound was prepared according to the general procedure using 4-bromo-3-thiophenecarbonitrile (18.8 mg, 100 µmol, 1.0 equiv.), dimethyl disulfide (26.7 µL, 300 µmol, 3 equiv.), dodecanenitrile (22.15 µL, 100 µmol, 1.0 equiv.) as internal standard, K_2CO_3 (20.8 mg, 150 µmol, 1.5 equiv.) and DIPEA (10.5 µL, 60 µmol, 0.6 equiv.) in ACN 3 mL. The mixture was irradiated for 24 hours, giving an 65% product yield according to GC-FID analysis (61% isolated yield as pale-yellow powder).

^{1}H NMR (400 MHz, $CDCl_3$) δ 7.96 (d, *J* = 3.2 Hz, 1H), 7.09 (d, *J* = 3.2 Hz, 1H), 2.53 (s, 3H) ppm.
^{13}C NMR (100 MHz, $CDCl_3$) δ 137.0 (C, s), 136.7 (CH, s), 122.4 (CH, s), 114.1 (C, s), 113.2 (C, s), 17.9 (CH_3, s) ppm.
HRMS (EI): m/z $(M+Na)^+$ = calcd. for $C_6H_5NS_2Na$: 177.9761, found: 177.9764.

3t. 5-(Phenylthio)thiophene-2-carbonitrile

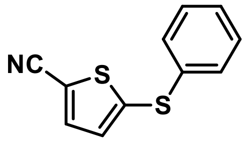

The compound (CAS: 1300106-13-1) was prepared according to the general procedure using 5-bromo-2-thiophenecarbonitrile (11.1 µL, 100 µmol, 1.0 equiv.), diphenyl disulfide (66.2 mg, 300 µmol, 3 equiv.), dodecanenitrile (22.15 µL, 100 µmol, 1.0 equiv.) as internal standard, K_2CO_3 (20.8 mg, 150 µmol, 1.5 equiv.) and DIPEA (10.5 µL, 60 µmol, 0.6 equiv.) in ACN 3 mL. The mixture was irradiated for 4 hours, giving an 80% product yield according to GC-FID analysis (73% isolated yield as colorless oil).

^{1}H NMR (400 MHz, $CDCl_3$) δ 7.50 (d, *J* = 3.9 Hz, 1H), 7.41 – 7.31 (m, 5H), 7.10 (d, *J* = 3.9 Hz, 1H) ppm.
^{13}C NMR (100 MHz, $CDCl_3$) δ 144.4 (C, s), 137.9 (CH, s), 134.8 (C, s), 131.9 (CH, s), 130.8 (CH, s), 129.8 (CH, s), 128.5 (CH, s), 113.7 (C, s), 112.1 (C, s) ppm.
HRMS (EI): m/z $(M+H)^+$ = calcd. for $C_{11}H_8NS_2$: 218.0093, found: 218.0089.

3u. 1-(5-(Methylthio)furan-2-yl)ethan-1-one

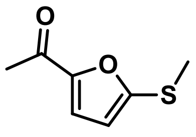

The compound (CAS: 934-64-5) was prepared according to the general procedure using 2-acetyl-5-bromofuran (19.9 mg, 100 µmol, 1.0 equiv.), dimethyl disulfide (26.7 µL, 300 µmol, 3 equiv.), dodecanenitrile (22.15 µL, 100 µmol, 1.0 equiv.) as internal standard, K_2CO_3 (20.8 mg, 150 µmol, 1.5 equiv.) and DIPEA (10.5 µL, 60 µmol, 0.6 equiv.) in ACN 3 mL. The mixture was irradiated for 6 hours, giving an 85% product yield according to GC-FID analysis (79% isolated yield as pale-yellow powder).

^{1}H NMR (400 MHz, $CDCl_3$) δ 7.16 (d, *J* = 3.6 Hz, 1H), 6.37 (d, *J* = 3.6 Hz, 1H), 2.54 (s, 3H), 2.45 (s, 3H) ppm.

^{13}C NMR (100 MHz, $CDCl_3$) δ 185.7 (C, s), 155.0 (C, s), 154.0 (C, s), 119.3 (CH, s), 112.3 (CH, s), 25.9 (CH_3, s), 16.5 (CH_3, s) ppm.
HRMS (EI): m/z $(M+H)^+$ = calcd. for $C_7H_9O_2S$: 157.0311, found: 157.0318.

3v. 1-(5-(Phenylthio)furan-2-yl)ethan-1-one

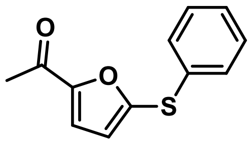

The compound (CAS: 28569-36-0) was prepared according to the general procedure using 2-acetyl-5-bromofuran (19.9 mg, 100 µmol, 1.0 equiv.), diphenyl disulfide (66.2 mg, 300 µmol, 3 equiv.), dodecanenitrile (22.15 µL, 100 µmol, 1.0 equiv.) as internal standard, K_2CO_3 (20.8 mg, 150 µmol, 1.5 equiv.) and DIPEA (10.5 µL, 60 µmol, 0.6 equiv.) in ACN 3 mL. The mixture was irradiated for 6 hours, giving an 81% product yield according to GC-FID analysis (71% isolated yield as brown-yellow powder).

^{1}H NMR (400 MHz, $CDCl_3$) δ 7.41–7.25 (m, 5H), 7.17 (d, *J* = 3.5 Hz, 1H), 6.63 (d, *J* = 3.5 Hz, 1H), 2.45 (s, 3H) ppm.
^{13}C NMR (100 MHz, $CDCl_3$) δ 186.5 (C, s), 155.1 (C, s), 150.6 (C, s), 133.2 (C, s), 130.3 (CH, s), 129.6 (CH, s), 127.9 (CH, s), 118.6 (CH, s), 118.3 (CH, s), 26.1 (CH_3, s) ppm.

Analytical data is in accordance with the previously reported in literature.[3]

3w. Methyl 1-methyl-5-(methylthio)-1*H*-pyrrole-2-carboxylate

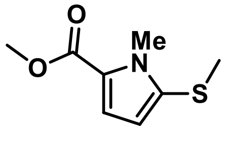

The compound was prepared according to the general procedure using methyl 1-methyl-5-bromo-1*H*-pyrrole-2-carboxylate (21.8 mg, 100 µmol, 1.0 equiv.), dimethyl disulfide (26.7 µL, 300 µmol, 3 equiv.), dodecanenitrile (22.15 µL, 100 µmol, 1.0 equiv.) as internal standard, K_2CO_3 (20.8 mg, 150 µmol, 1.5 equiv.) and DIPEA (10.5 µL, 60 µmol, 0.6 equiv.) in ACN 3 mL. The mixture was irradiated for 24 hours, giving a 50% product yield according to GC-FID analysis (43% isolated yield as pale-yellow oil).

^{1}H NMR (400 MHz, $CDCl_3$) δ 6.97 (d, *J* = 4.3 Hz, 1H), 6.63 (d, *J* = 4.3 Hz, 1H), 4.16 (s, 3H), 3.85 (s, 3H), 2.97 (s, 3H) ppm.
^{13}C NMR (100 MHz, $CDCl_3$) δ 161.2 (C, s), 138.8 (C, s), 127.1 (C, s), 117.1 (CH, s), 109.9 (CH, s), 51.8 (CH_3, s), 39.9 (CH_3, s), 34.1 (CH_3, s) ppm.
HRMS (EI): m/z $(M)^+$ = calcd. for $C_8H_{11}NO_2S$: 185.0505, found: 185.0497.

3x. 1-(5-(Methylthio)selenophen-2-yl)ethan-1-one

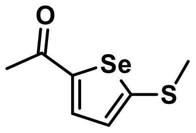

The compound was prepared according to the general procedure using 2-acetyl-5-bromoselenophene (25.2 mg, 100 µmol, 1.0 equiv.), dimethyl disulfide (26.7 µL, 300 µmol, 3 equiv.), dodecanenitrile (22.15 µL, 100 µmol, 1.0 equiv.) as internal standard, K_2CO_3 (20.8 mg, 150 µmol, 1.5 equiv.) and DIPEA (10.5 µL, 60 µmol, 0.6 equiv.) in ACN 3 mL. The mixture was irradiated for 6 hours, giving a 72% product yield according to GC-FID analysis (66% isolated yield as yellow oil).

^{1}H NMR (400 MHz, $CDCl_3$) δ 7.70 (d, *J* = 4.2 Hz, 1H), 7.05 (d, *J* = 4.2 Hz, 1H), 2.61 (s, 3H), 2.50 (s, 3H) ppm.
^{13}C NMR (100 MHz, $CDCl_3$) δ 190.8 (C, s), 157.4 (C, s), 149.2 (C, s), 135.7 (CH, s), 127.4 (CH, s), 25.6 (CH_3, s), 20.6 (CH_3, s) ppm.
HRMS (EI): m/z $(M+H)^+$ = calcd. for C_7H_9OSSe: 220.9534, found: 220.9530.

---
[3] Herrera-Luna, J.C.; *et al. Org. Lett.* **2021**, *23* (6), 2320–2325.

3y. Ethyl 2-(methylthio)oxazole-5-carboxylate

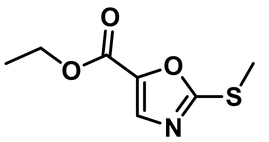

The compound (CAS: 2117579-63-0) was prepared according to the general procedure using ethyl 2-chlorooxazole-5-carboxylate (13.2 µL, 100 µmol, 1.0 equiv.), dimethyl disulfide (26.7 µL, 300 µmol, 3 equiv.), dodecanenitrile (22.15 µL, 100 µmol, 1.0 equiv.) as internal standard, K_2CO_3 (20.8 mg, 150 µmol, 1.5 equiv.) and DIPEA (10.5 µL, 60 µmol, 0.6 equiv.) in ACN 3 mL. The mixture was irradiated for 24 hours, giving a 90% product yield according to GC-FID analysis (74% isolated yield as white powder).

^{1}H NMR (400 MHz, $CDCl_3$) δ 7.69 (s, 1H), 4.36 (q, *J* = 7.1 Hz, 2H), 2.69 (s, 3H), 1.37 (t, *J* = 7.1 Hz, 3H) ppm.
^{13}C NMR (100 MHz, $CDCl_3$) δ 166.2 (C, s), 157.6 (C, s), 144.1 (C, s), 135.4 (CH, s), 61.6 (CH_2, s), 14.7 (CH_3, s), 14.4 (CH_3, s) ppm.
HRMS (EI): m/z $(M+H)^+$ = calcd. for $C_7H_{10}NO_3S$: 188.0376, found 188.0376.

3z. 2-(Methylthio)thiazole-5-carbonitrile

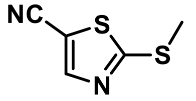

The compound (CAS: 2110748-59-7) was prepared according to the general procedure using 2-bromo-5-thiazolecarbonitrile (18.9 mg, 100 µmol, 1.0 equiv.), dimethyl disulfide (26.7 µL, 300 µmol, 3 equiv.), dodecanenitrile (22.15 µL, 100 µmol, 1.0 equiv.) as internal standard, K_2CO_3 (20.8 mg, 150 µmol, 1.5 equiv.) and DIPEA (10.5 µL, 60 µmol, 0.6 equiv.) in ACN 3 mL. The mixture was irradiated for 2 hours, giving a 91% product yield according to GC-FID analysis (78% isolated yield as white powder).

^{1}H NMR (400 MHz, $CDCl_3$) δ 8.07 (s, 1H), 2.75 (s, 3H) ppm.
^{13}C NMR (100 MHz, $CDCl_3$) δ 174.6 (C, s), 151.9 (CH, s), 111.8 (C, s), 104.6 (C, s), 16.6 (CH_3, s) ppm.
HRMS (EI): m/z $(M+H)^+$ = calcd. for $C_5H_4N_2S_2$: 156.9889, found: 156.9883.

3-Riv. (*S*)-5-(Methylthio)-*N*-((2-oxo-3-(4-(3-oxomorpholino)phenyl)oxazolidin-5-yl)methyl)thiophene-2-carboxamide

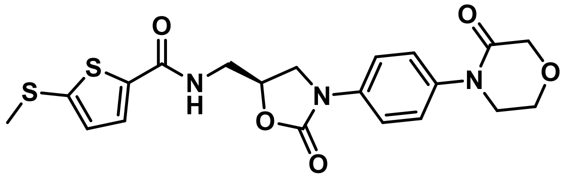

The compound was prepared according to the general procedure using (*S*)-5-chloro-*N*-((2-oxo-3-(4-(3-oxomorpholino)phenyl) oxazolidin-5-yl)methyl)thiophene-2-carboxamide (43.6 mg, 100 µmol, 1.0 equiv.), dimethyl disulfide (26.7 µL, 300 µmol, 3 equiv.), dodecanenitrile (22.15 µL, 100 µmol, 1.0 equiv.) as internal standard, K_2CO_3 (20.8 mg, 150 µmol, 1.5 equiv.) and DIPEA (10.5 µL, 60 µmol, 0.6 equiv.) in ACN 3 mL. The mixture was irradiated for 24 hours, giving a 30% isolated product yield as white powder.

^{1}H NMR (400 MHz, $CDCl_3$) δ 7.60–7.52 (m, 2H), 7.37 (d, *J* = 3.9 Hz, 1H), 7.35–7.31 (m, 2H), 6.9 (d, *J* = 3.9 Hz, 1H), 6.50 (t, *J* = 6.2 Hz, 1H), 4.85 (dtd, *J* = 9.9, 6.6, 3.4 Hz, 1H), 4.33 (s, 2H), 4.09 (t, *J* = 9.0 Hz, 1H), 4.03 (dd, *J* = 5.8, 4.3 Hz, 2H), 3.89 – 3.82 (m, 2H), 3.75 (dd, *J* = 11.3, 6.1 Hz, 3H), 2.55 (s, 3H) ppm.
^{13}C NMR (100 MHz, $CDCl_3$) δ 167.0 (C, s), 162.1 (C, s), 154.5 (C, s), 145.8 (C, s), 137.5 (C, s), 137.4 (C, s), 136.8 (C, s), 129.2 (CH, s), 128.5 (CH, s), 126.4 (CH, s), 119.2 (CH, s), 72.1 (CH, s), 68.7 (CH_2, s), 64.3 (CH_2, s), 49.8 (CH_2, s), 47.8 (CH_2, s), 42.5 (CH_2, s), 14.3 (CH_3, s) ppm.
HRMS (EI): m/z $(M+H)^+$ = calcd. for $C_{20}H_{21}N_3O_5S_2$: 448.0995, found: 448.0986.

4a. 1-(4-(Methylthio)phenyl)ethan-1-one

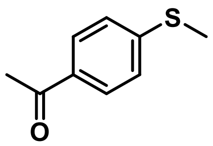

The compound (CAS: 1778-09-2) was prepared according to the general procedure using 1-(4-bromophenyl)ethan-1-one (19.9 mg, 100 µmol, 1.0 equiv.) or 1-(4-chlorophenyl)ethan-1-one (15.5 mg, 100 µmol, 1.0 equiv.), dimethyl disulfide (26.7 µL, 300 µmol, 3 equiv.), dodecanenitrile (22.15 µL, 100 µmol, 1.0 equiv.) as internal standard, K_2CO_3 (41.5 mg, 300 µmol, 3.0 equiv.) and DIPEA (17.4 µL, 100 µmol, 1.0 equiv.) in ACN 3 mL. The mixture was irradiated for 24 hours, giving a 76% (71% from 1-(4-chlorophenyl)ethan-1-one) product yield according to GC-FID analysis (65% isolated yield as pale-yellow powder).

^{1}H NMR (400 MHz, $CDCl_3$) δ 7.87 (d, *J* = 8.6 Hz, 2H), 7.27 (d, *J* = 8.6 Hz, 2H), 2.57 (s, 3H), 2.52 (s, 3H) ppm.
^{13}C NMR (100 MHz, $CDCl_3$) δ 197.3 (C, s), 146.0 (C, s), 133.7 (C, s), 128.9 (CH, s), 125.1 (CH, s), 26.6 (CH_3, s), 14.9 (CH_3, s) ppm.
Analytical data is in accordance with the previously reported in literature.[4]

4b. 2-(Methylthio)-4-(trifluoromethyl)pyridine

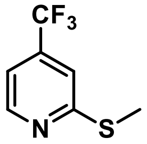

The compound was prepared according to the general procedure using 2-bromo-4-(trifluoromethyl)pyridine (22.6 mg, 100 µmol, 1.0 equiv.), dimethyldisulfide (177.7 µL, 2.0 mmol, 20.0 equiv.), dodecanenitrile (22.15 µL, 100 µmol, 1.0 equiv.) as internal standard, K_2CO_3 (41.5 mg, 300 µmol, 3.0 equiv.) and DIPEA (17.4 µL, 100 µmol, 1.0 equiv.) in ACN 3 mL. The mixture was irradiated for 24 hours, giving an 82% product yield according to GC-FID analysis (39% isolated yield as colorless oil).
Note: This compound is highly volatile and unstable.

^{1}H NMR (400 MHz, CD_2Cl_2) δ 8.59 (d, *J* = 5.2 Hz, 1H), 7.39 (s, 1H), 7.18 (d, *J* = 5.2 Hz, 1H), 2.59 (s, 3H) ppm.
^{19}F-NMR (377 MHz, CD_2Cl_2) δ -65.44 ppm.
HRMS (EI): m/z $(M)^+$ = calcd. for $C_7H_6NSF_3$: 193.0168, found: 193.0160.

4c. 4-(Methylthio)isoquinoline

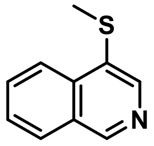

The compound (CAS: 38896-71-8) was prepared according to the general procedure using 4-bromoisoquinoline (20.8 mg, 100 µmol, 1.0 equiv.), dimethyldisulfide (177.7 µL, 2.0 mmol, 20.0 equiv.), dodecanenitrile (22.15 µL, 100 µmol, 1.0 equiv.) as internal standard, K_2CO_3 (41.5 mg, 300 µmol, 3.0 equiv.) and DIPEA (52.3 µL, 300 µmol, 3.0 equiv.) in ACN 3 mL. The mixture was irradiated for 24 hours, giving an 88% product yield according to GC-FID analysis (45% isolated yield as pale-yellow powder).

^{1}H NMR (400 MHz, $CDCl_3$) δ 9.09 (s, 1H), 8.44 (s, 1H), 8.22 (dd, *J* = 8.4, 0.9 Hz, 1H), 7.97 (d, *J* = 8.2, 1H), 7.76 (ddd, *J* = 8.4, 6.9, 1.3 Hz, 1H), 7.65 (ddd, *J* = 8.1, 6.9, 1.1 Hz, 1H), 2.61 (s, 3H). ppm.
^{13}C NMR (100 MHz, $CDCl_3$) δ 150.6 (CH, s), 141.0 (CH, s), 134.7 (C, s), 130.7 (CH, s), 130.6 (C, s), 128.3 (CH, s), 127.8 (CH, s), 123.7 (CH, s), 16.5 (CH_3, s) ppm.
HRMS (ESI): m/z $(M+H)^+$ = calcd. for $C_{10}H_{10}NS$: 176.0529, found: 176.0528.
Analytical data is in accordance with the previously reported in literature.[5]

[4] Ruan, J.; Li,X.; Saidi, O.; Xiao, J. *J. Am. Chem. Soc*. **2008**, *130*, 2424 – 2425.
[5] Wang, Y.; Wu, X.; Yang, M. *Synlett* **2020**, *31*, 1226–1230.

4d. 4-(Methylthio)quinoline

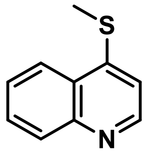

The compound (CAS: 46000-25-3) was prepared according to the general procedure using 4-bromoquinoline (20.8 mg, 100 µmol, 1.0 equiv.), dimethyldisulfide (177.7 µL, 2.0 mmol, 20.0 equiv.), dodecanenitrile (22.15 µL, 100 µmol, 1.0 equiv.) as internal standard, K_2CO_3 (41.5 mg, 300 µmol, 3.0 equiv.) and DIPEA (52.3 µL, 300 µmol, 3.0 equiv.) in ACN 3 mL. The mixture was irradiated for 24 hours, giving a 26% product yield according to GC-FID analysis (20% isolated yield as pale-yellow powder).

^{1}H NMR (400 MHz, $CDCl_3$) δ 8.74 (d, *J* = 4.9 Hz, 1H), 8.10 (ddd, *J* = 7.9, 6.1, 1.6 Hz, 2H), 7.73 (ddd, *J* = 8.4, 6.9, 1.3 Hz, 1H), 7.56 (ddd, *J* = 8.4, 6.9, 1.3 Hz, 1H), 7.14 (d, *J* = 4.9 Hz, 1H), 2.63 (s, 3H) ppm.
^{13}C NMR (100 MHz, $CDCl_3$) δ 149.4 (C, s), 149.2 (CH, s), 147.0 (C, s), 130.0 (CH, s), 129.9 (CH, s), 126.5 (CH, s), 126.4 (C, s), 123.5 (CH, s), 114.9 (CH, s), 14.2 (CH_3, s) ppm.
HRMS (ESI) m/z $(M+H)^+$ = calcd. for $C_{10}H_9NSH$: 176.0529, found: 176.0528.

Analytical data is in accordance with the previously reported in literature.[6]

4e. 3-(Methylthio)quinoline

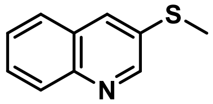

The compound (CAS: 51934-46-4) was prepared according to the general procedure using 3-bromoquinoline (13.9 µL, 100 µmol, 1.0 equiv.), dimethyl disulfide (26.7 µL, 300 µmol, 3 equiv.), dodecanenitrile (22.15 µL, 100 µmol, 1.0 equiv.) as internal standard, K_2CO_3 (20.8 mg, 150 µmol, 1.5 equiv.) and DIPEA (10.5 µL, 60 µmol, 0.6 equiv.) in ACN 3 mL. The mixture was irradiated for 24 hours, giving a 69% product yield according to GC-FID analysis (56% isolated yield as pale-yellow powder).

^{1}H NMR (400 MHz, $CDCl_3$) δ 8.80 (d, *J* = 2.4 Hz, 1H), 8.06 (d, *J* = 8.7 Hz, 1H), 7.90 (d, *J* = 2.2 Hz, 1H), 7.73 (d, *J* = 8.1 Hz, 1H), 7.64 (ddd, *J* = 8.4, 6.9, 1.5 Hz, 1H), 7.57 – 7.51 (m, 1H), 2.61 (s, 3H) ppm.
^{13}C NMR (100 MHz, $CDCl_3$) δ 150.1 (s), 146.1 (s), 132.8 (s), 131.6 (s), 129.5 (s), 128.8 (s), 128.5 (s), 127.4 (s), 126.9 (s), 16.0 (s) ppm.

Analytical data is in accordance with the previously reported in literature.[7]

---

[6] Wang, M.; Qiao, Z.; Zhao, J.; Jiang, X. *Org. Lett.* **2018**, *20* (19), 6193–6197.
[7] Reeves, J.T., Camara, K.; Han, S.Z.; Xu, Y.; Lee, H.; Busacca, C.A., Senanayake, C.H. *Org. Lett.* **2014**, *16* (4), 1196-1199.

NMR Spectra

^{1}HNMR 400MHz, $CDCl_3$

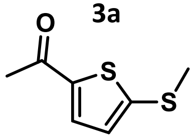

3a

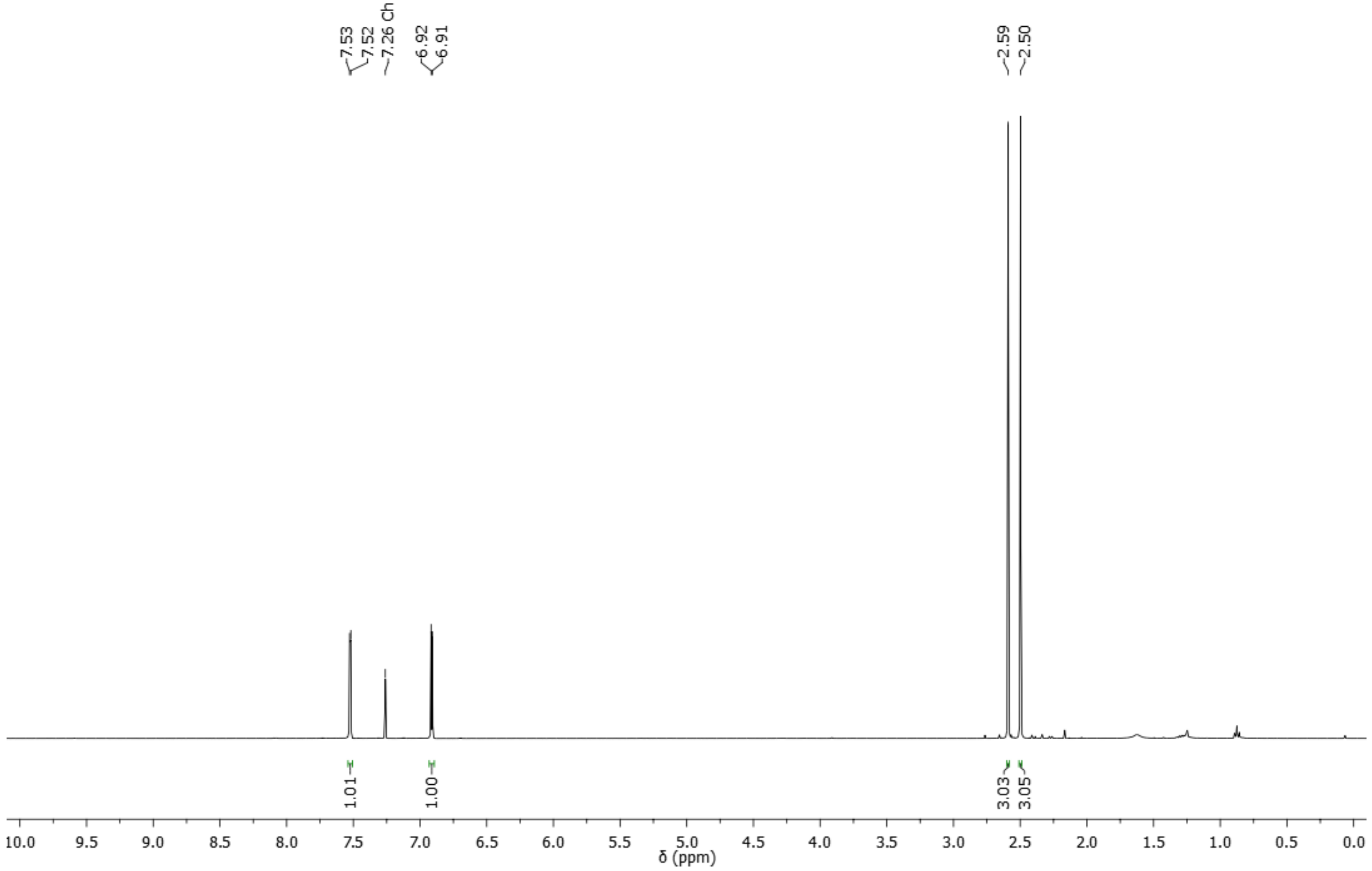

^{13}CNMR 100MHz, $CDCl_3$

3a 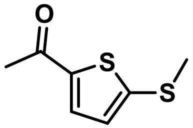

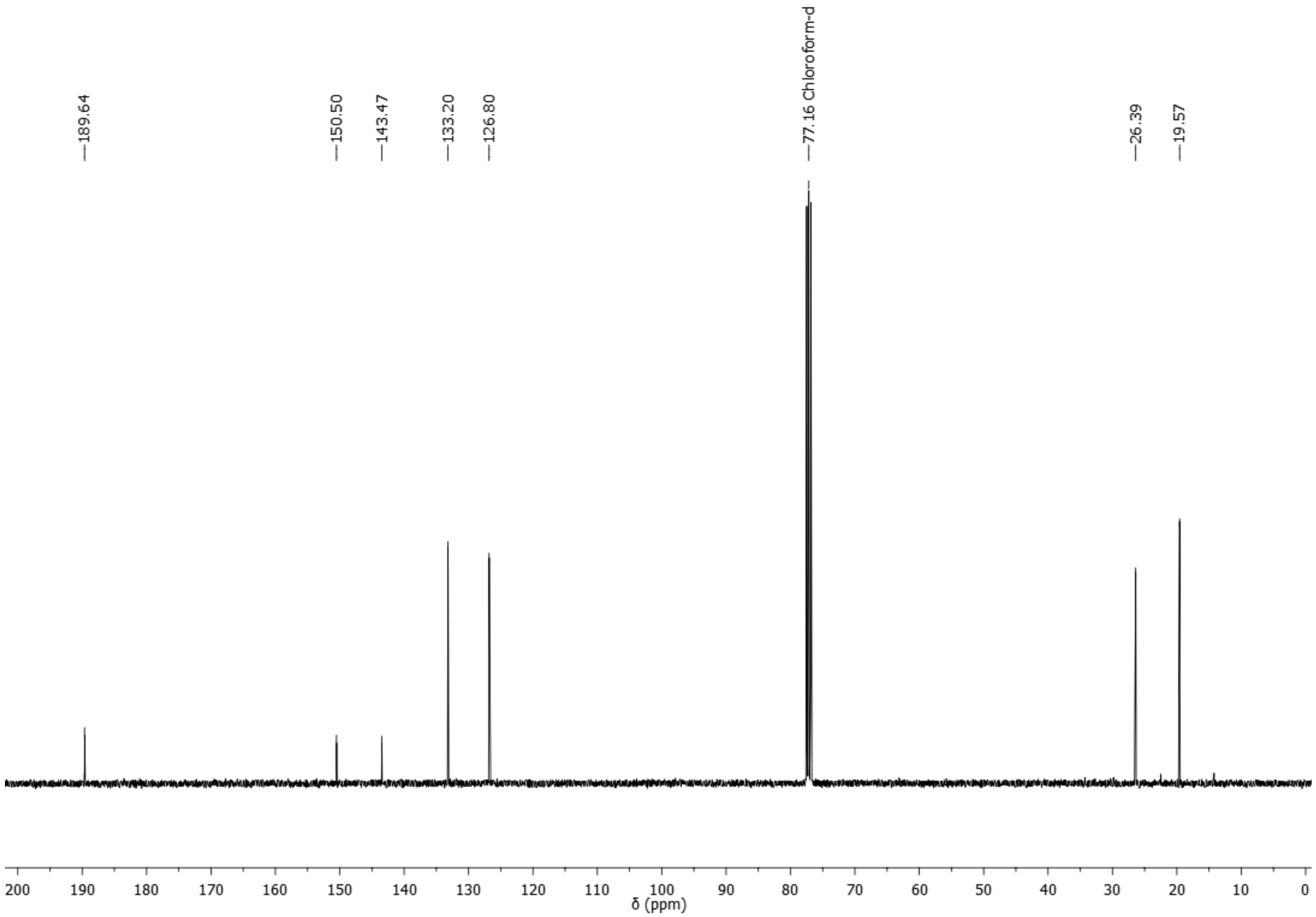

^{1}HNMR 400MHz, $CDCl_3$

3b

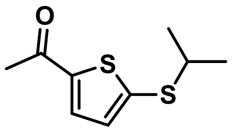

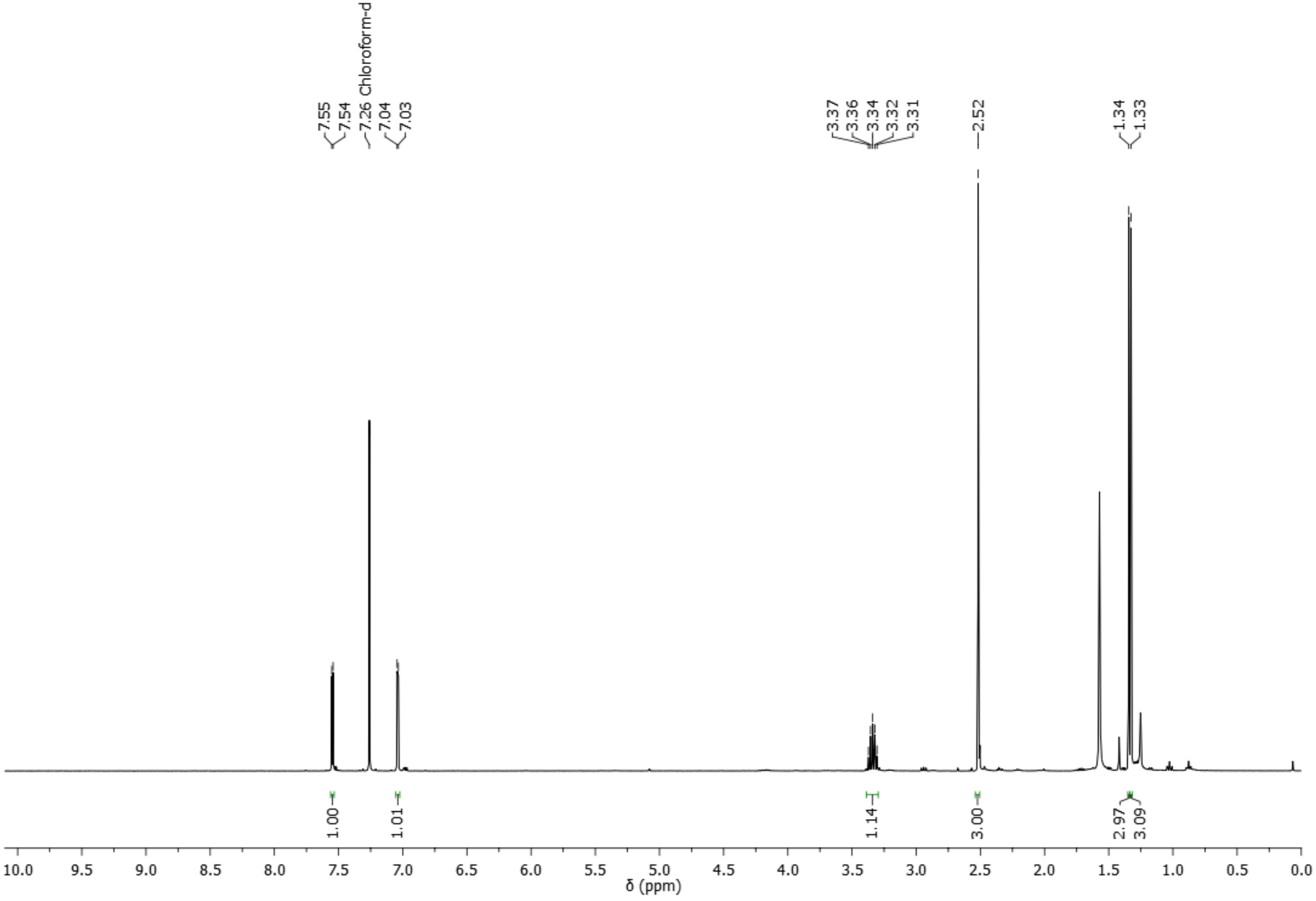

^{13}CNMR 100MHz, $CDCl_3$

3b

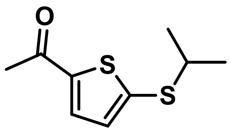

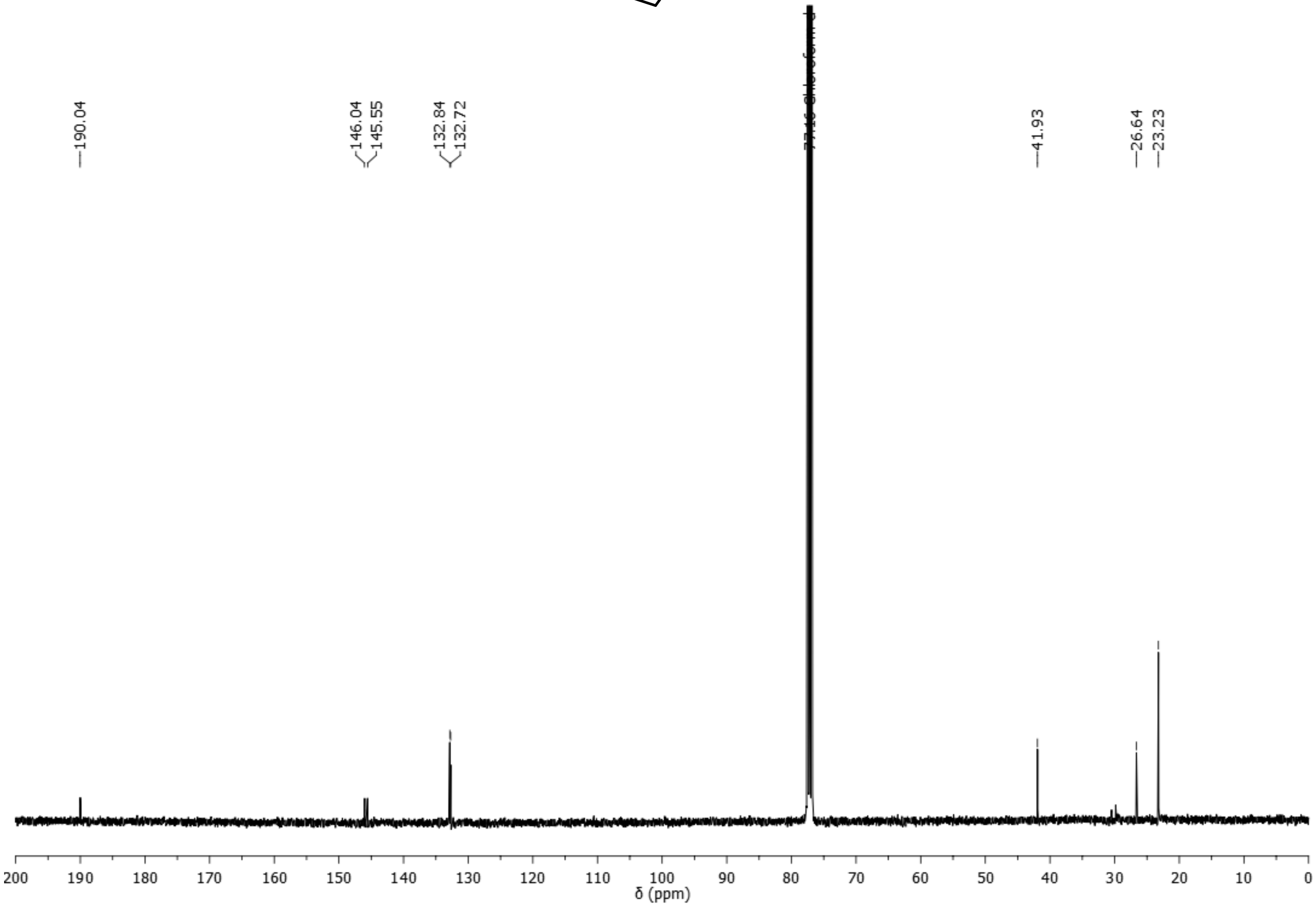

^{1}HNMR 400MHz, $CDCl_3$

3c

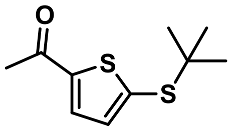

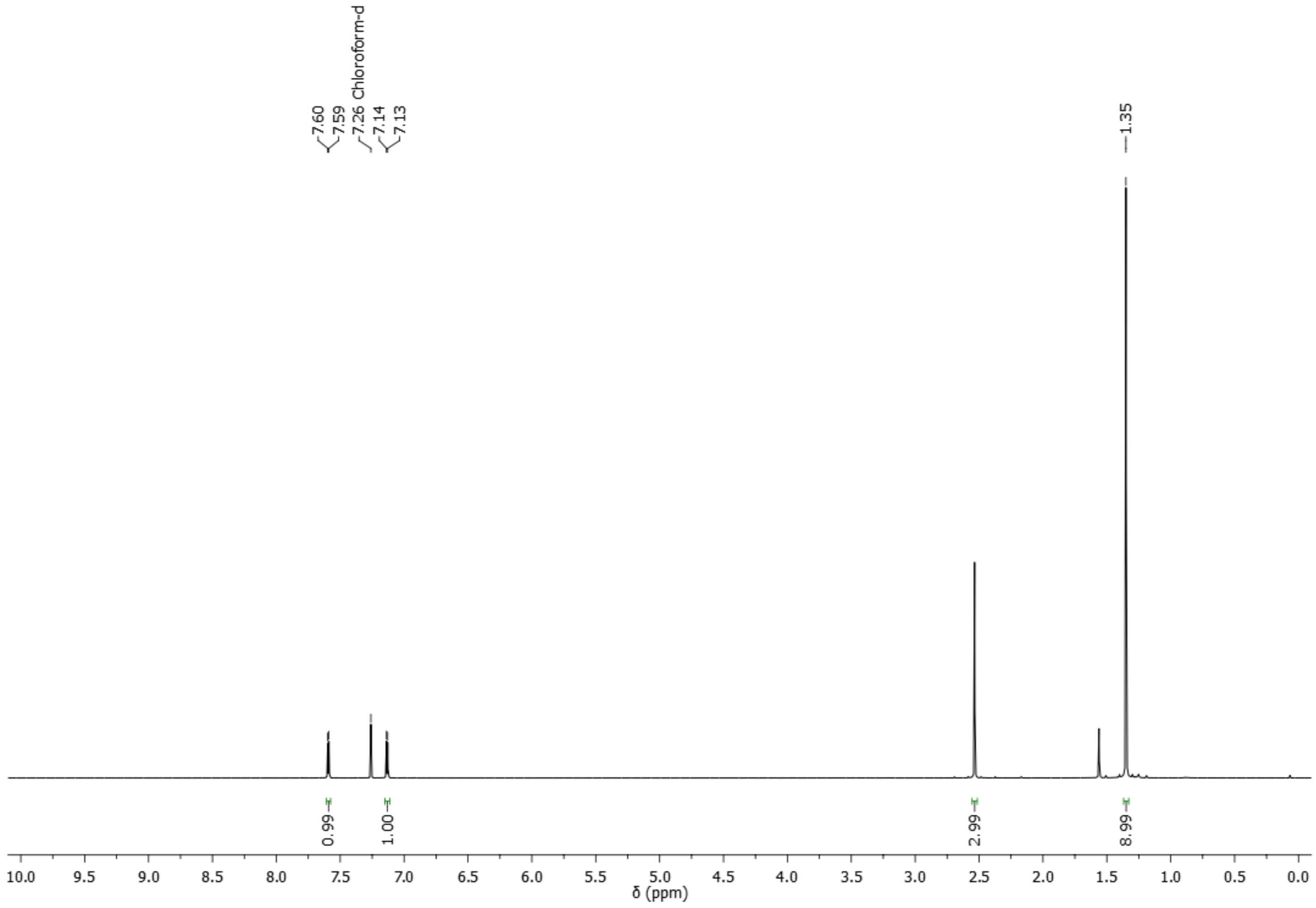

^{13}CNMR 100MHz, $CDCl_3$

3c

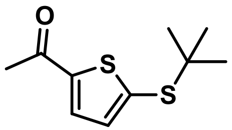

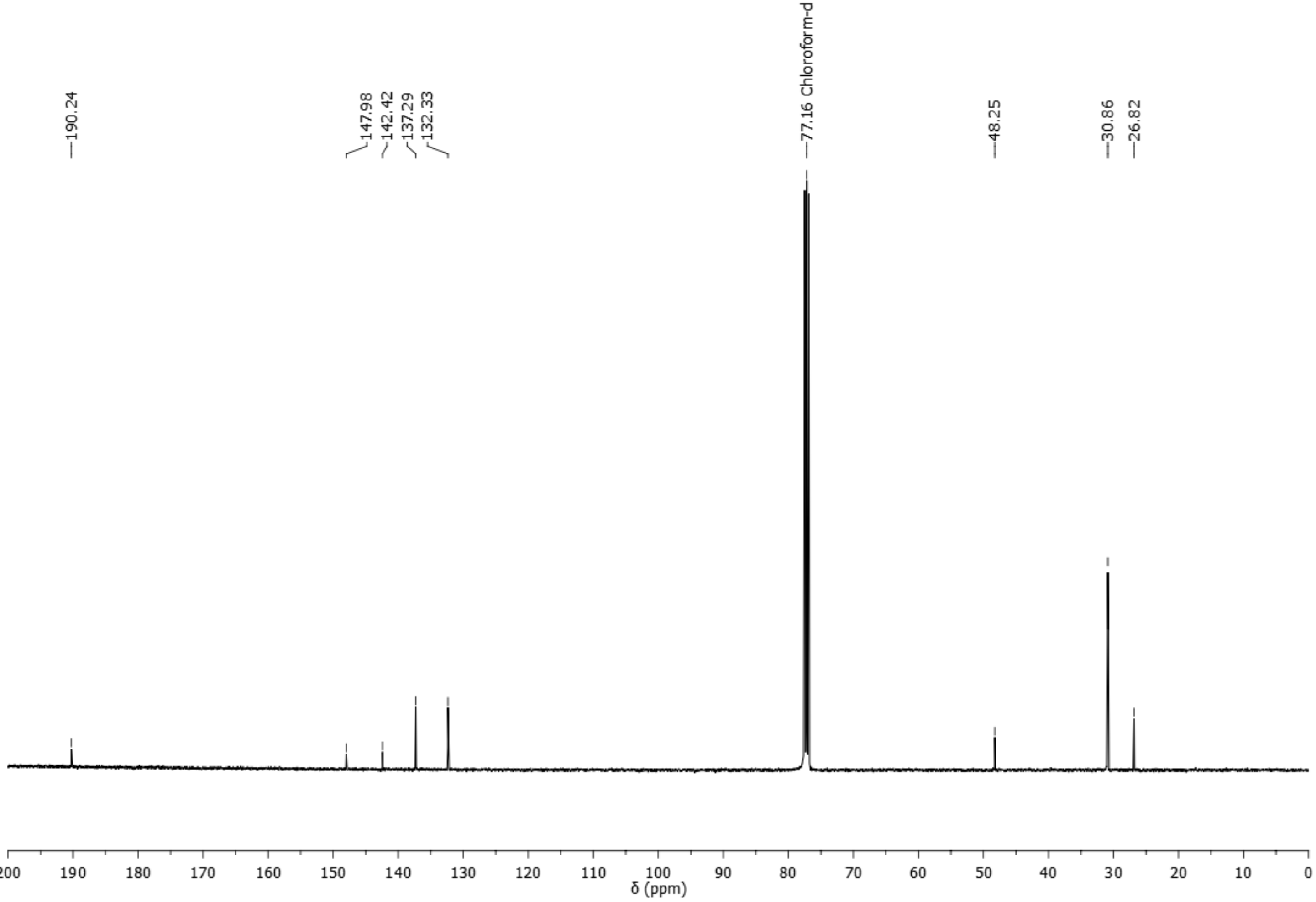

^{1}HNMR 400MHz, $CDCl_3$

3d

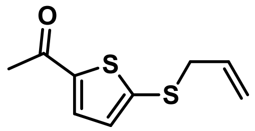

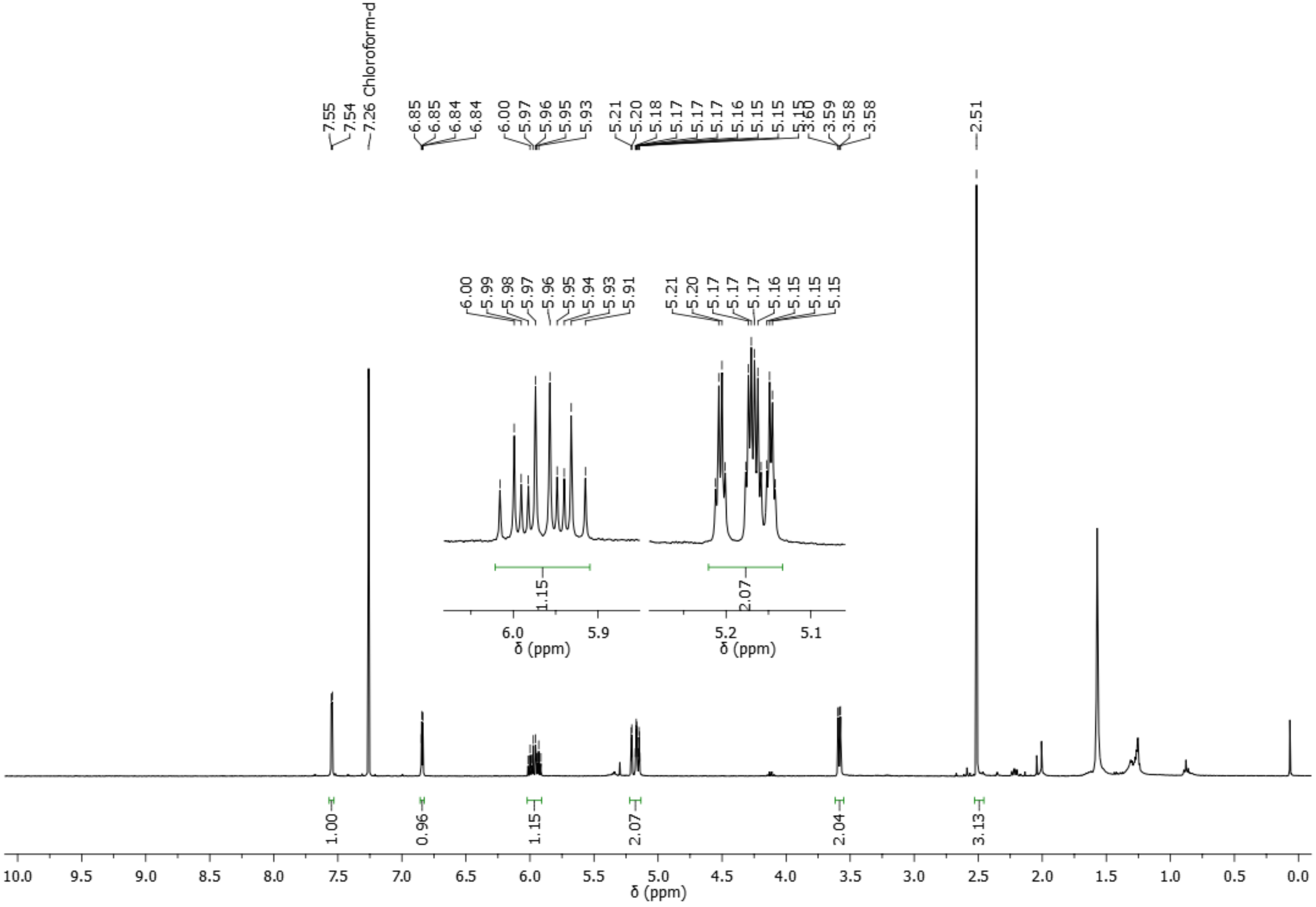

^{13}CNMR 100MHz,

3d

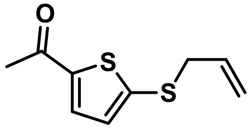

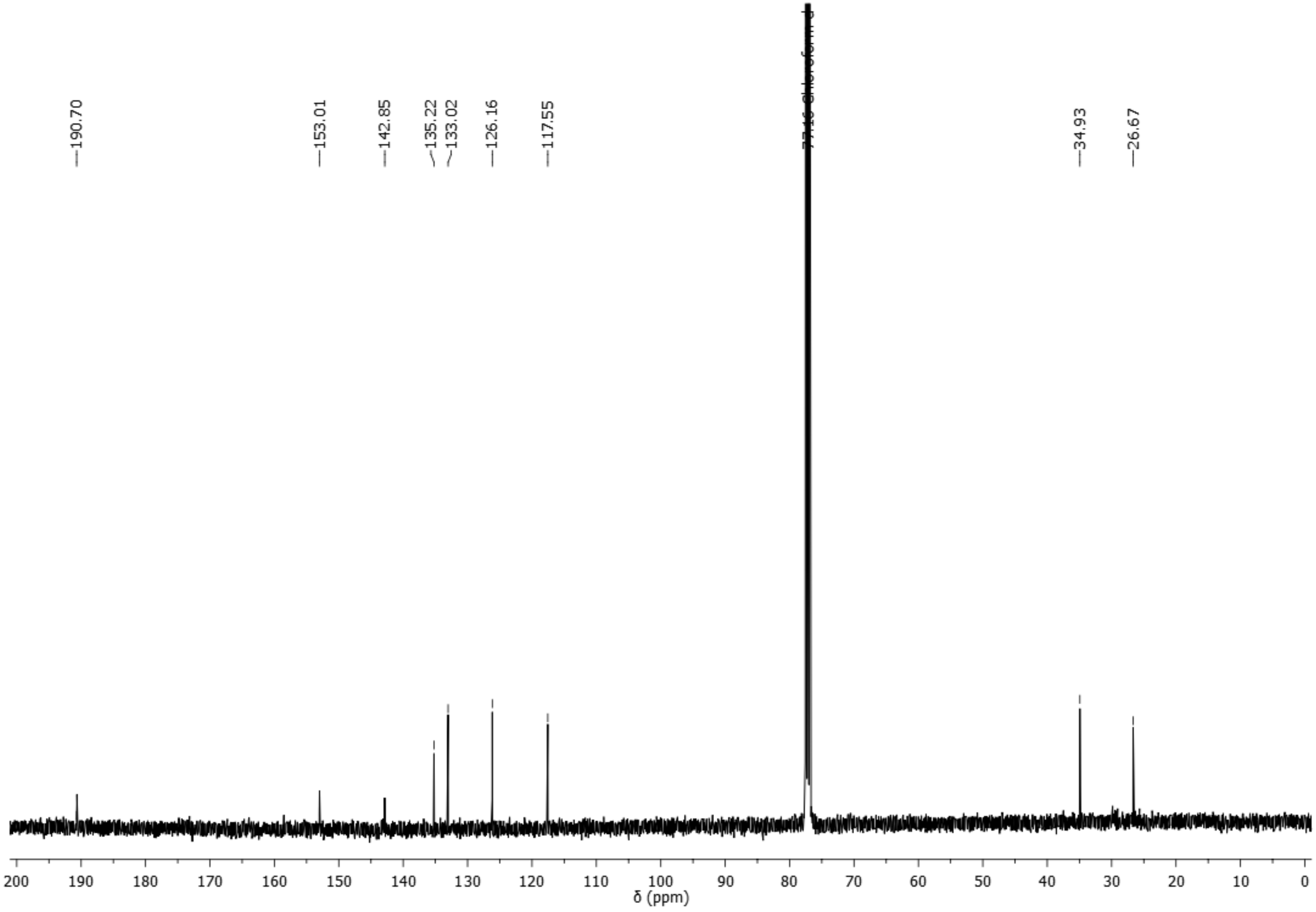

^{1}HNMR 400MHz, $CDCl_3$

3e

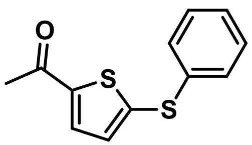

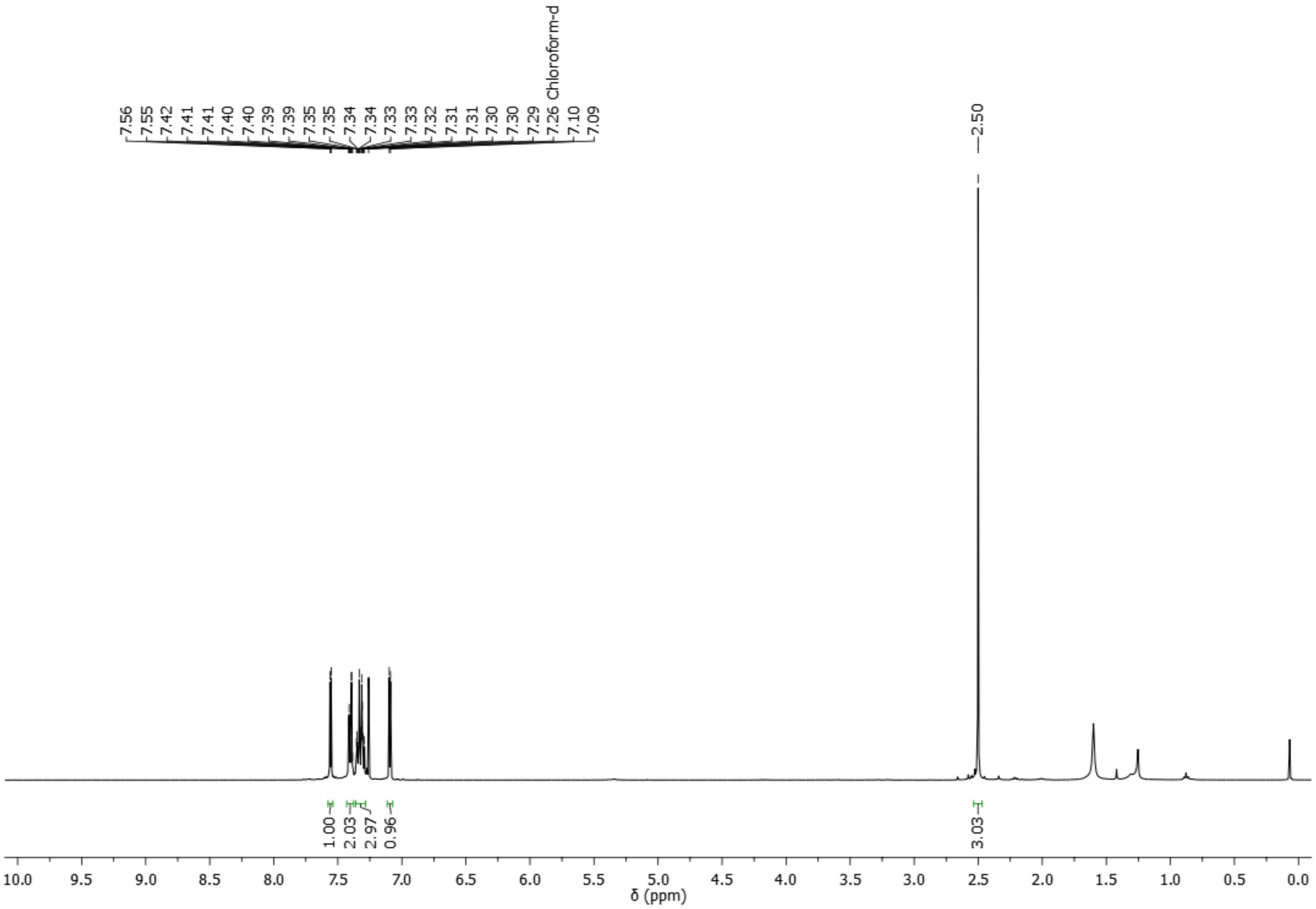

^{13}CNMR 100MHz, $CDCl_3$

3e 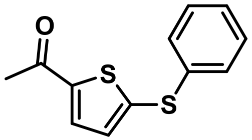

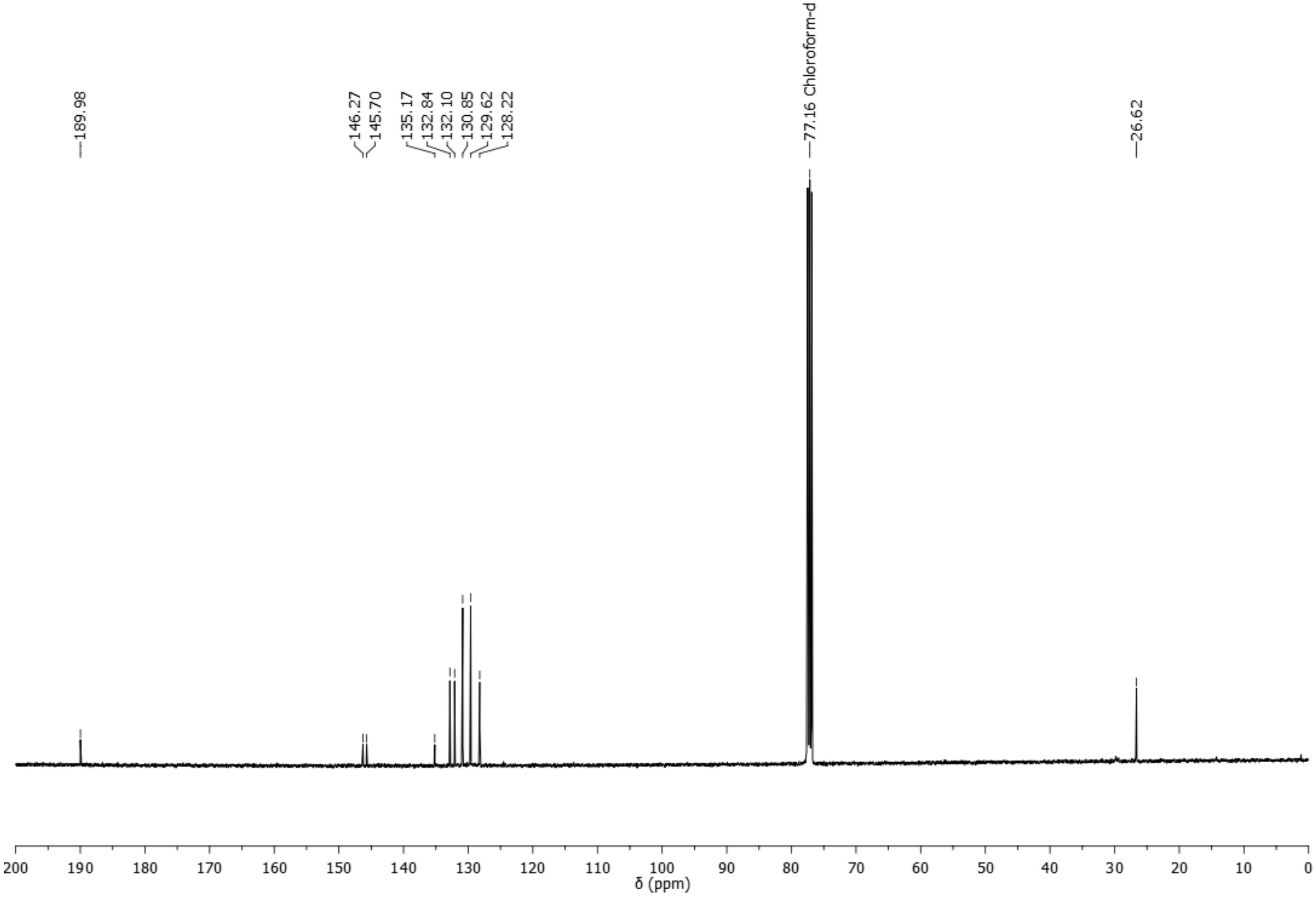

^{1}HNMR 400MHz, $CDCl_3$

3f

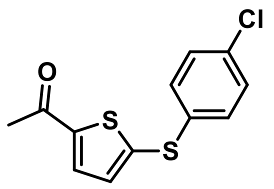

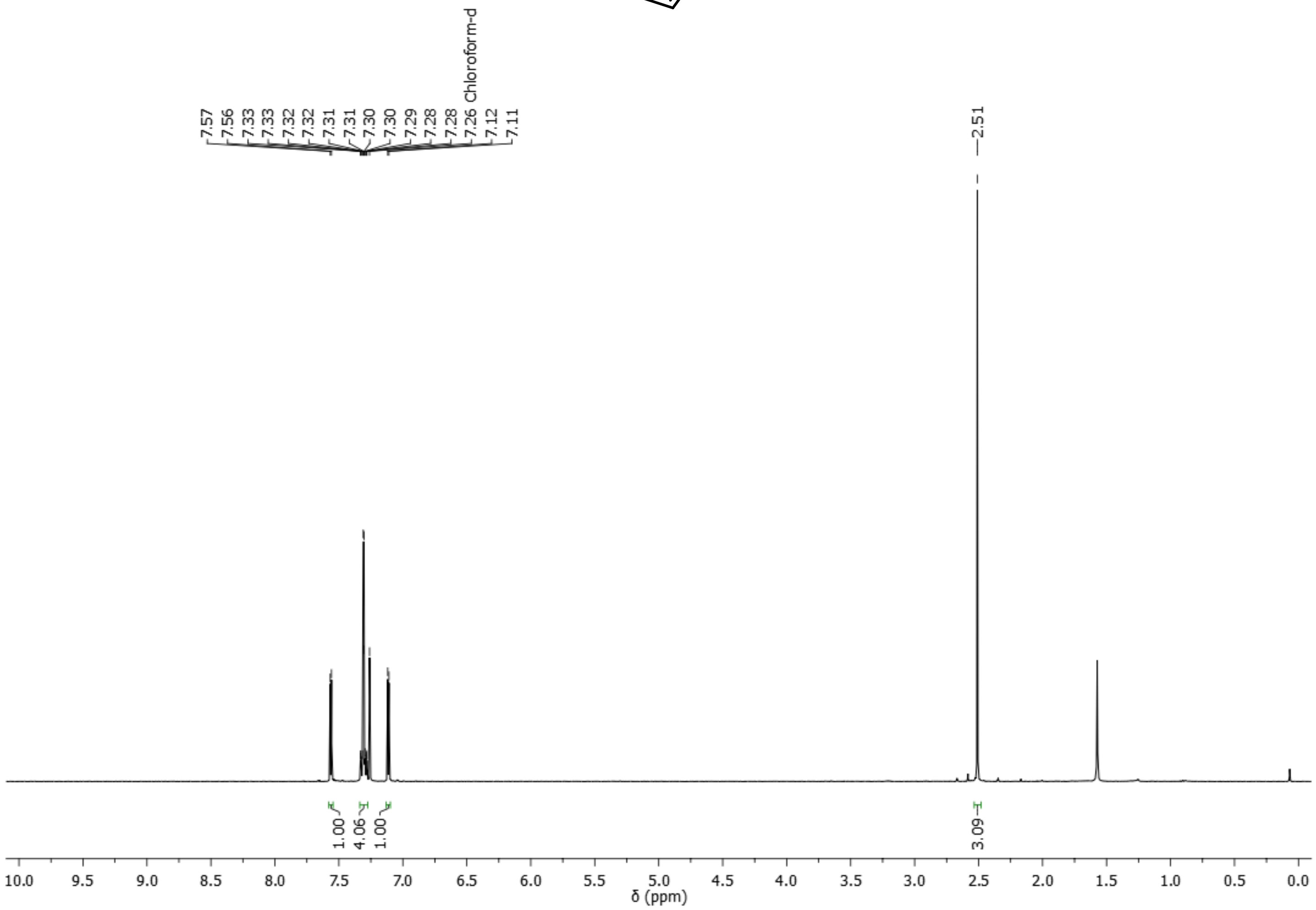

^{13}CNMR 100MHz, $CDCl_3$

3f

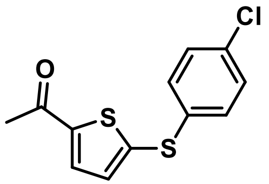

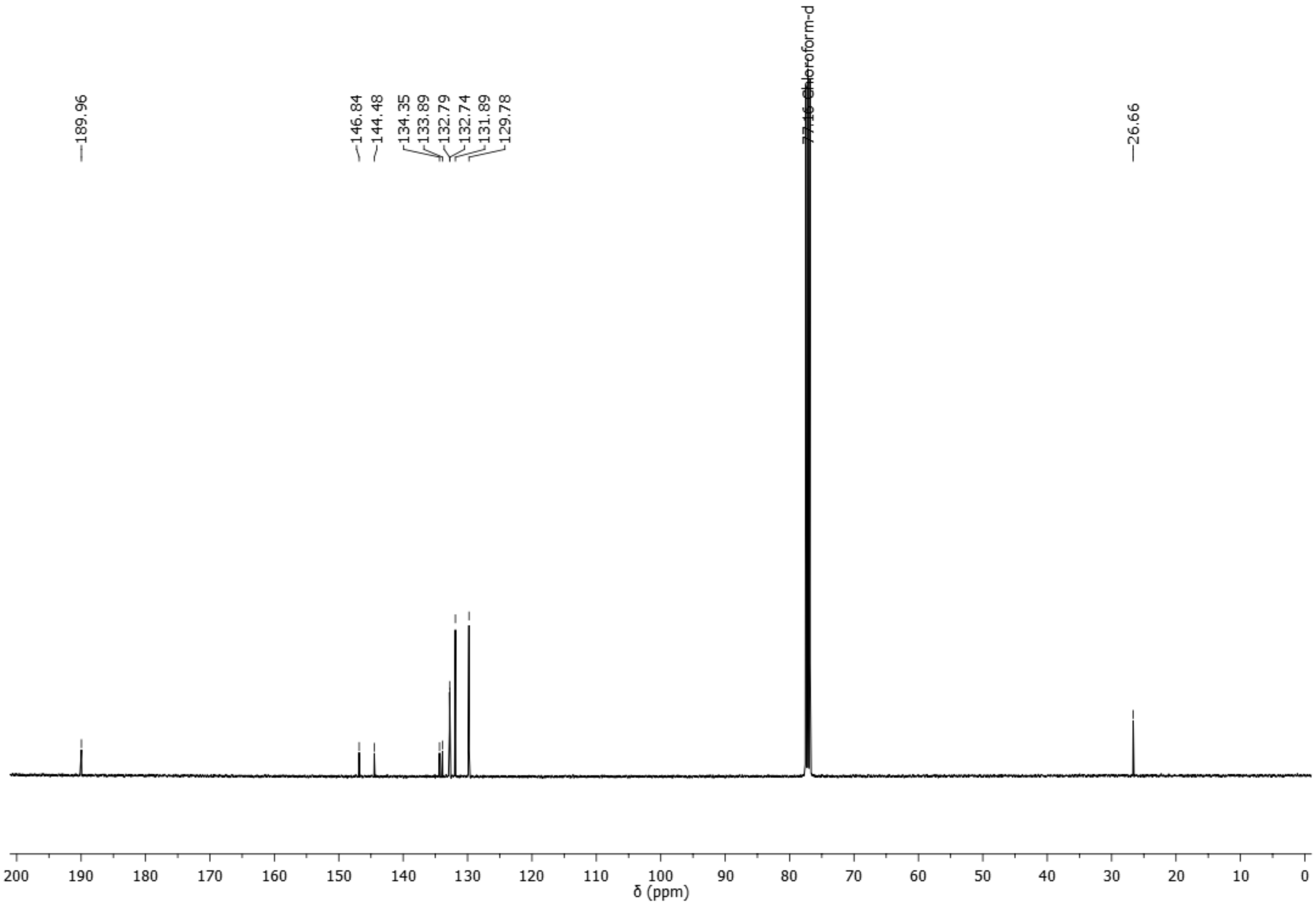

^{1}HNMR 400MHz, CDCl$_3$

3g

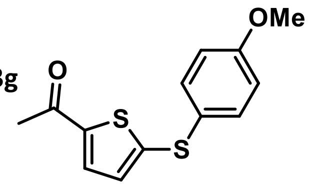

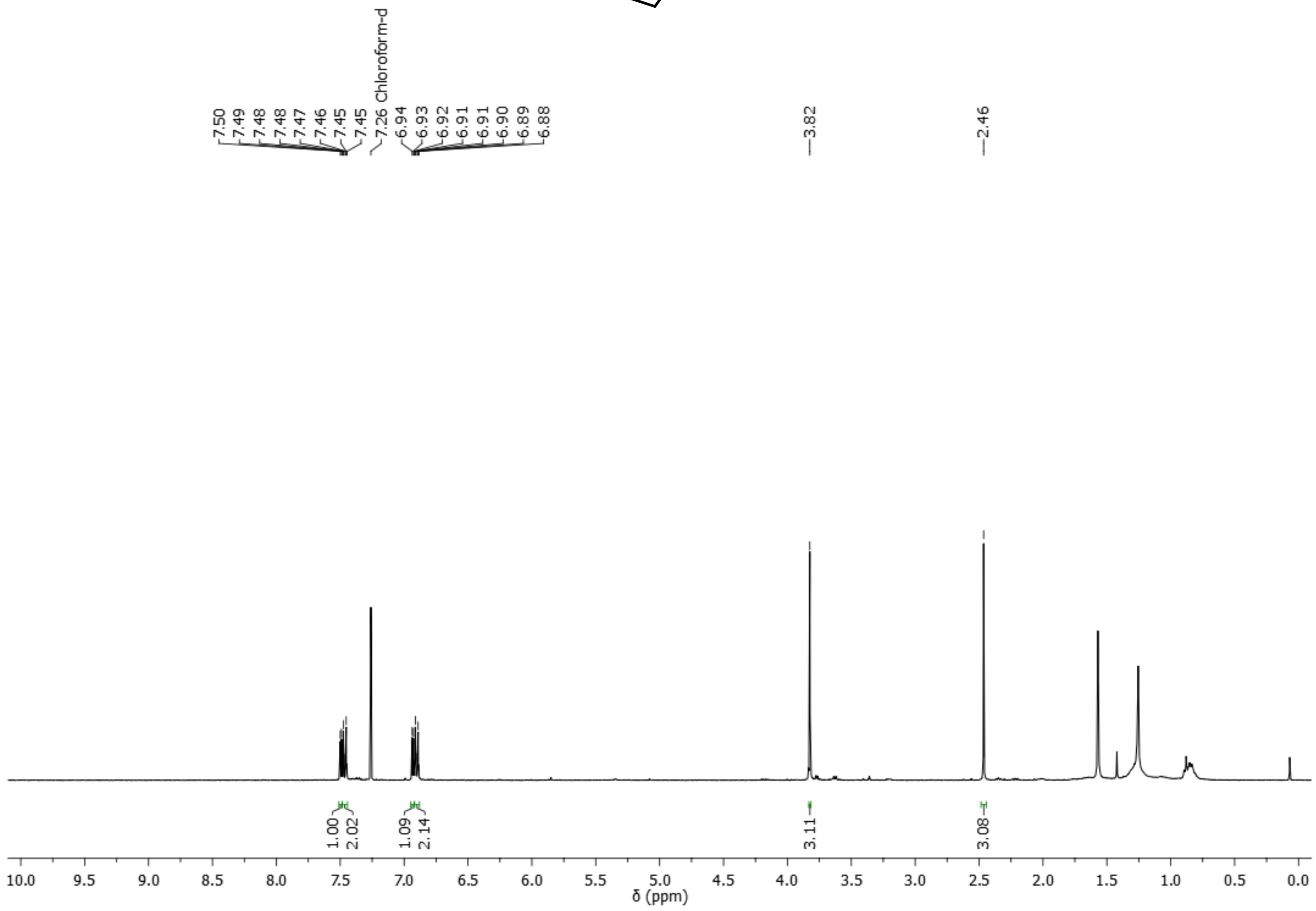

^{13}CNMR 100 MHz, $CDCl_3$

3g

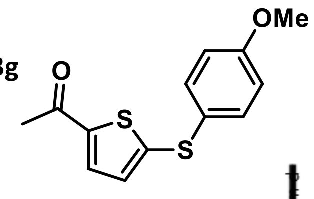

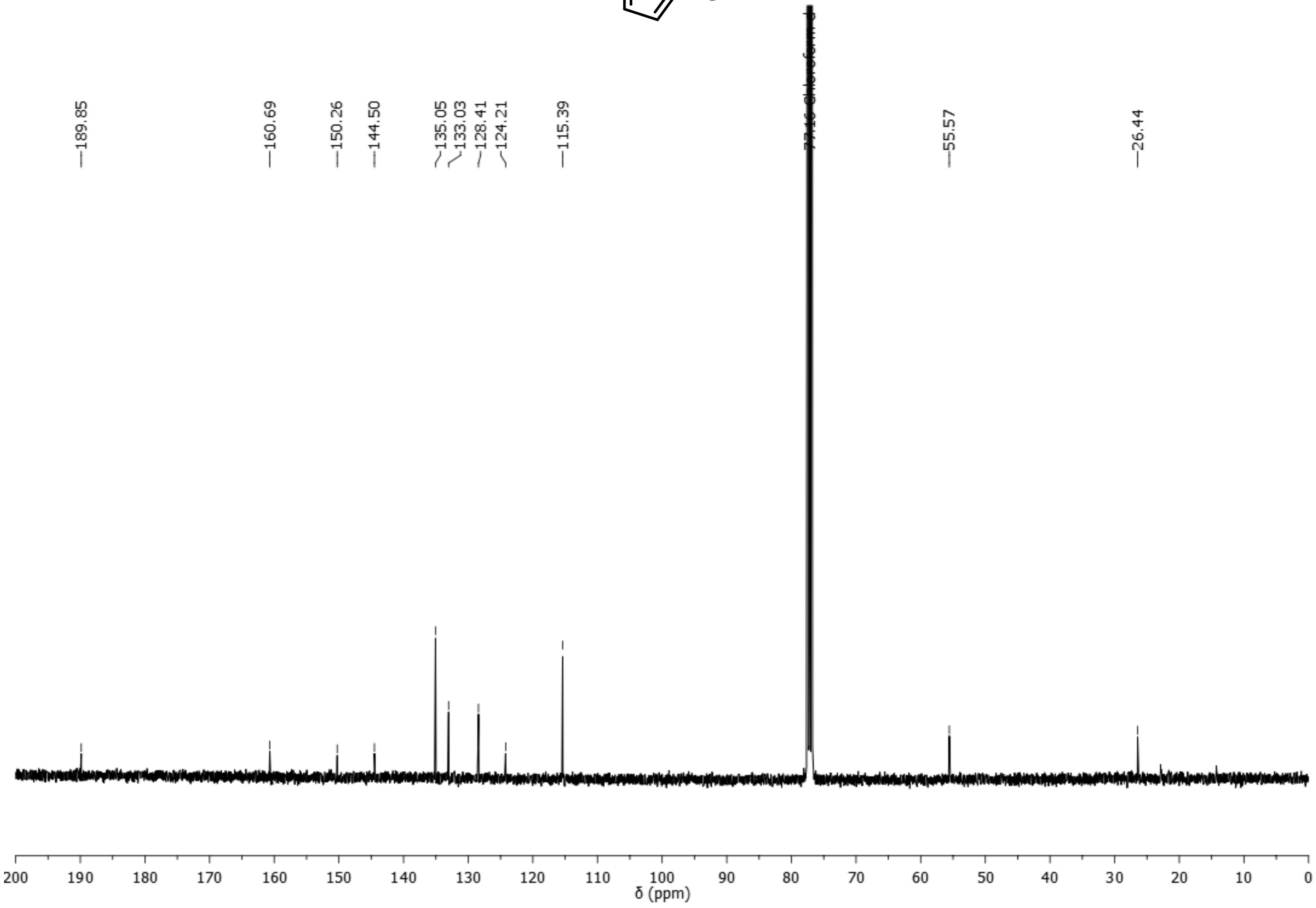

^{1}HNMR 400MHz, $CDCl_3$

3h

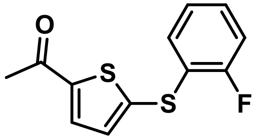

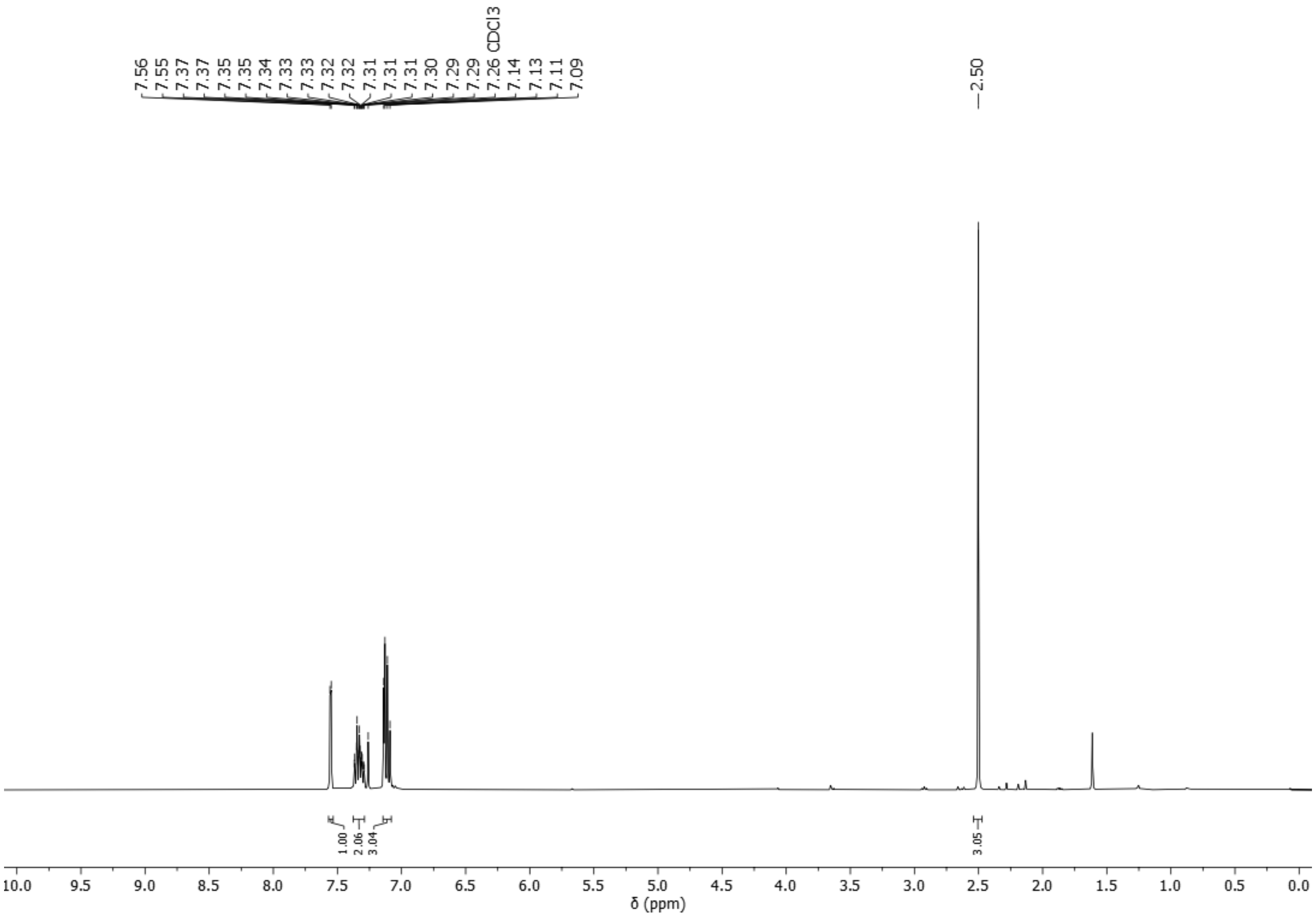

^{13}CNMR 100MHz, $CDCl_3$

3h

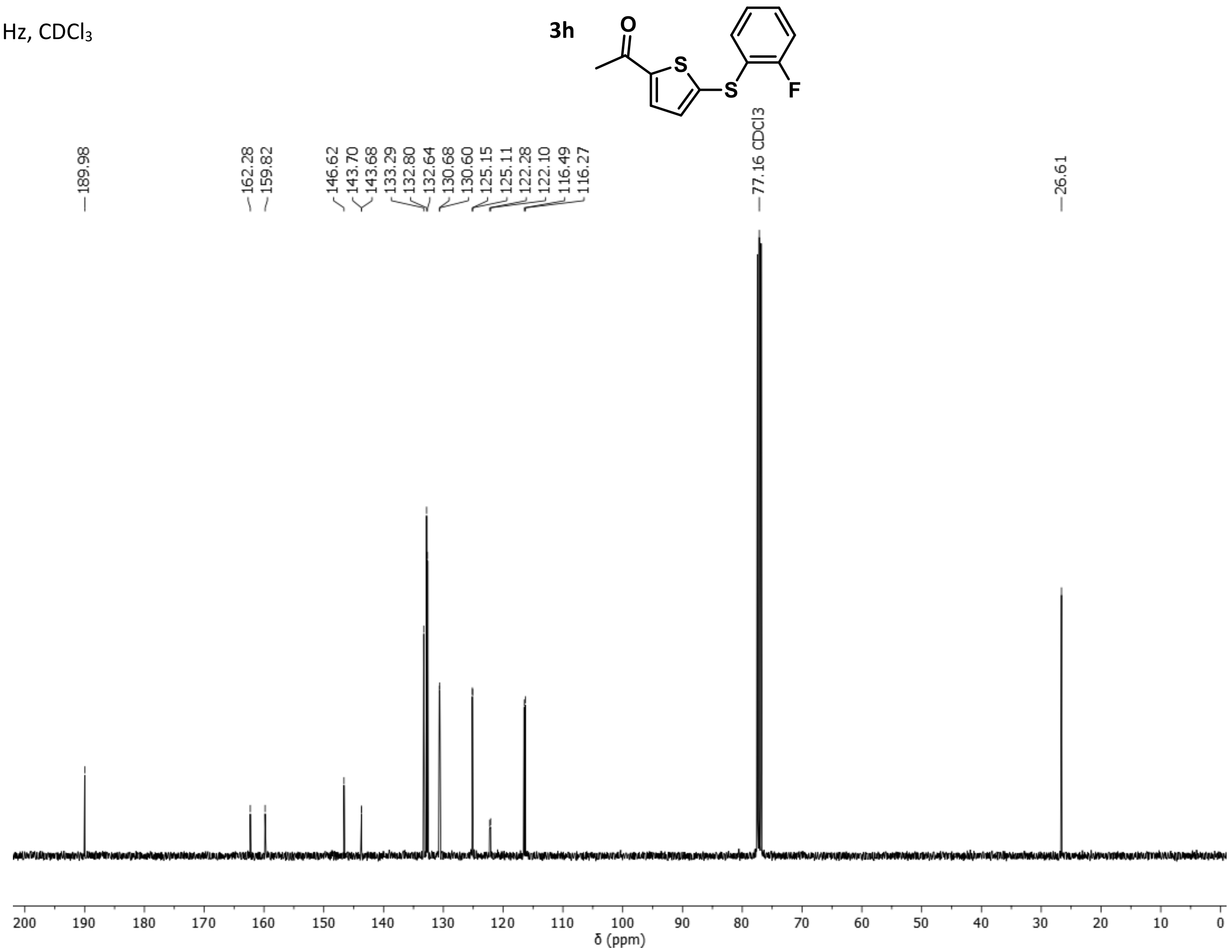

^{19}FNMR 377MHz, $CDCl_3$

3h

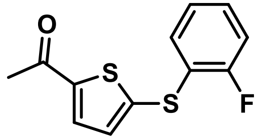

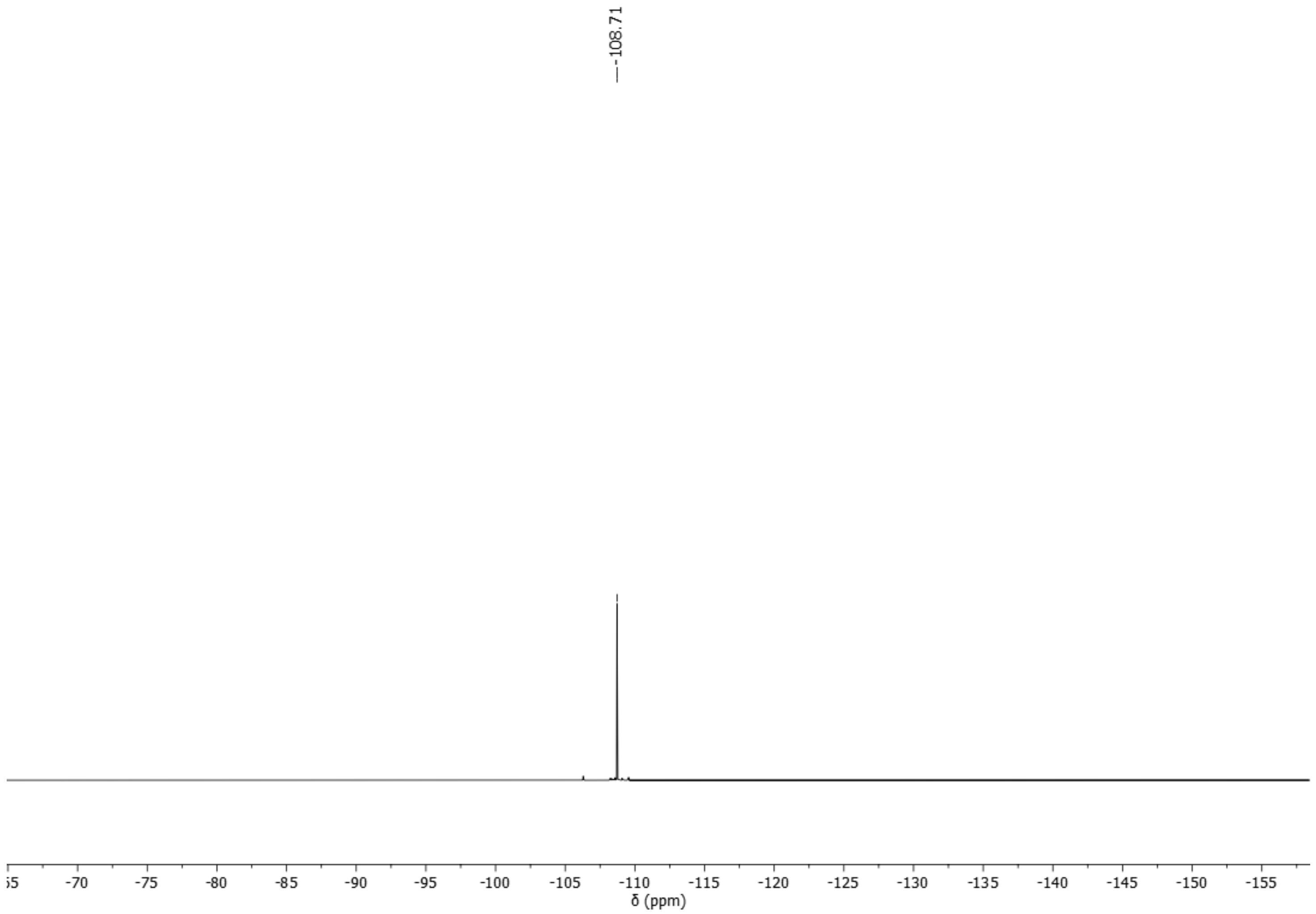

^{1}HNMR 400MHz, $CDCl_3$

3i

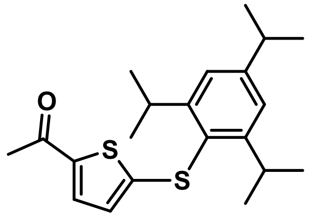

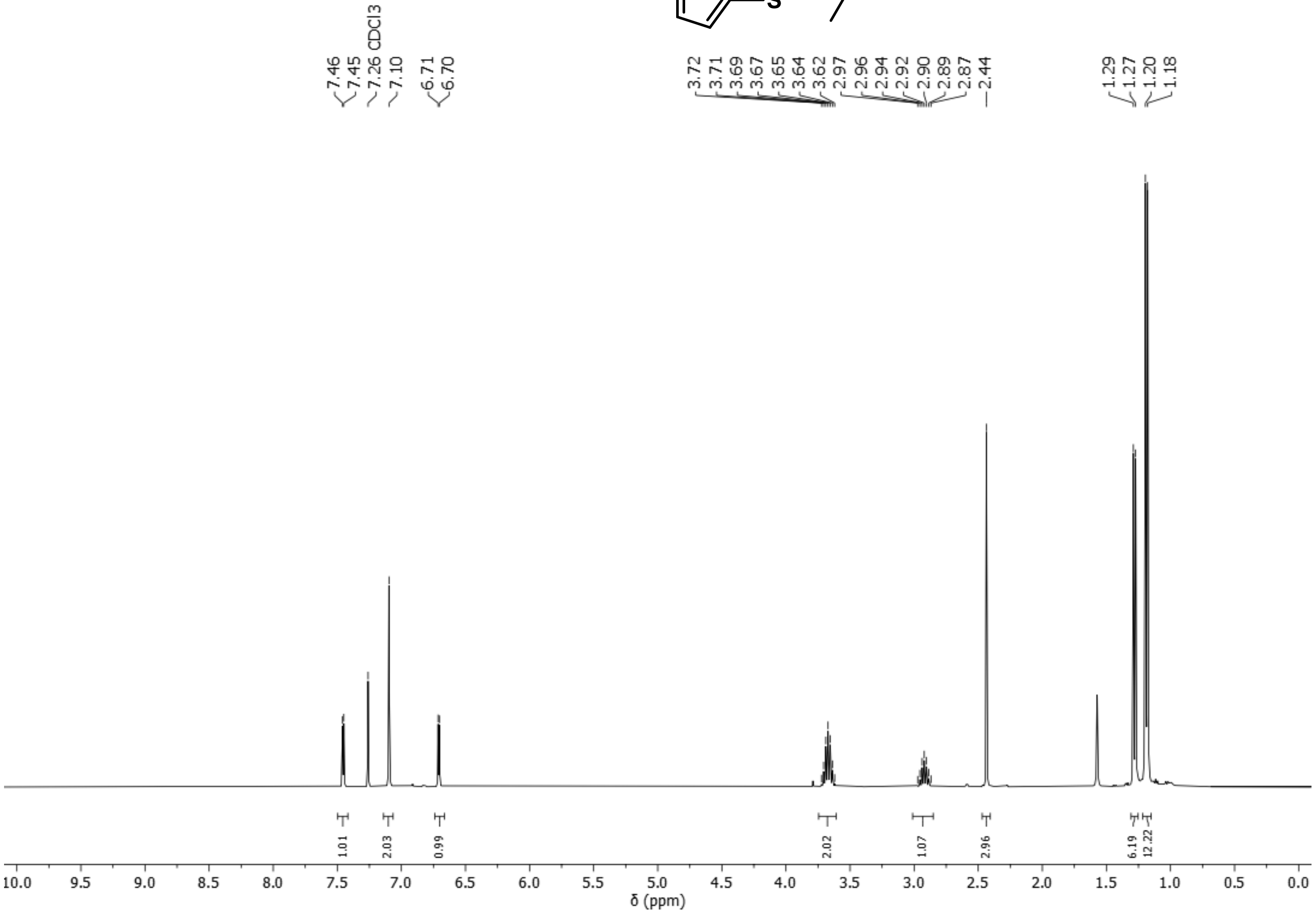

^{13}CNMR 100MHz, $CDCl_3$

3i

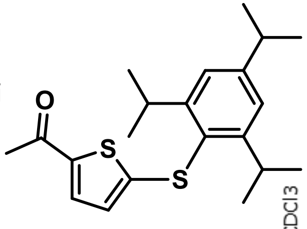

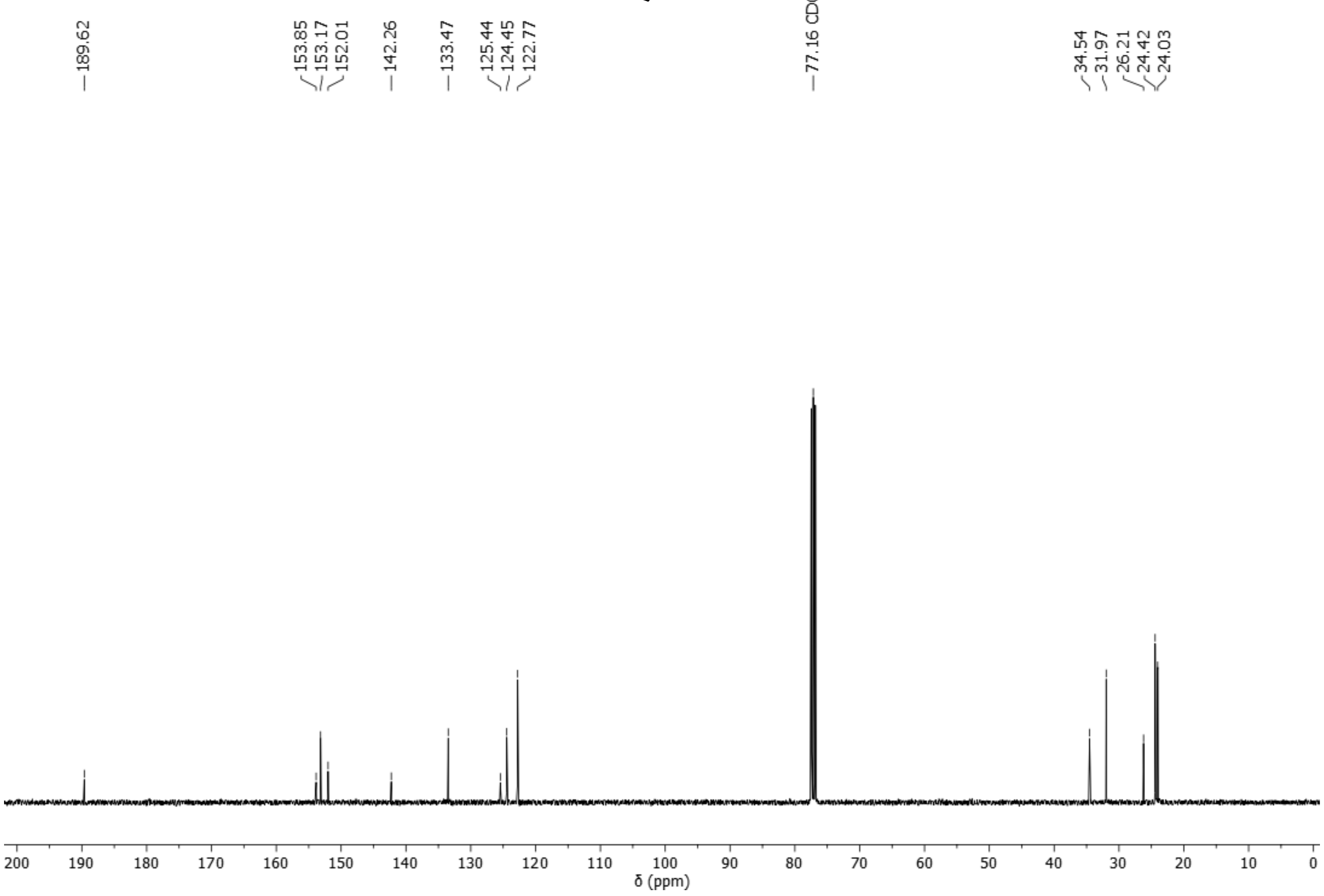

^{1}HNMR 400MHz, $CDCl_3$

3j

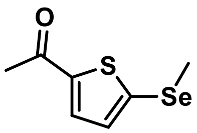

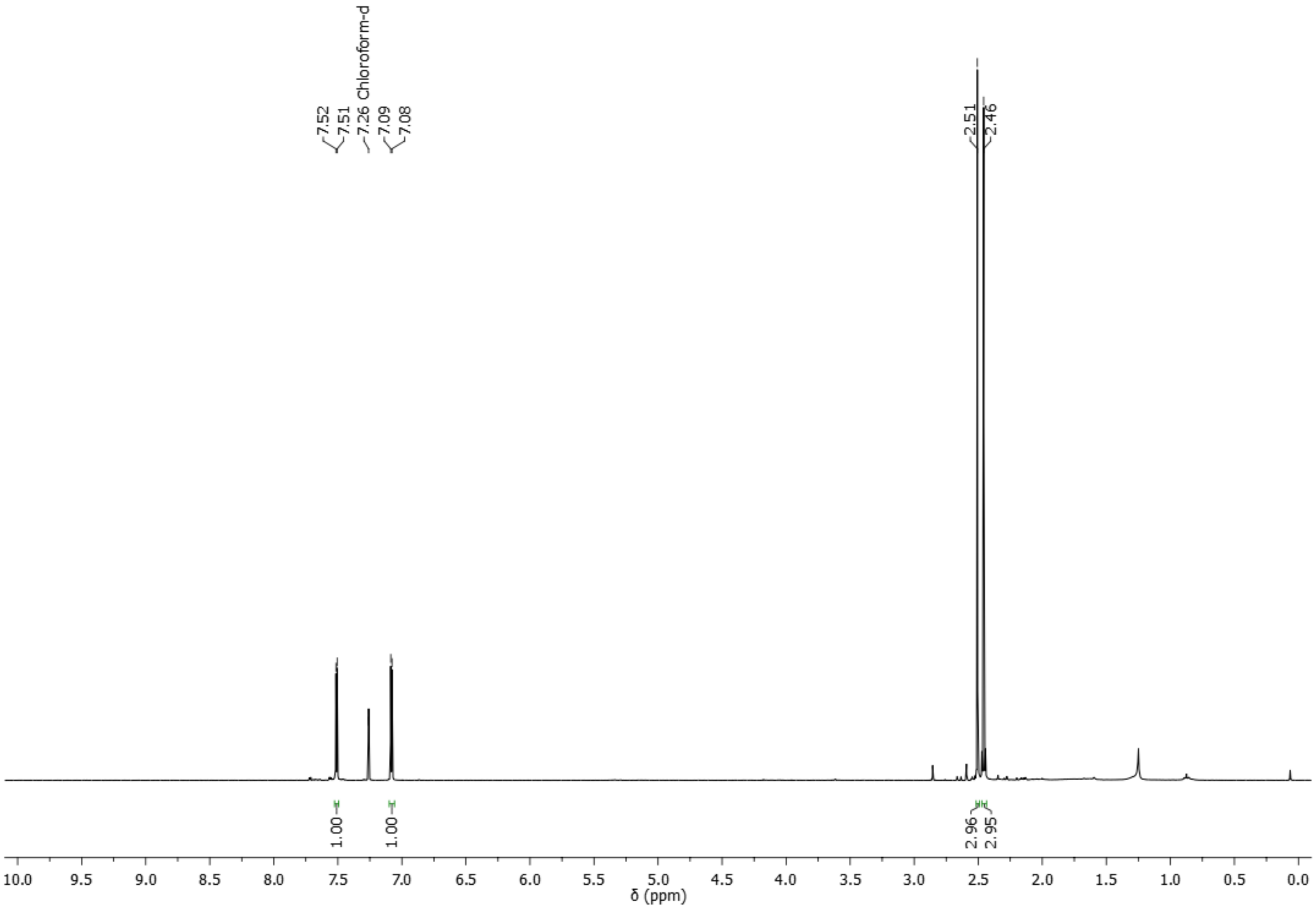

^{13}CNMR 100MHz, $CDCl_3$

3j

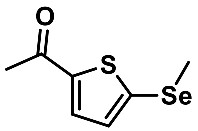

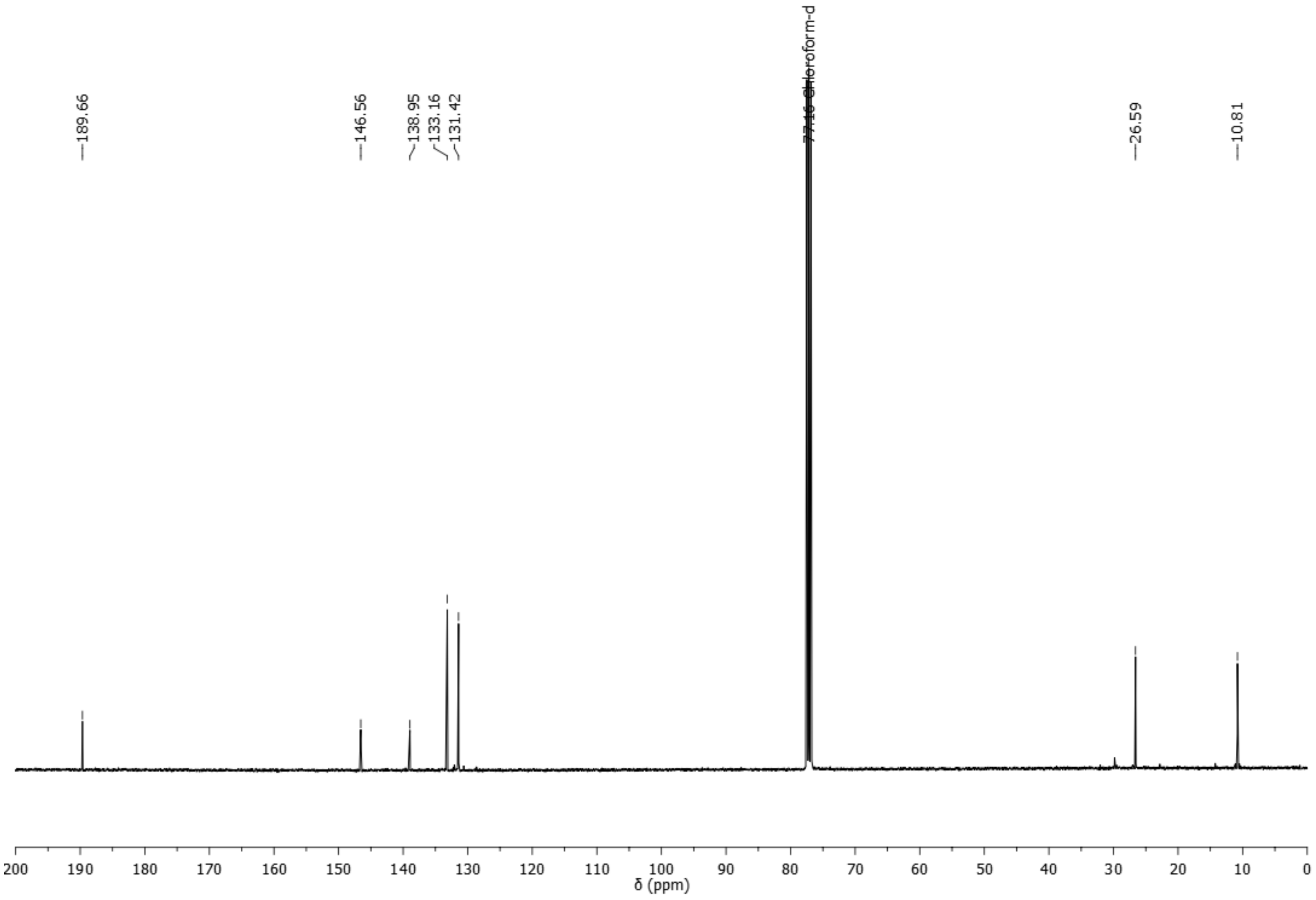

^{1}HNMR 400MHz, $CDCl_3$

3k

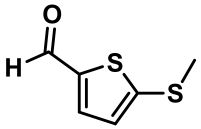

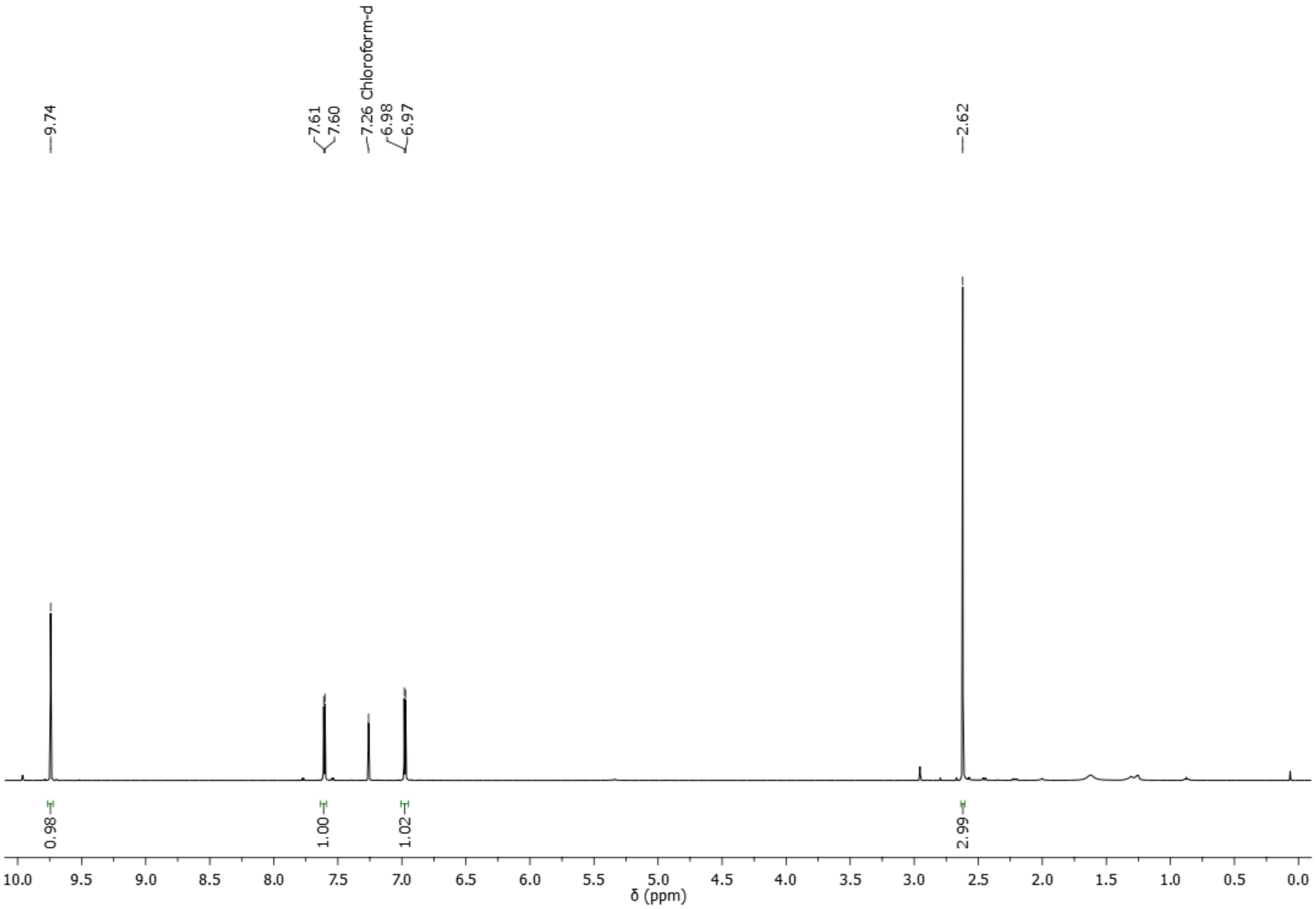

^{13}CNMR 100MHz, $CDCl_3$

3k

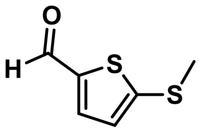

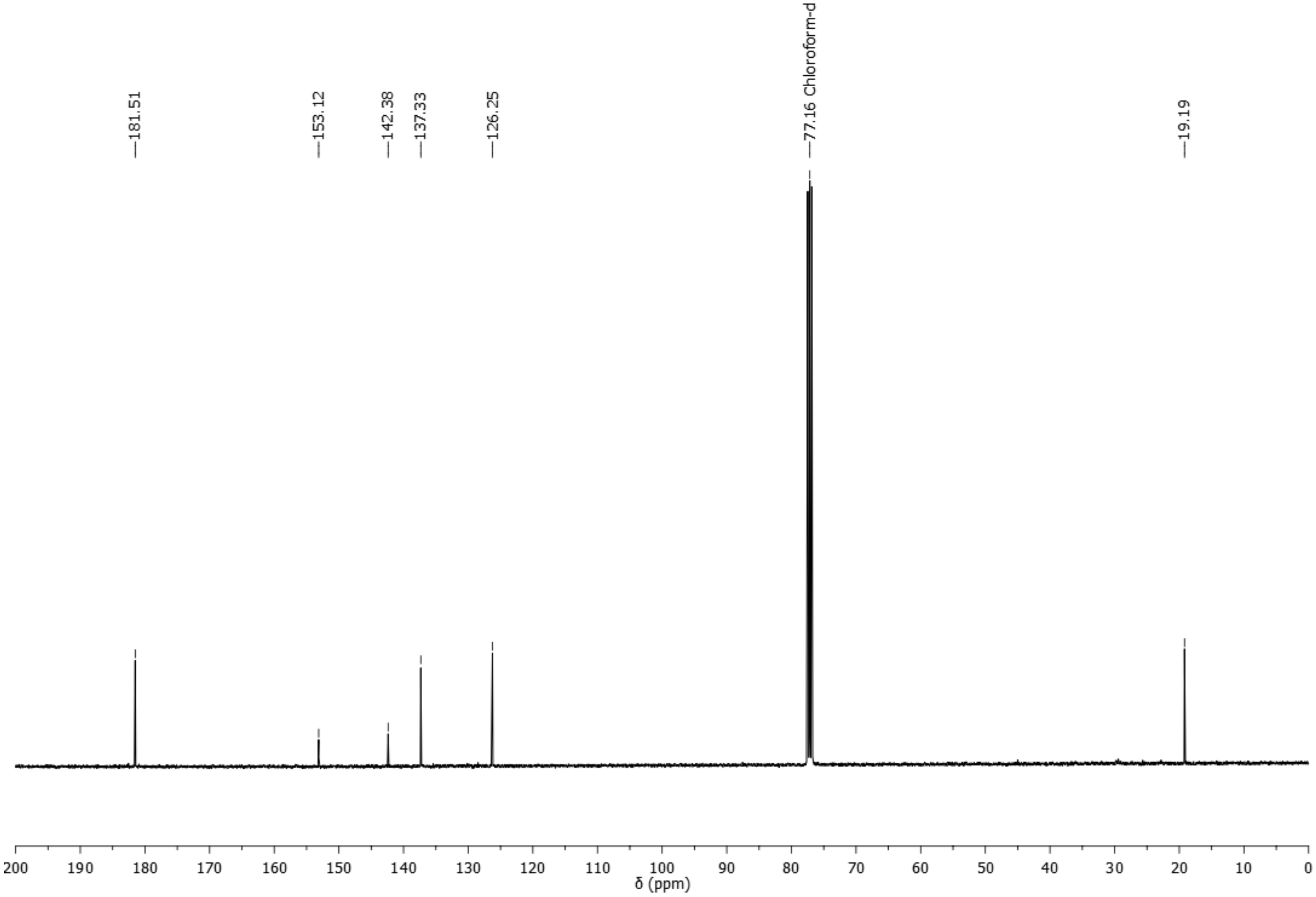

^{1}HNMR 400MHz, $CDCl_3$

3l

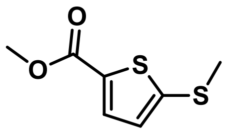

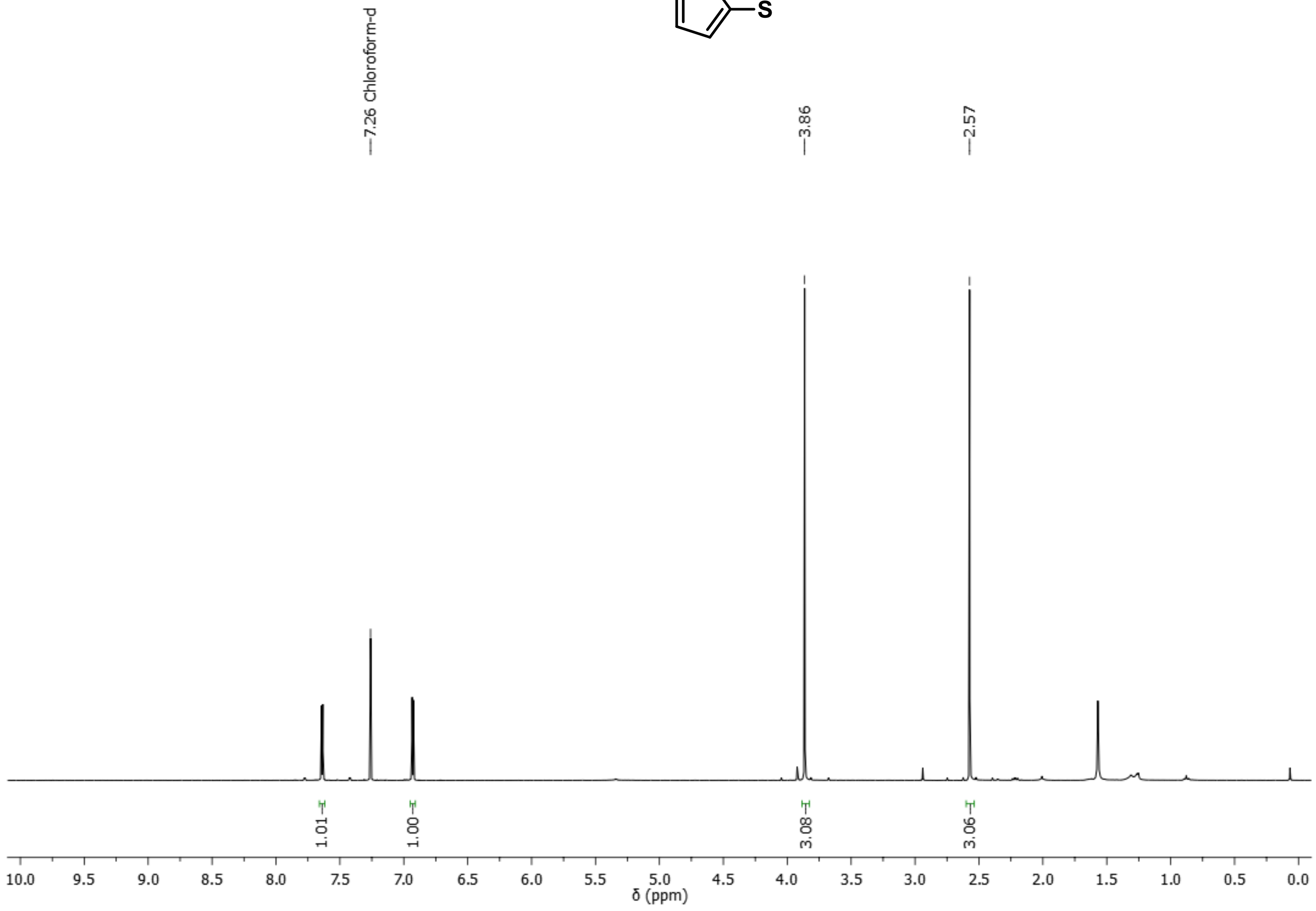

^{13}CNMR 100MHz, $CDCl_3$

3l

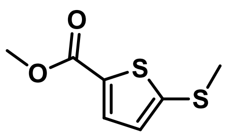

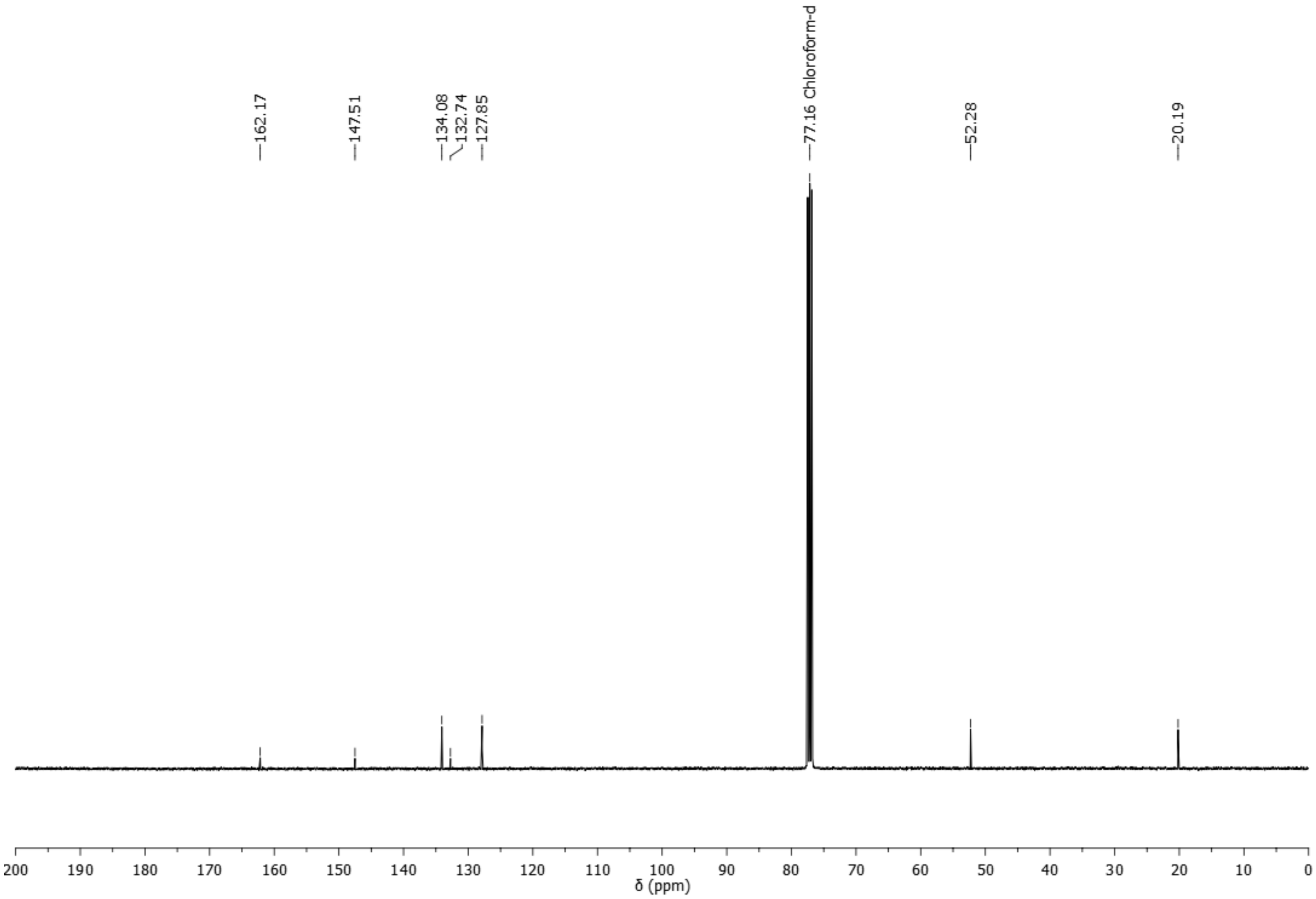

^{1}HNMR 400MHz, $CDCl_3$

3m

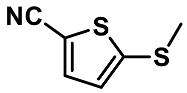

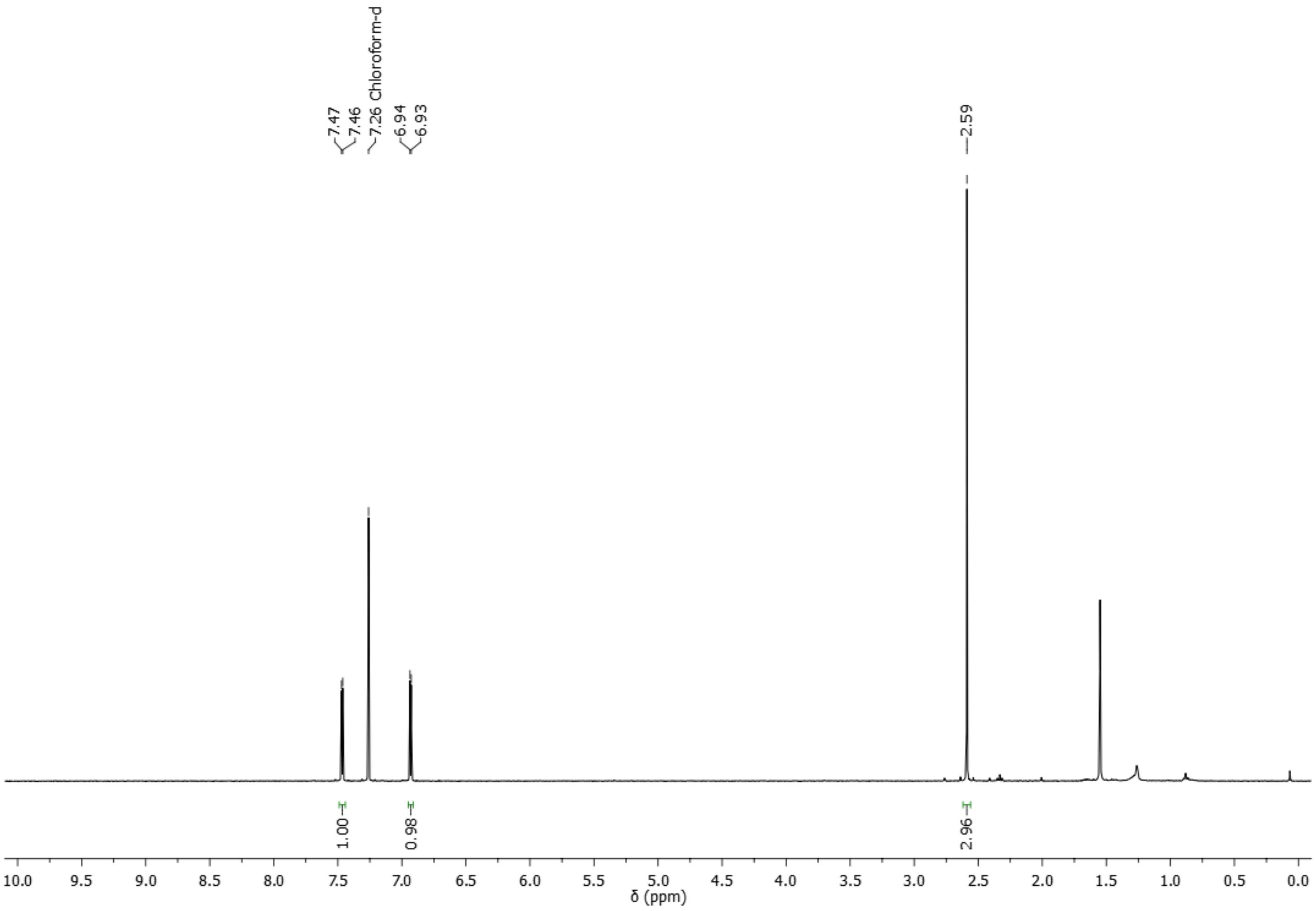

^{13}CNMR 100MHz, $CDCl_3$

3m

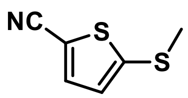

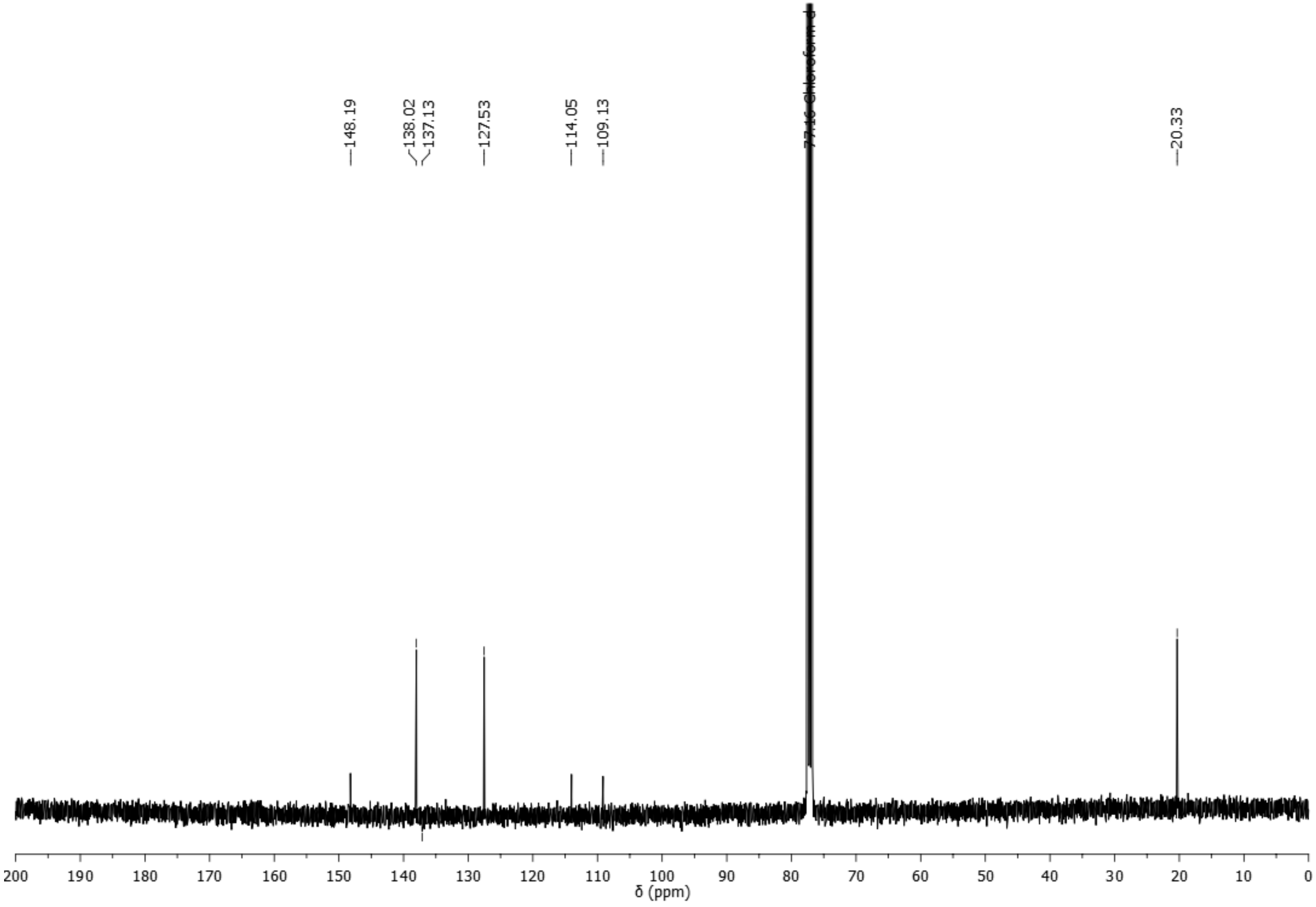

^{1}HNMR 400MHz, CD_2Cl_2

3n

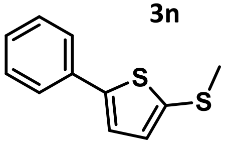

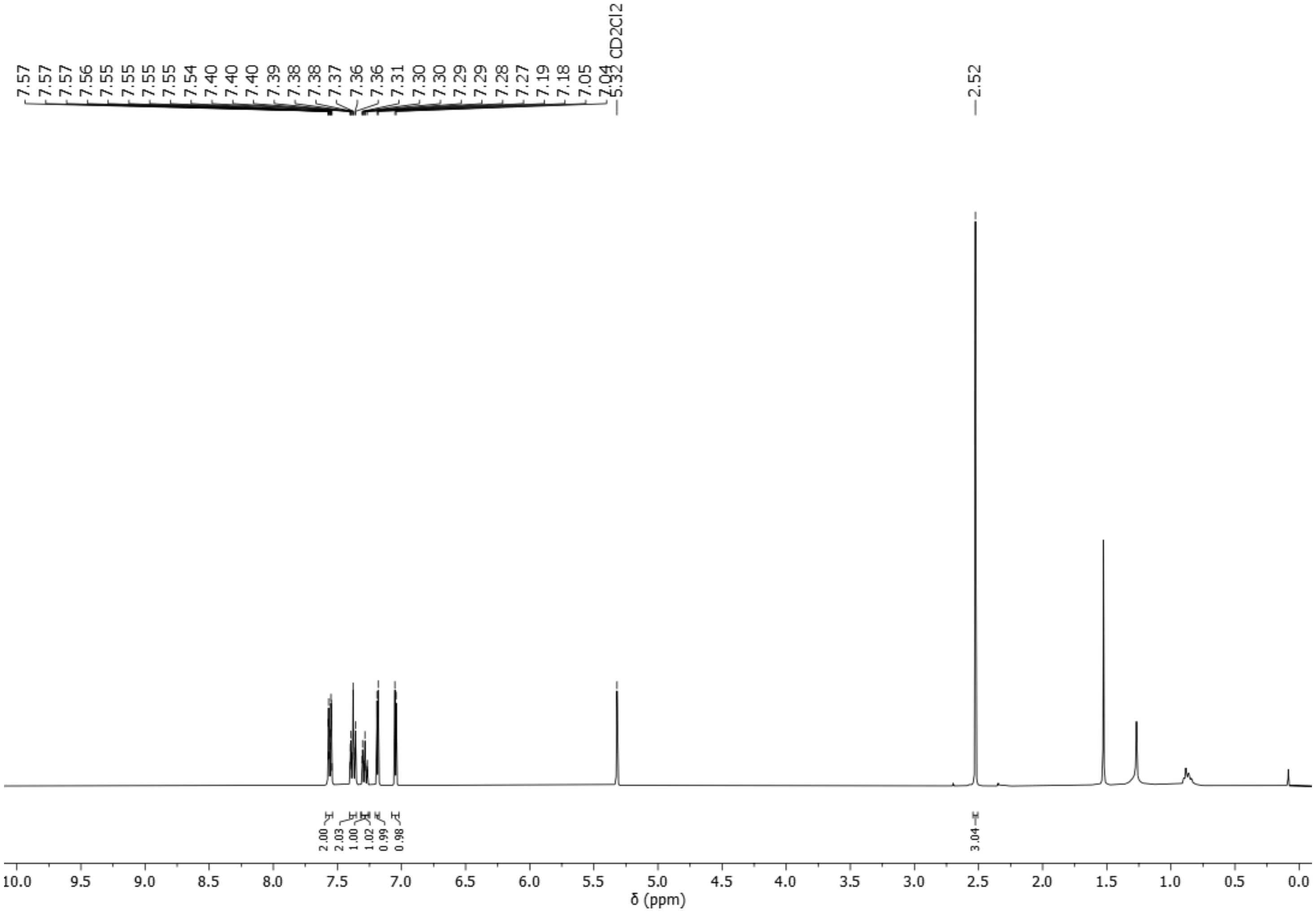

^{13}CNMR 100MHz, CD_2Cl_2

3n

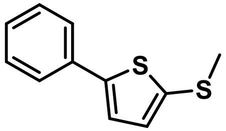

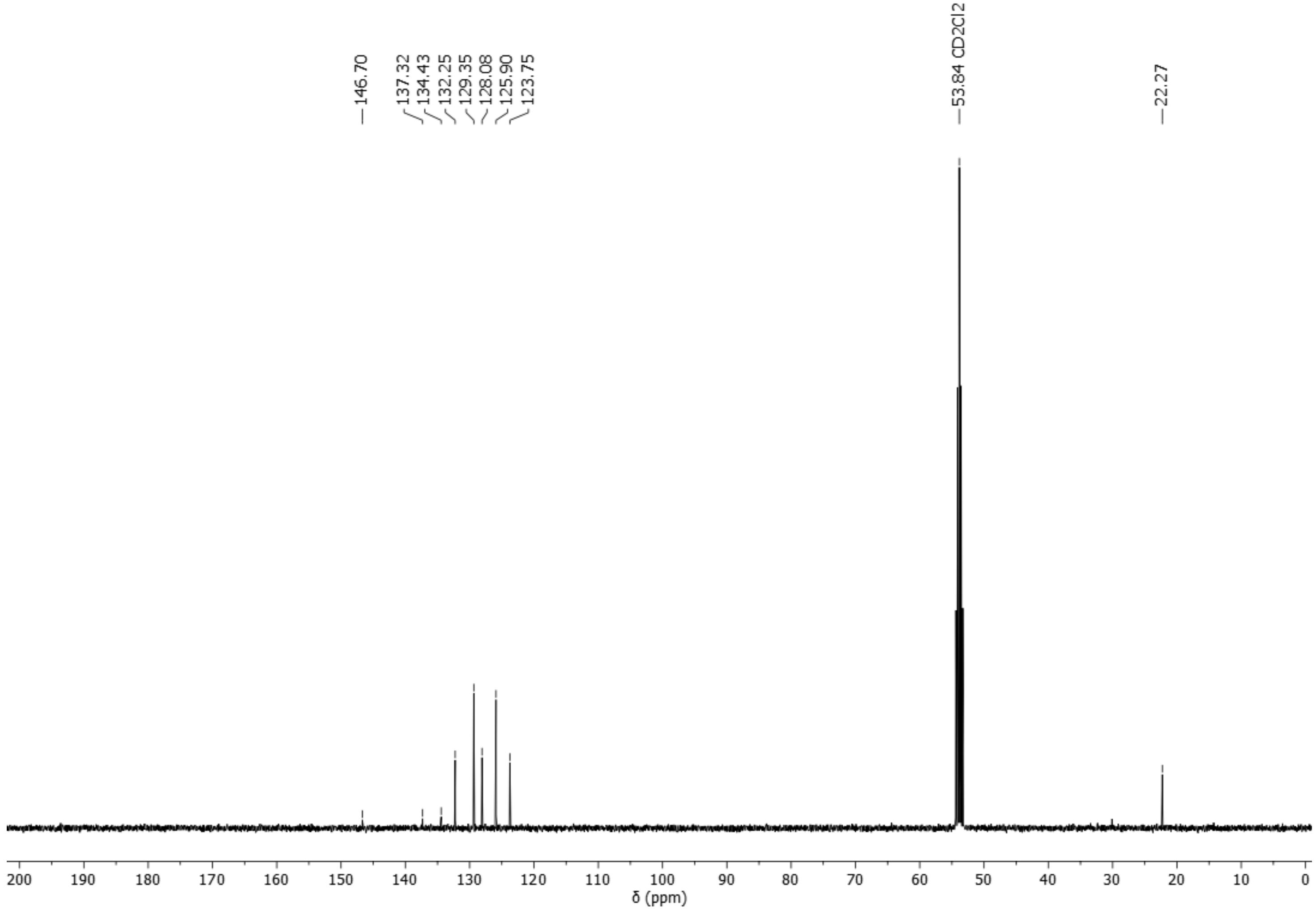

^{1}HNMR 400MHz, $CDCl_3$

3o

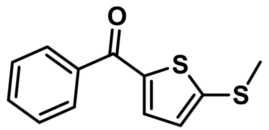

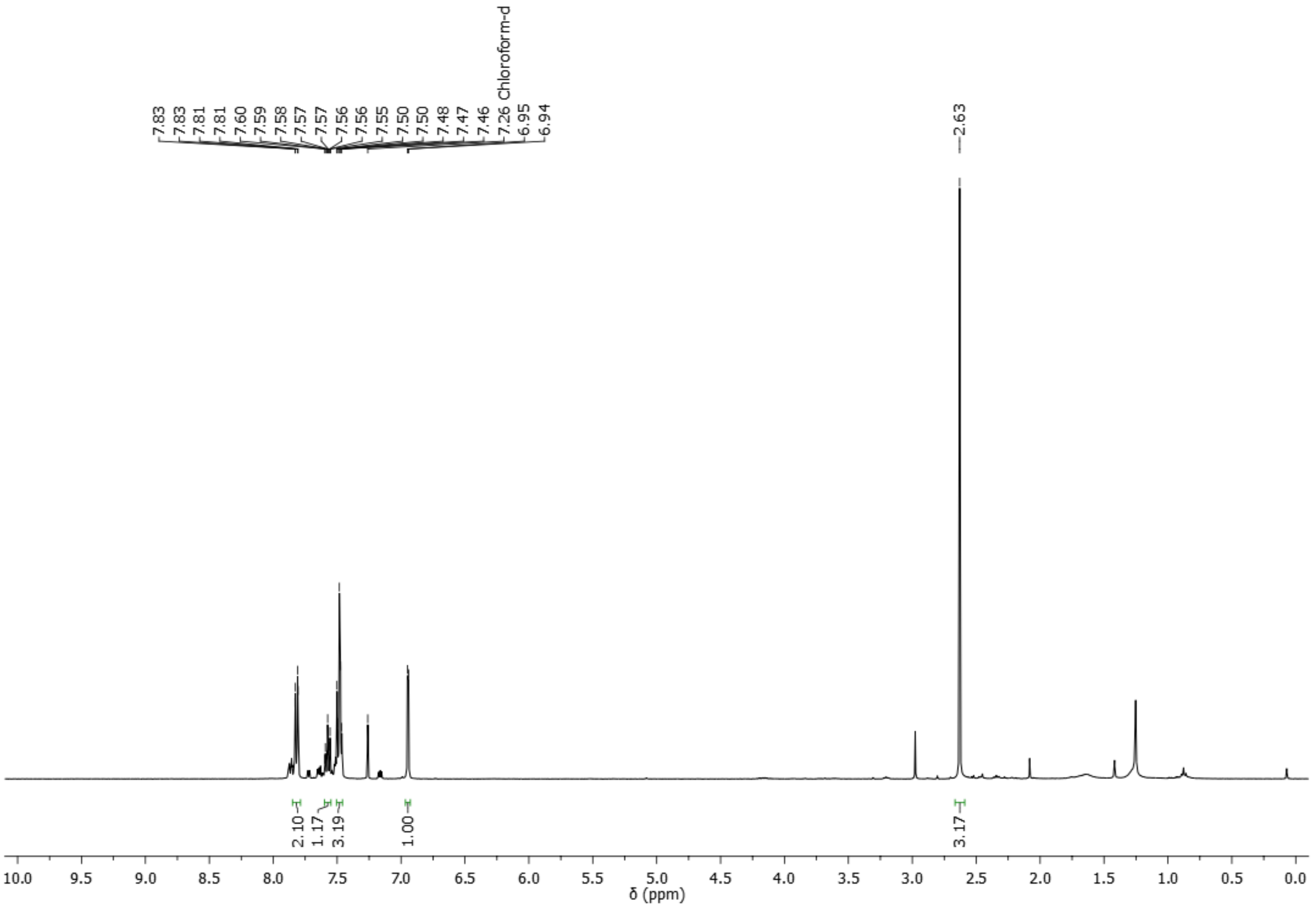

^{13}CNMR 100MHz, $CDCl_3$

3o

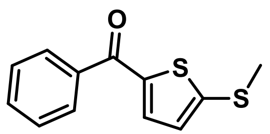

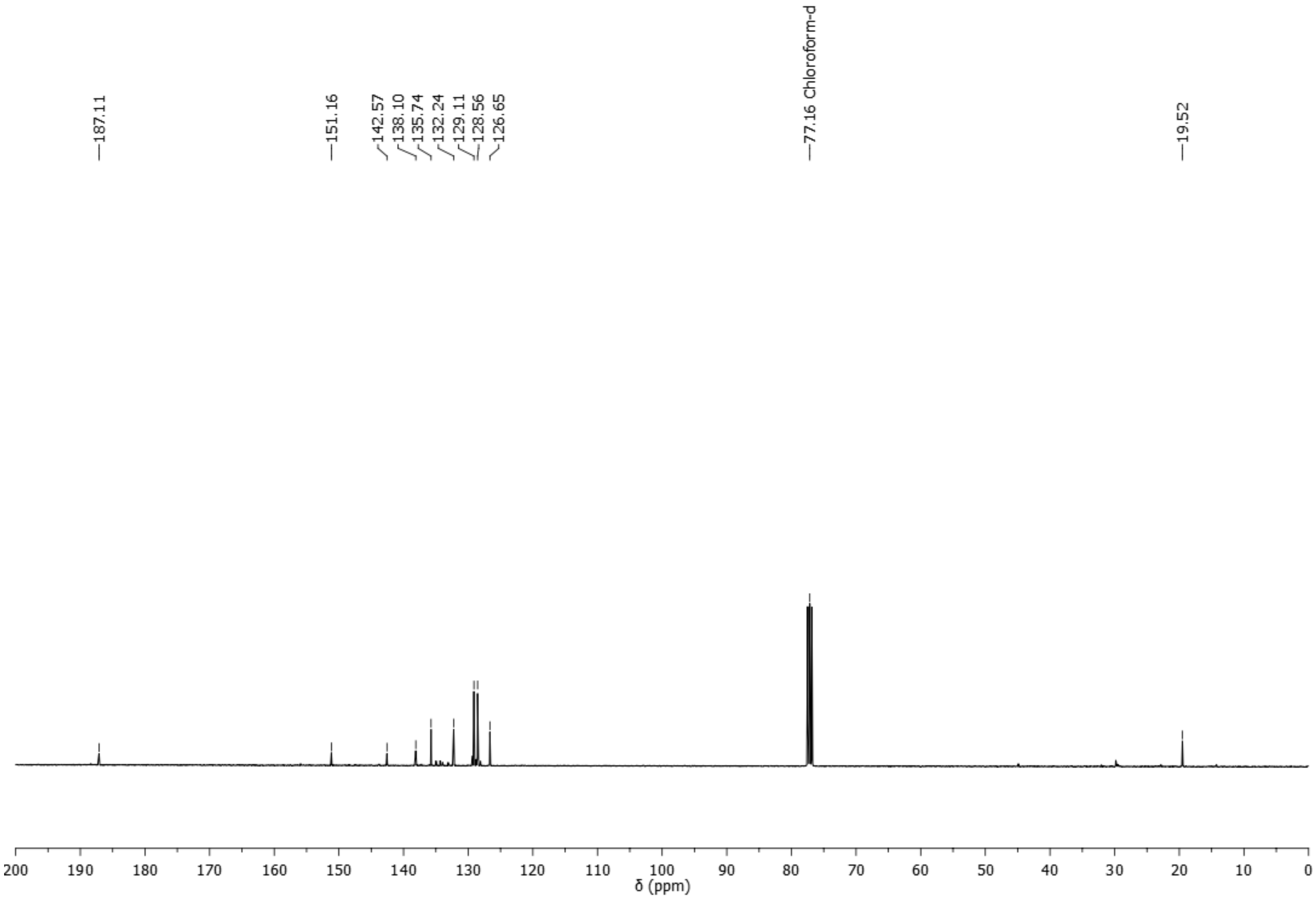

^{1}HNMR 400MHz, $CDCl_3$

3p

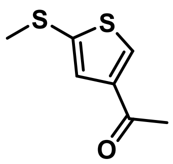

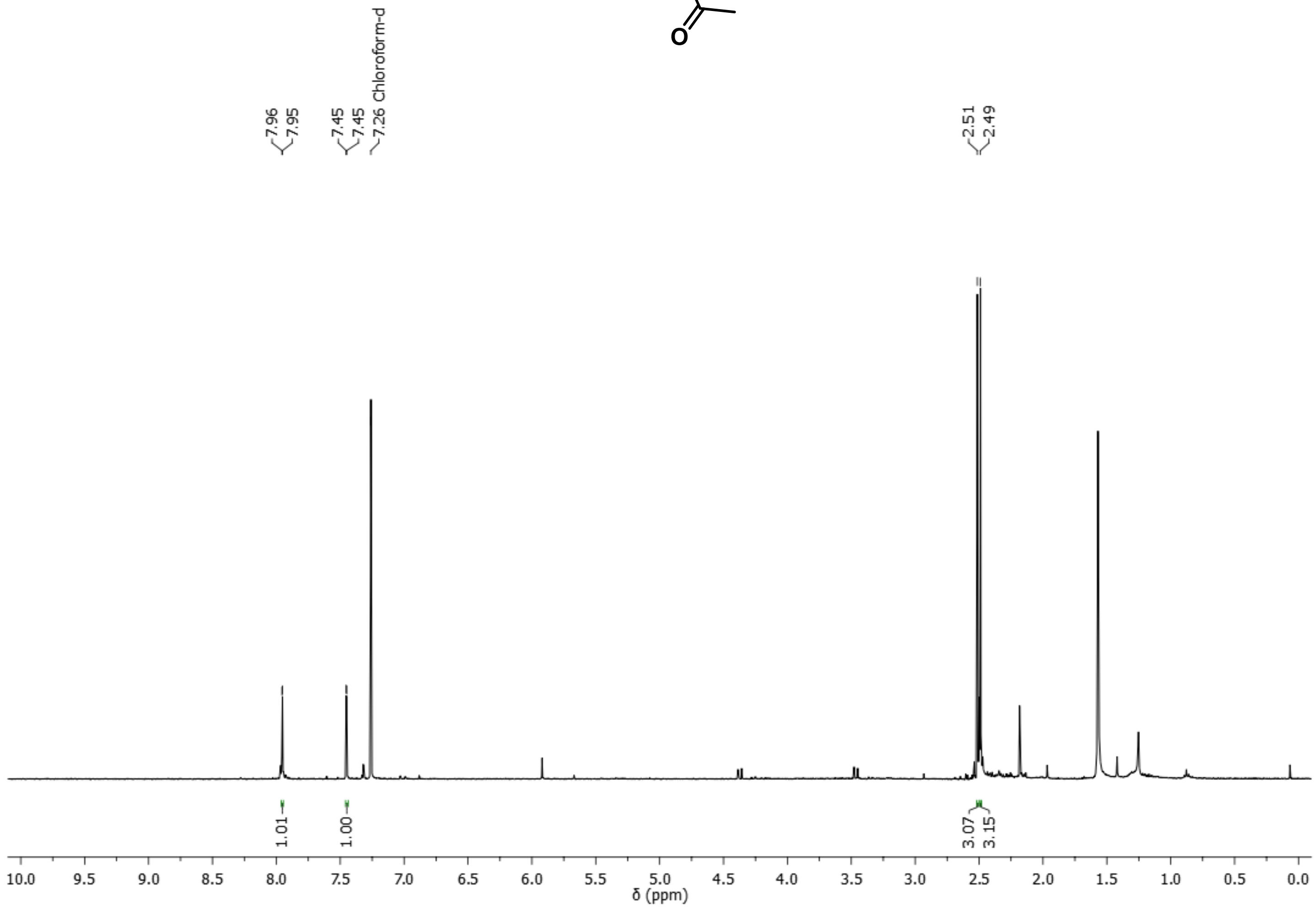

^{13}CNMR 100MHz, $CDCl_3$

3p

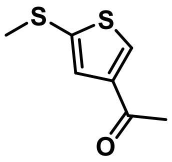

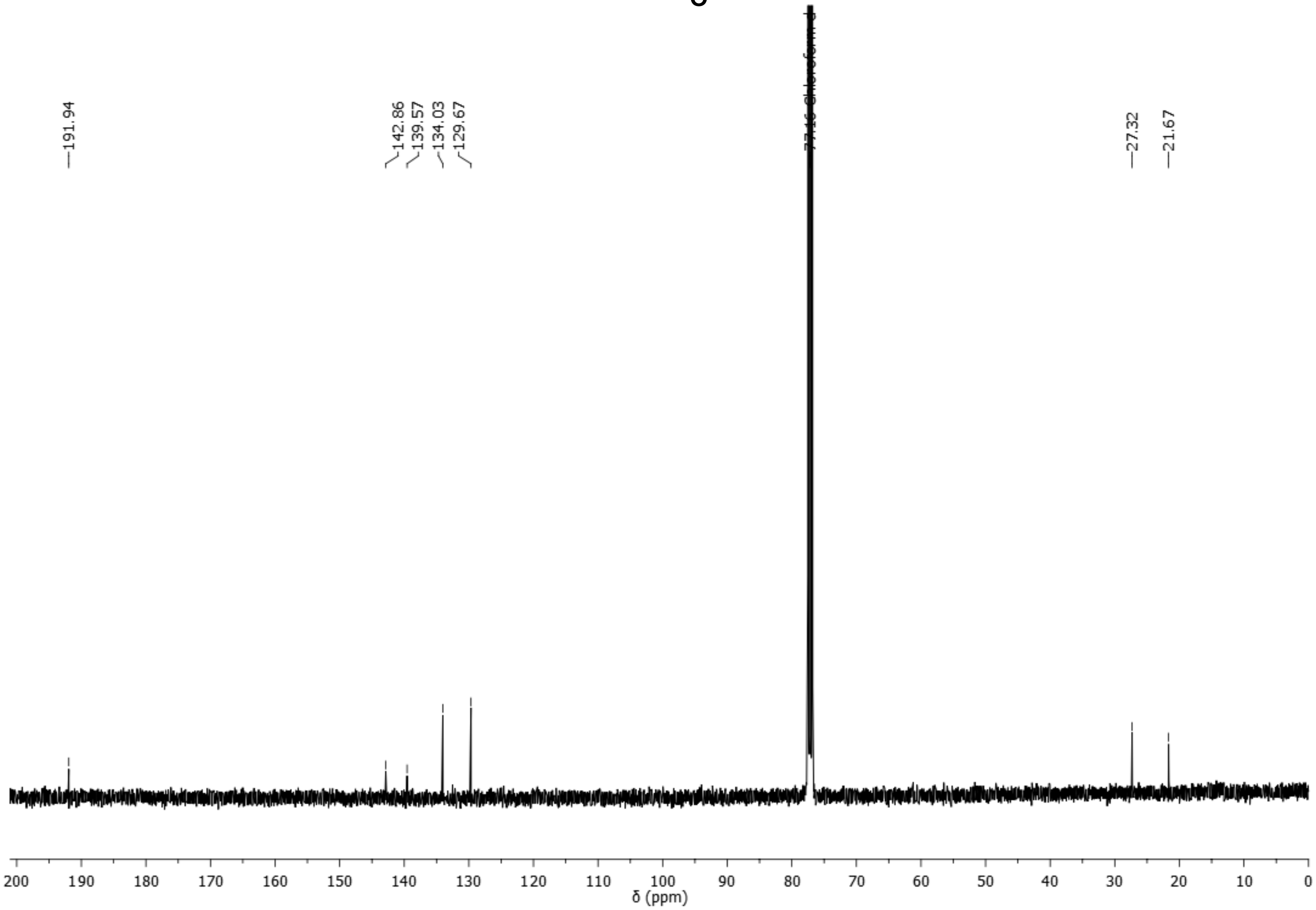

^{1}HNMR 400MHz, $CDCl_3$

3q

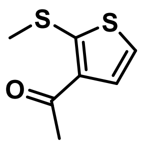

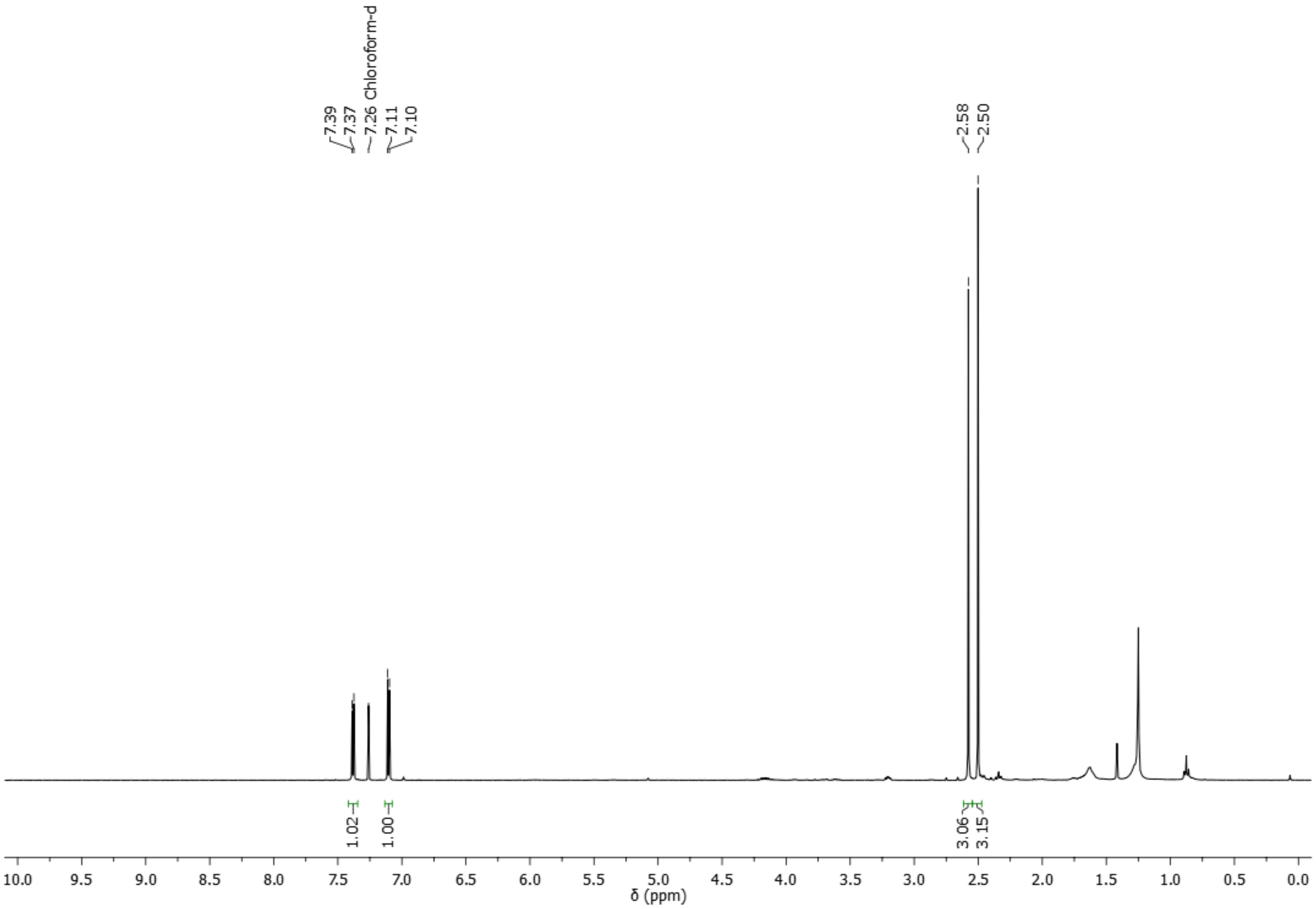

^{13}CNMR 100MHz, $CDCl_3$

3q

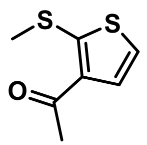

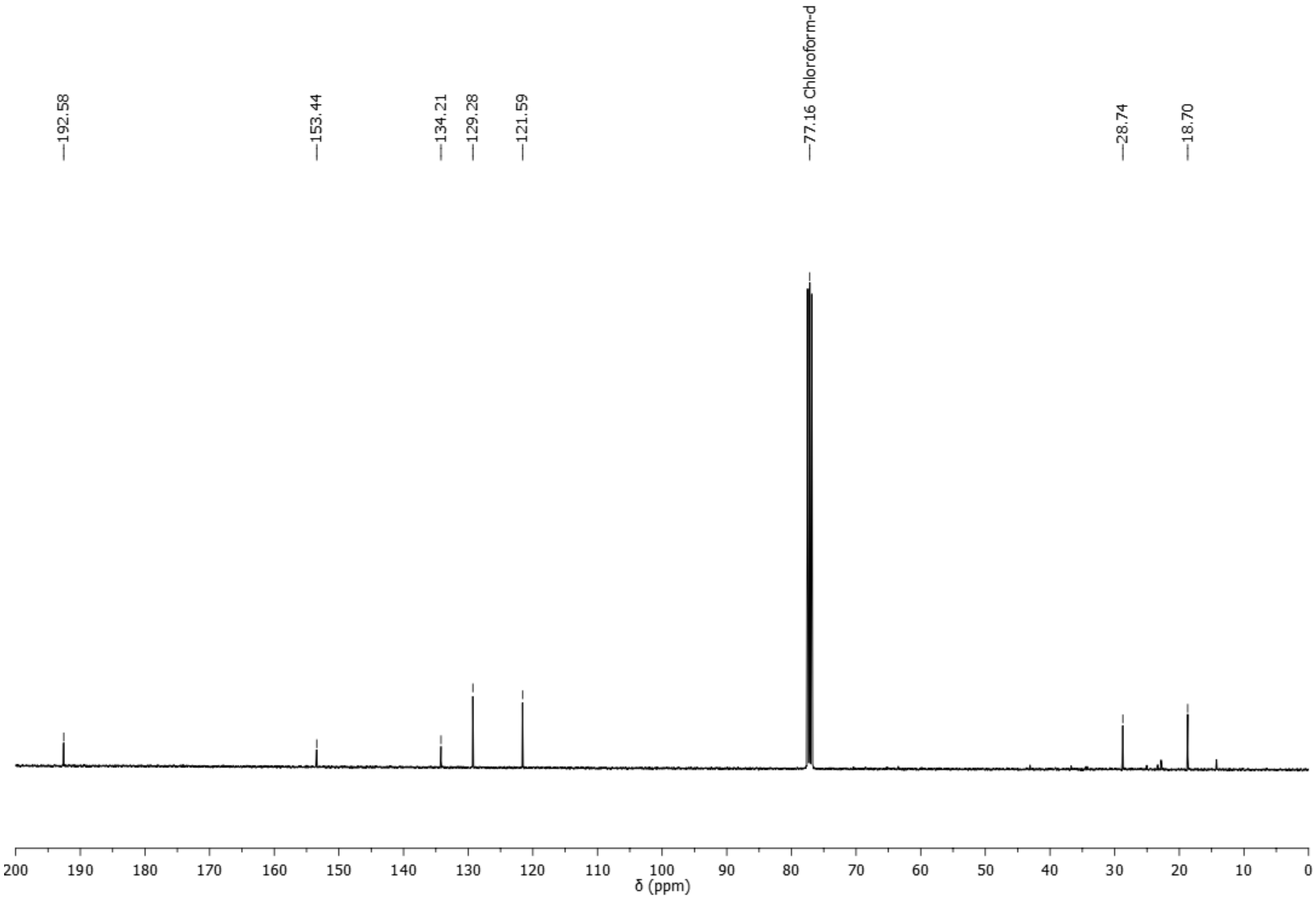

^{1}HNMR 400MHz, $CDCl_3$

3r

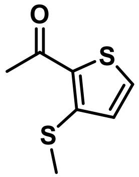

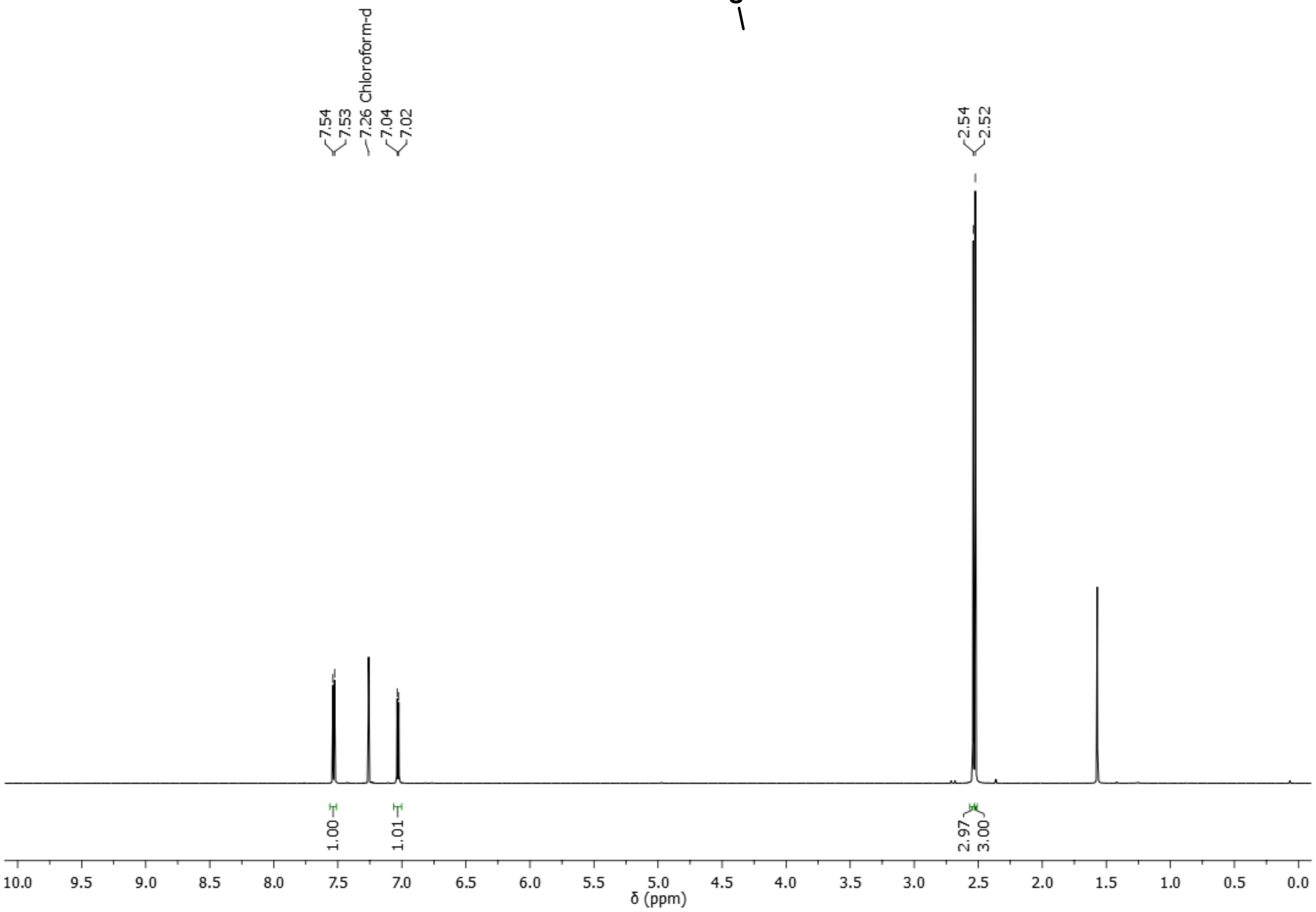

^{13}CNMR 100MHz, $CDCl_3$

3r

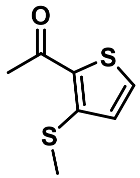

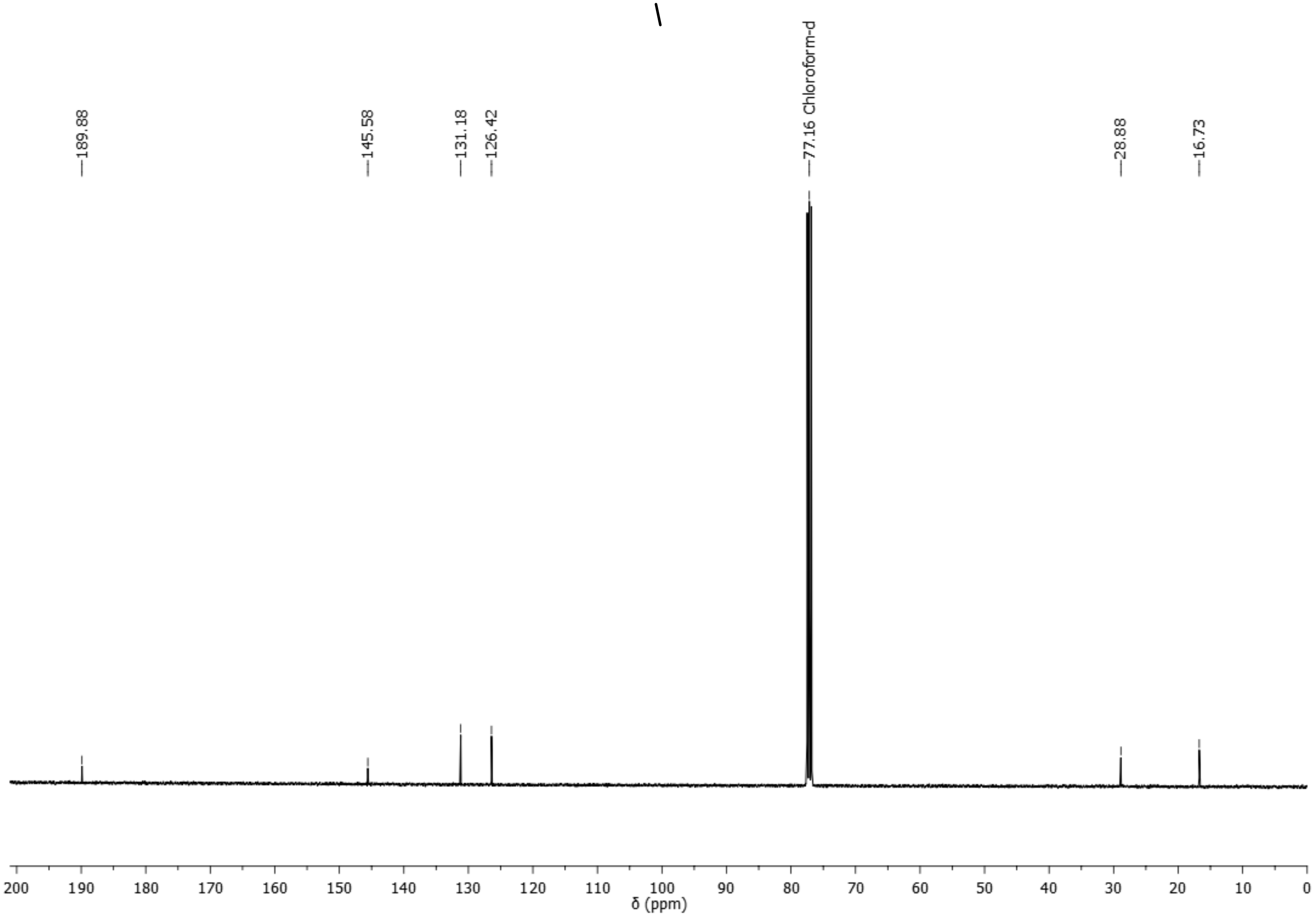

^{1}HNMR 400MHz, $CDCl_3$

3s

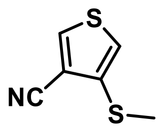

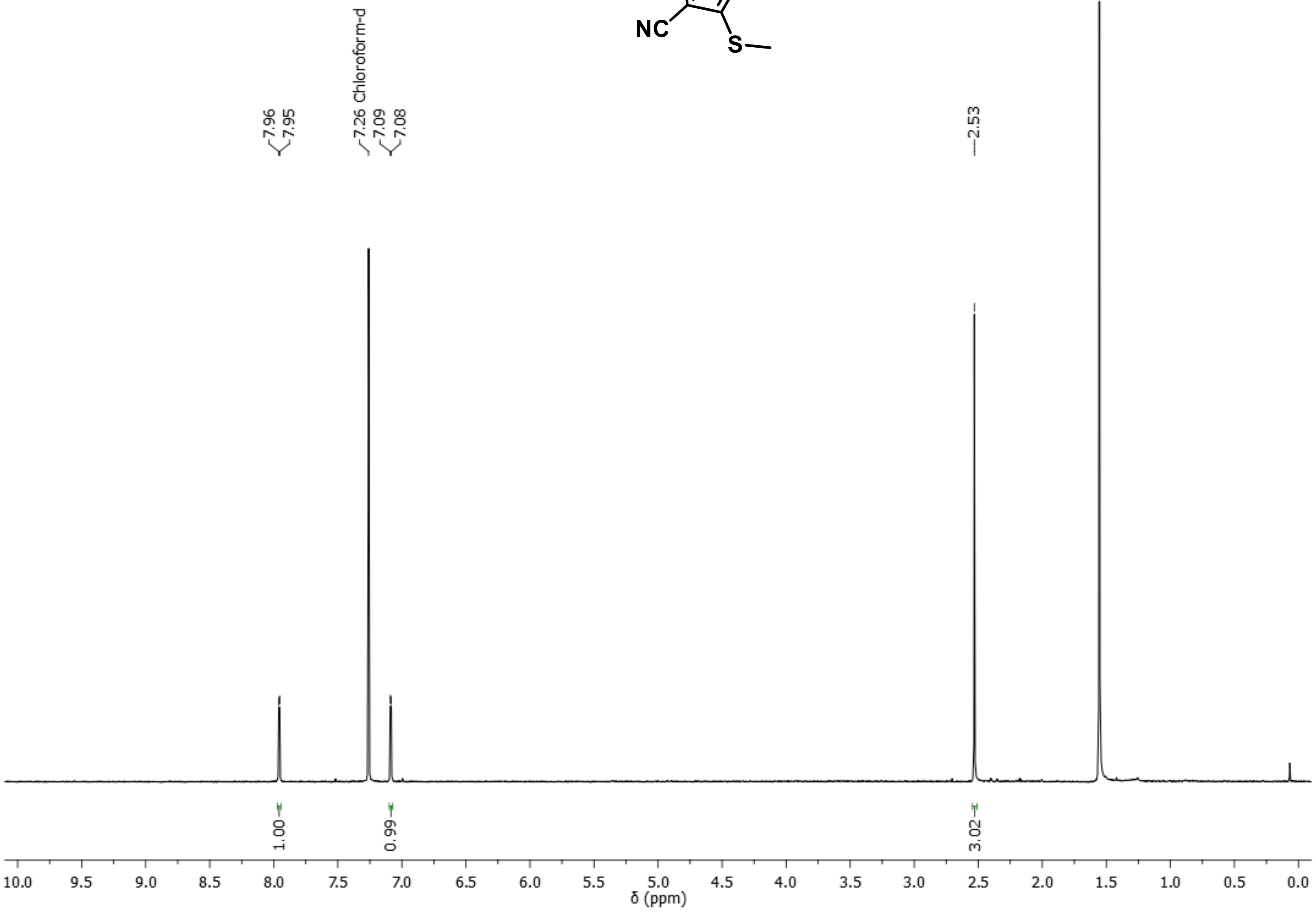

^{13}CNMR 100MHz, CDCl$_3$

3s

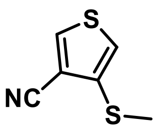

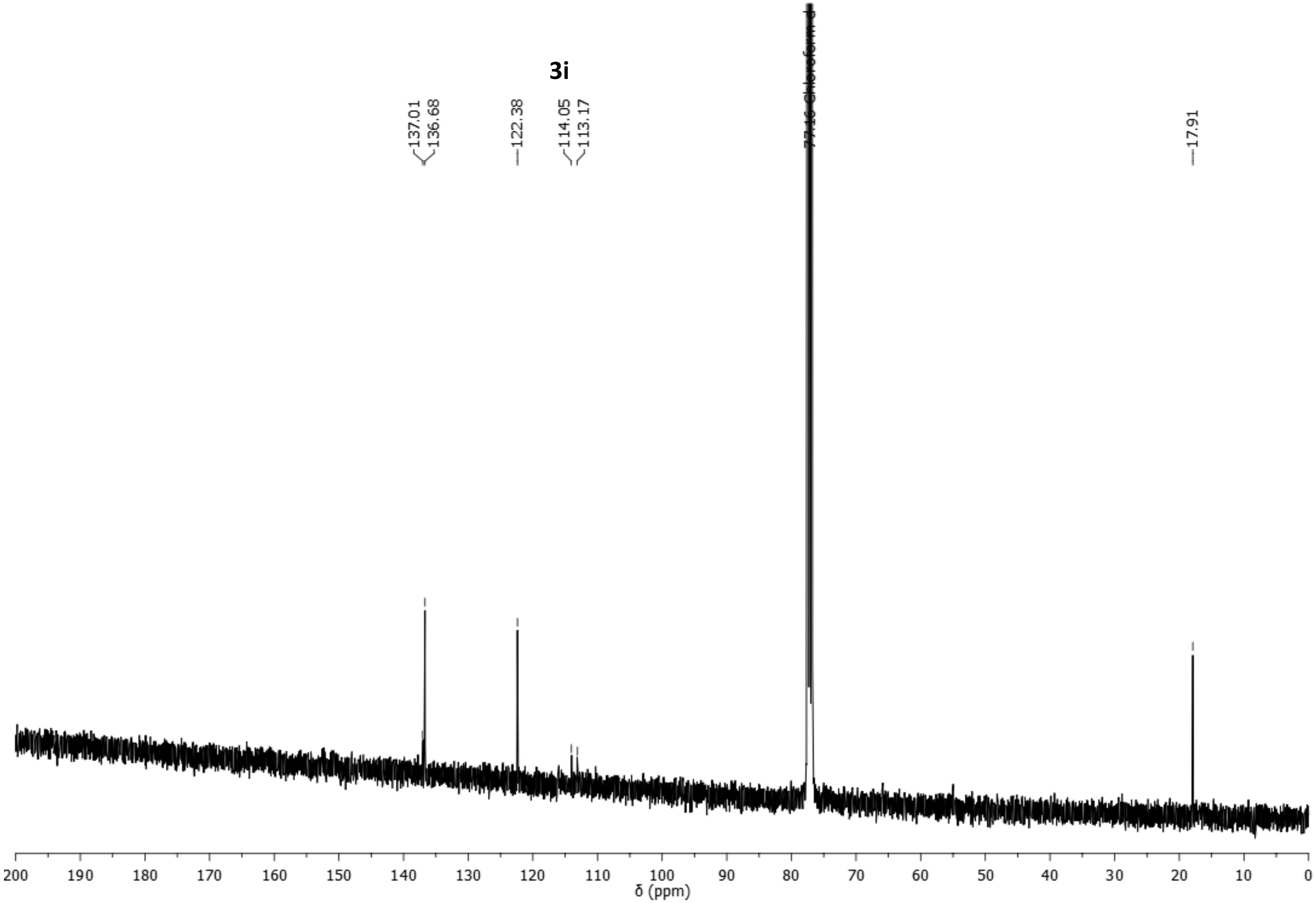

^{1}HNMR 400MHz, $CDCl_3$

3t

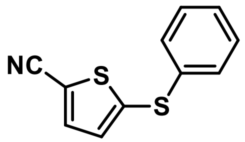

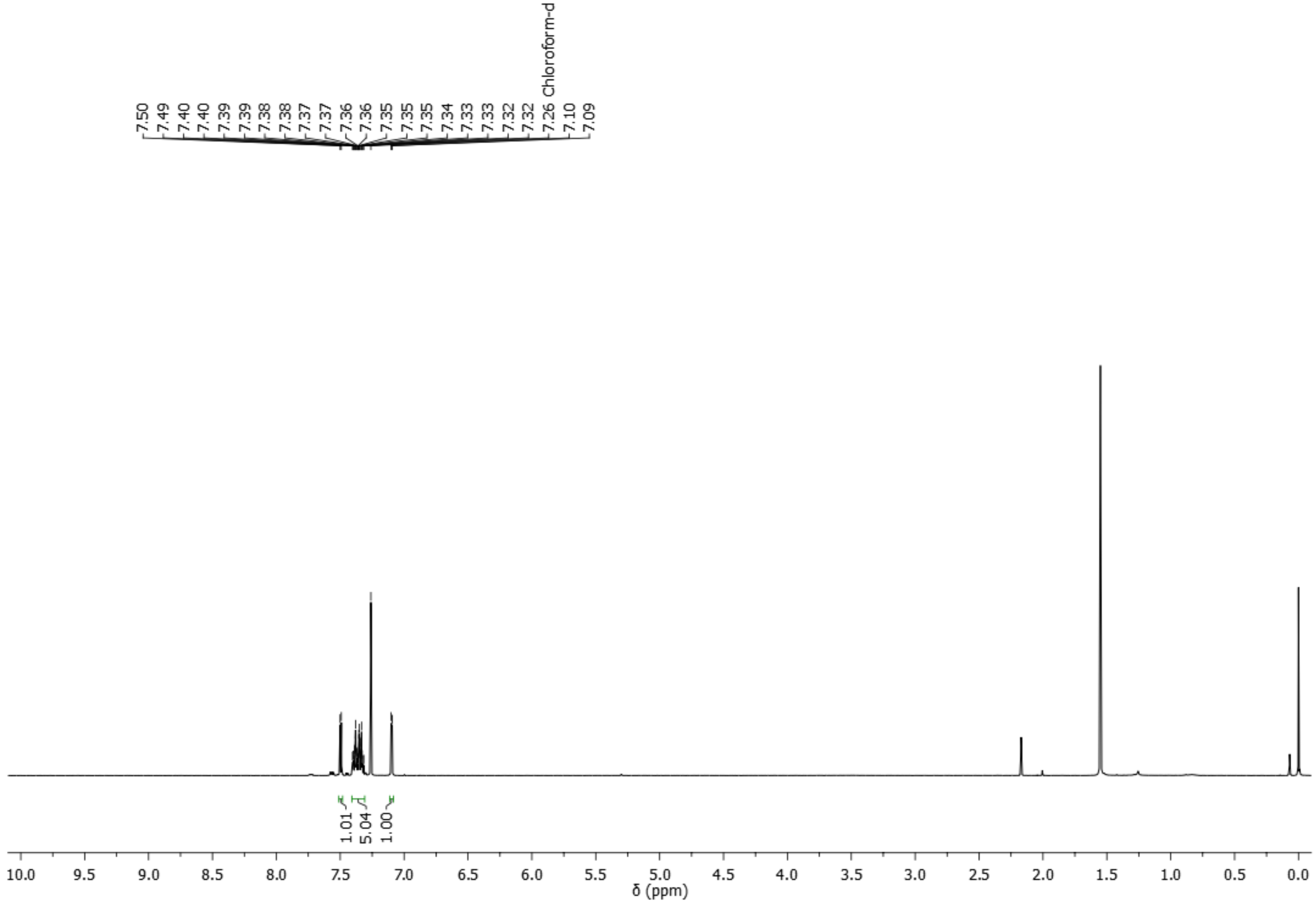

^{13}CNMR 100MHz, $CDCl_3$

3t

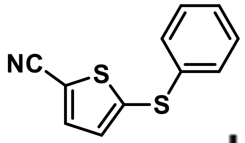

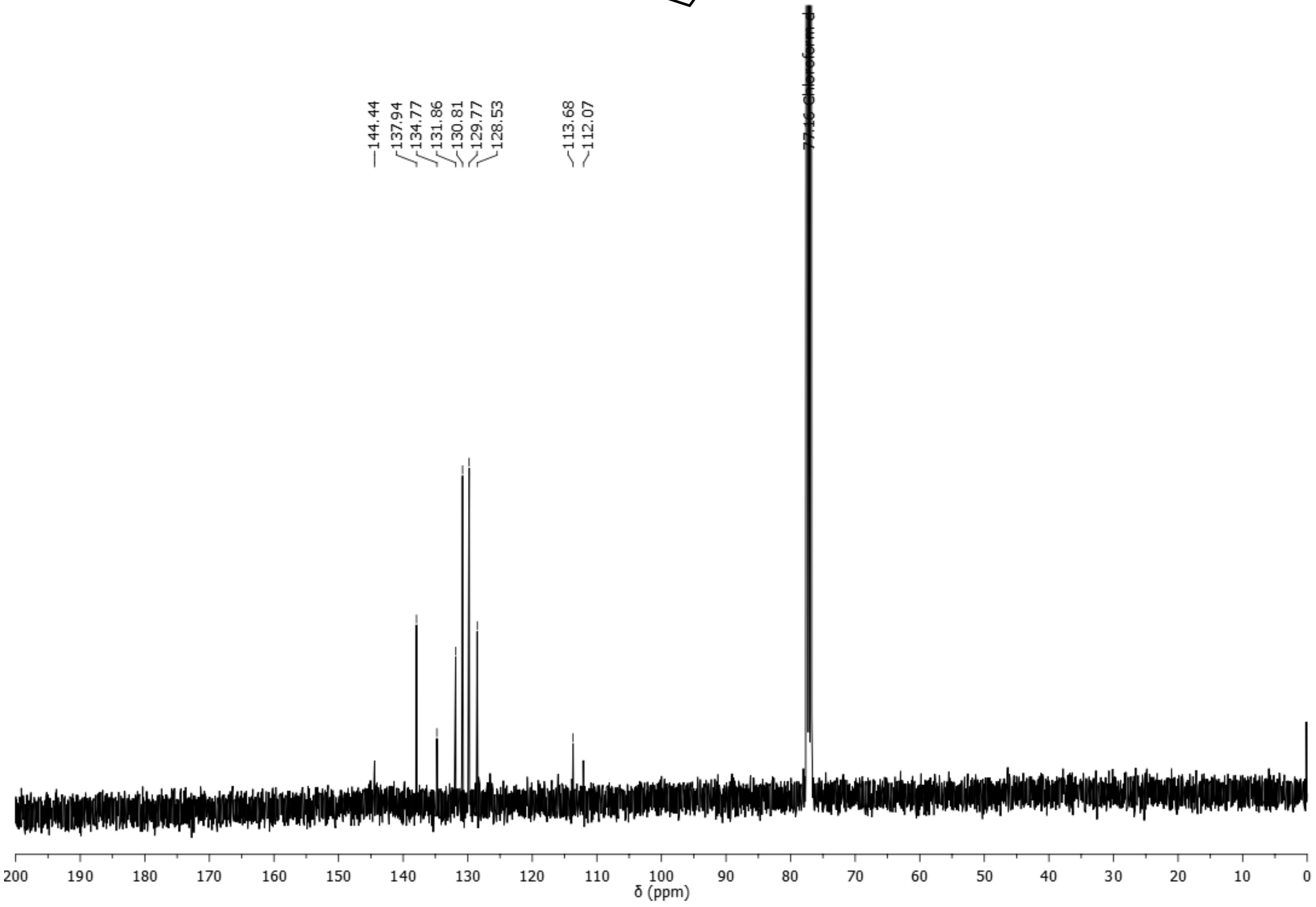

^{1}HNMR 400MHz, $CDCl_3$

3u

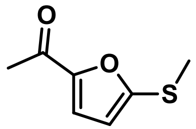

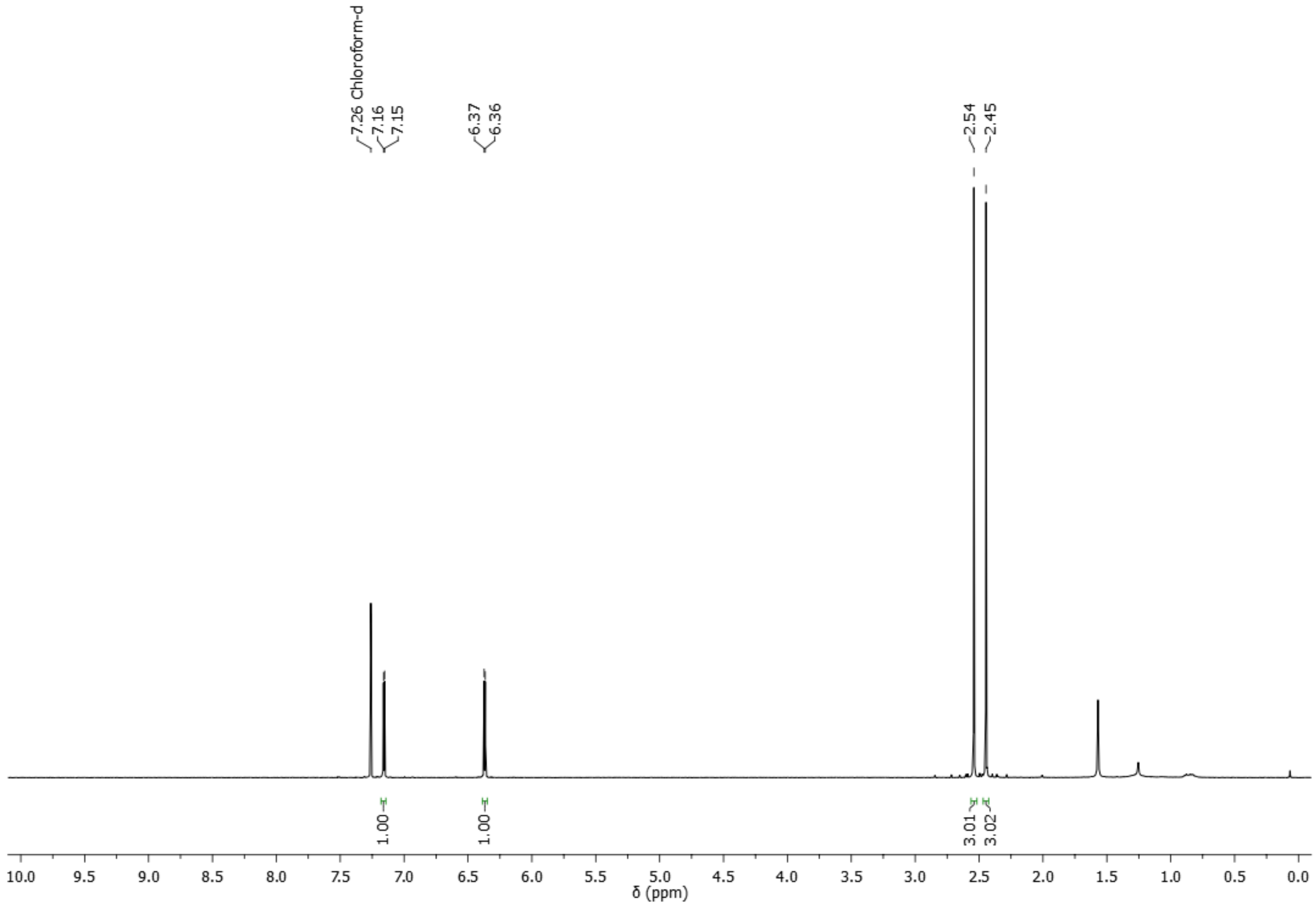

^{13}CNMR 100MHz, $CDCl_3$

3u

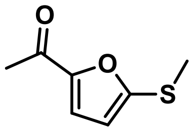

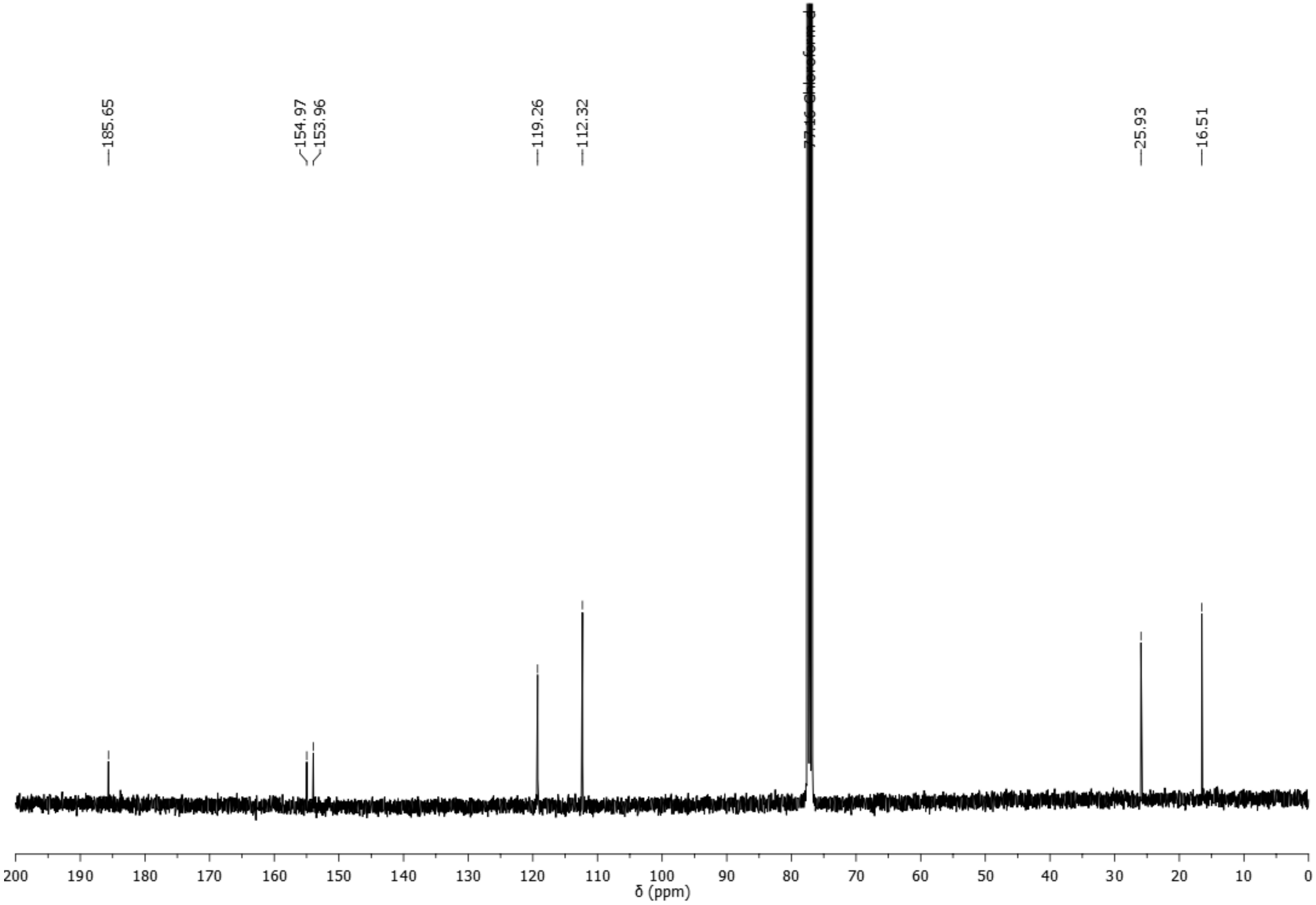

^{1}HNMR 400MHz, $CDCl_3$

3v

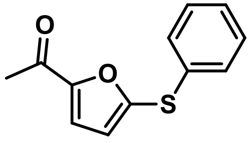

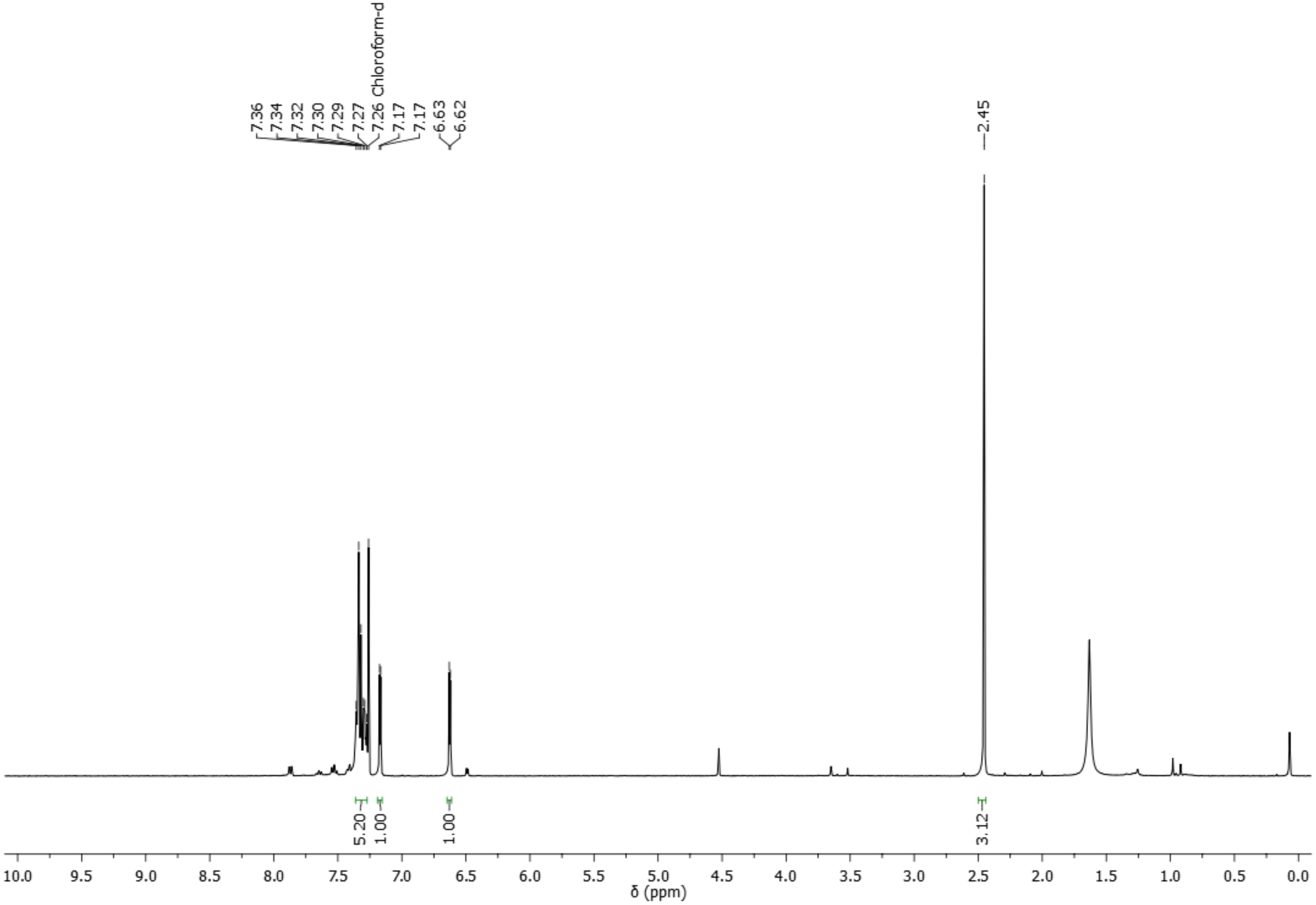

^{13}CNMR 100MHz, $CDCl_3$

3v

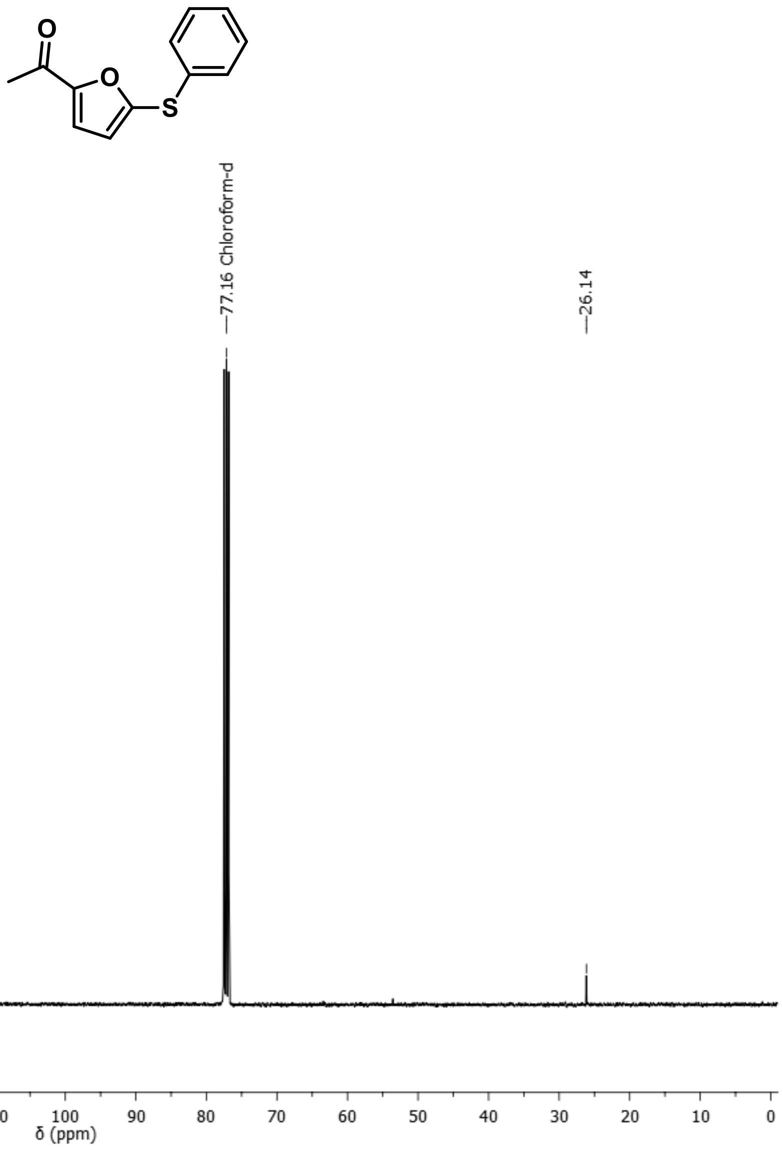

^{1}HNMR 400MHz, $CDCl_3$

3w

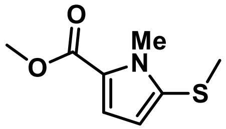

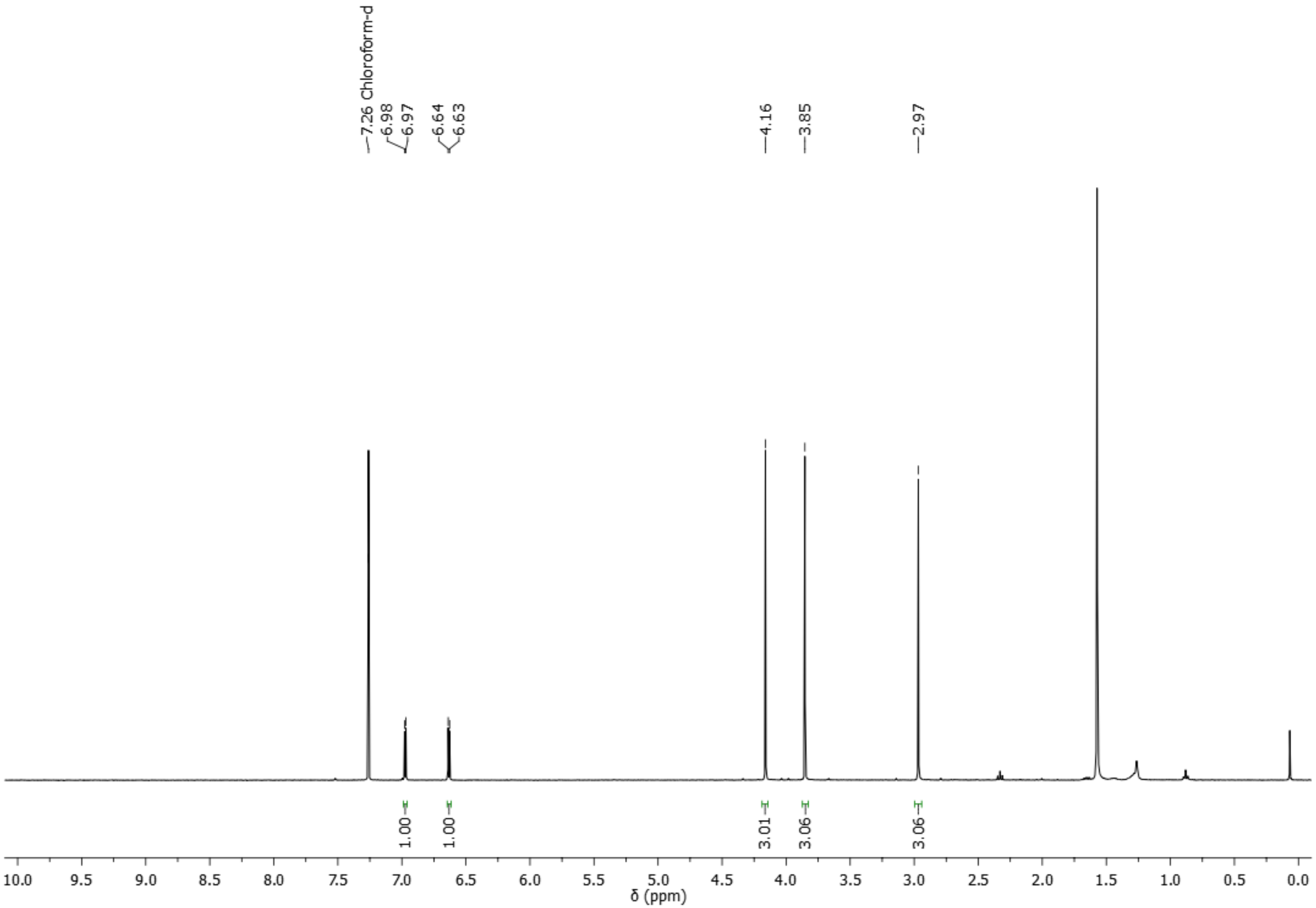

^{13}CNMR 100MHz, $CDCl_3$

3w

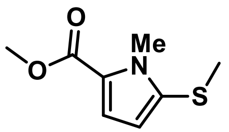

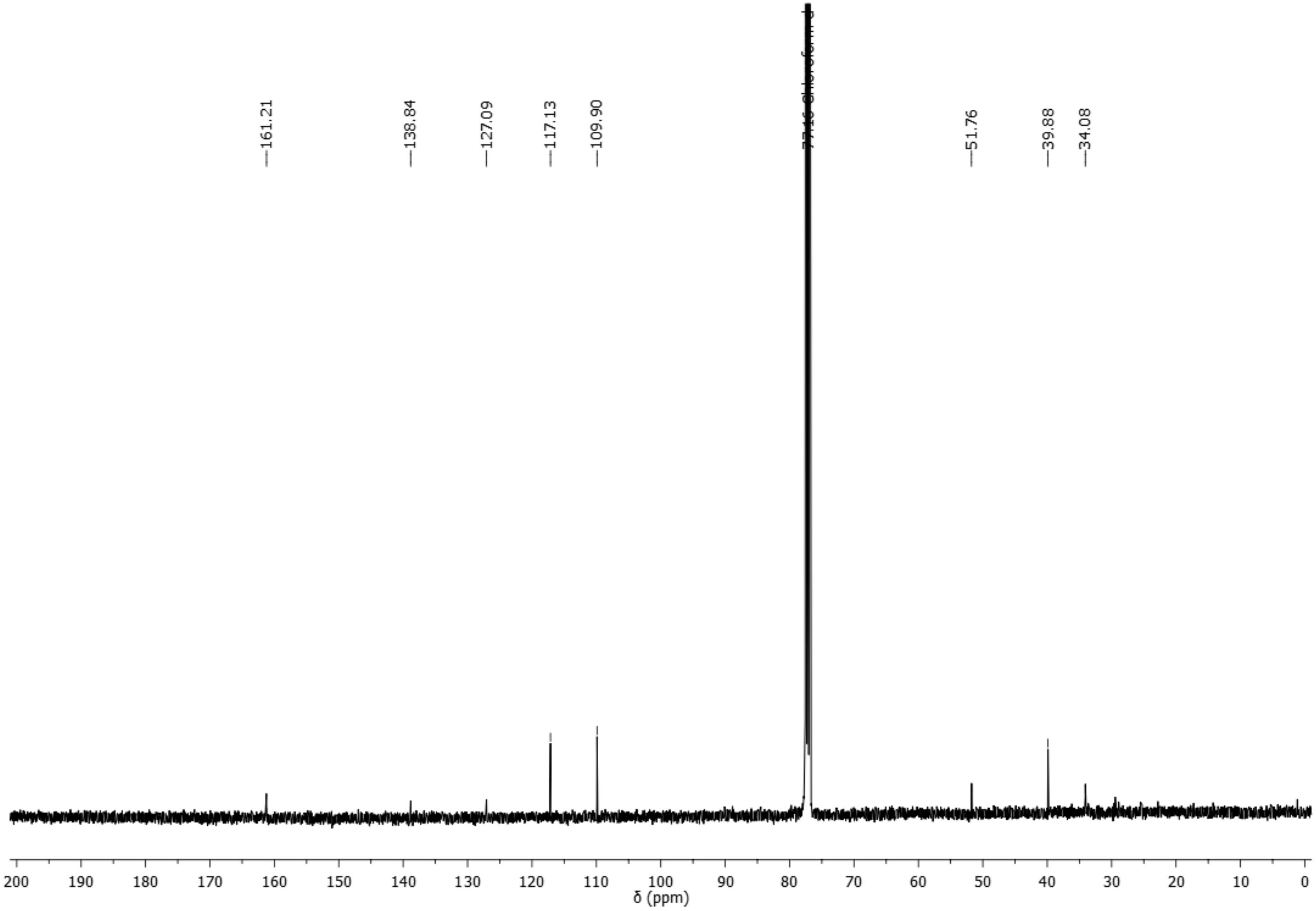

^{1}HNMR 400MHz, $CDCl_3$

3x

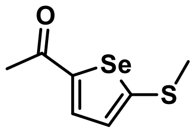

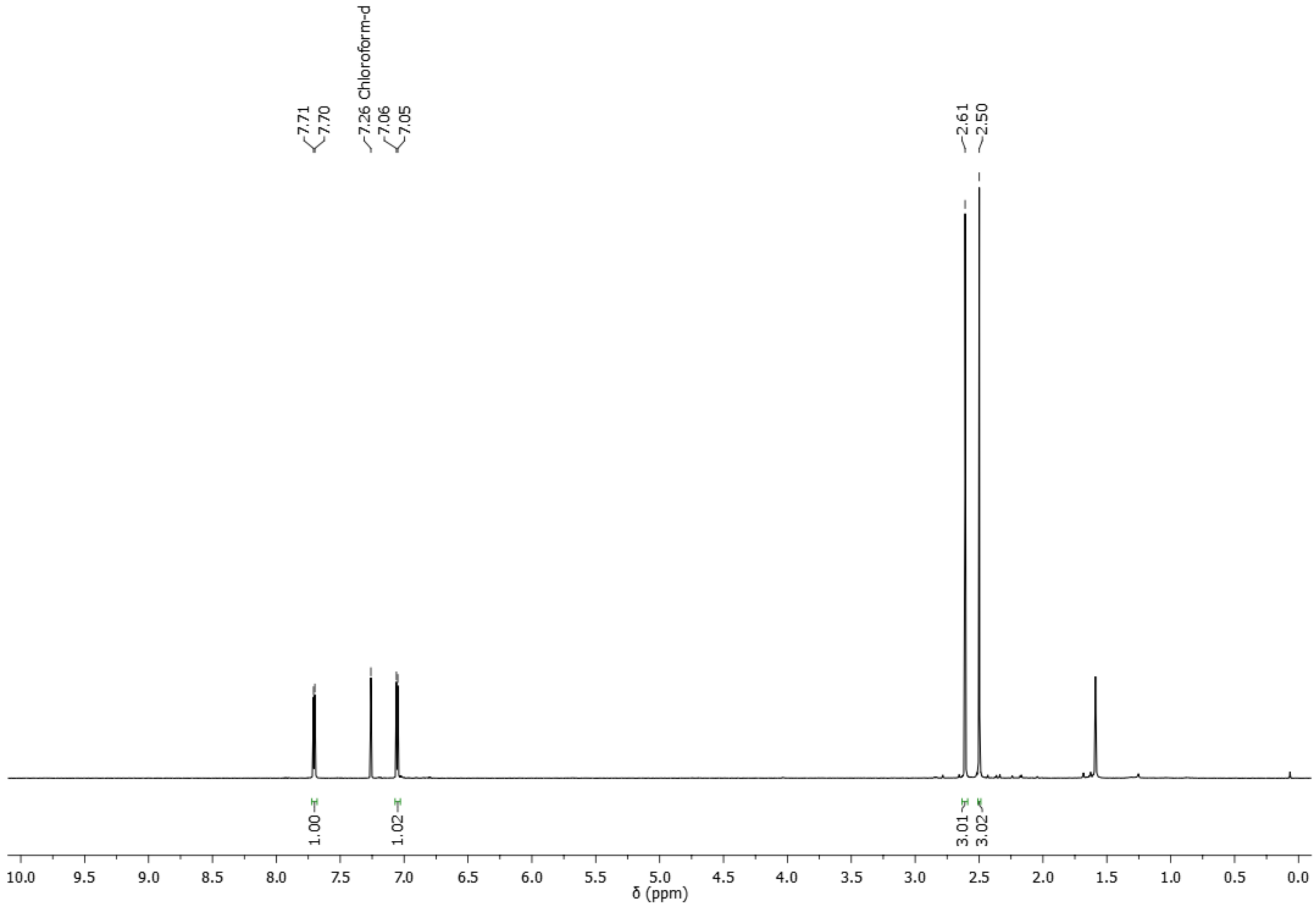

^{13}CNMR 100MHz, $CDCl_3$

3x

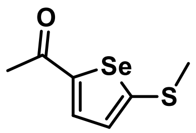

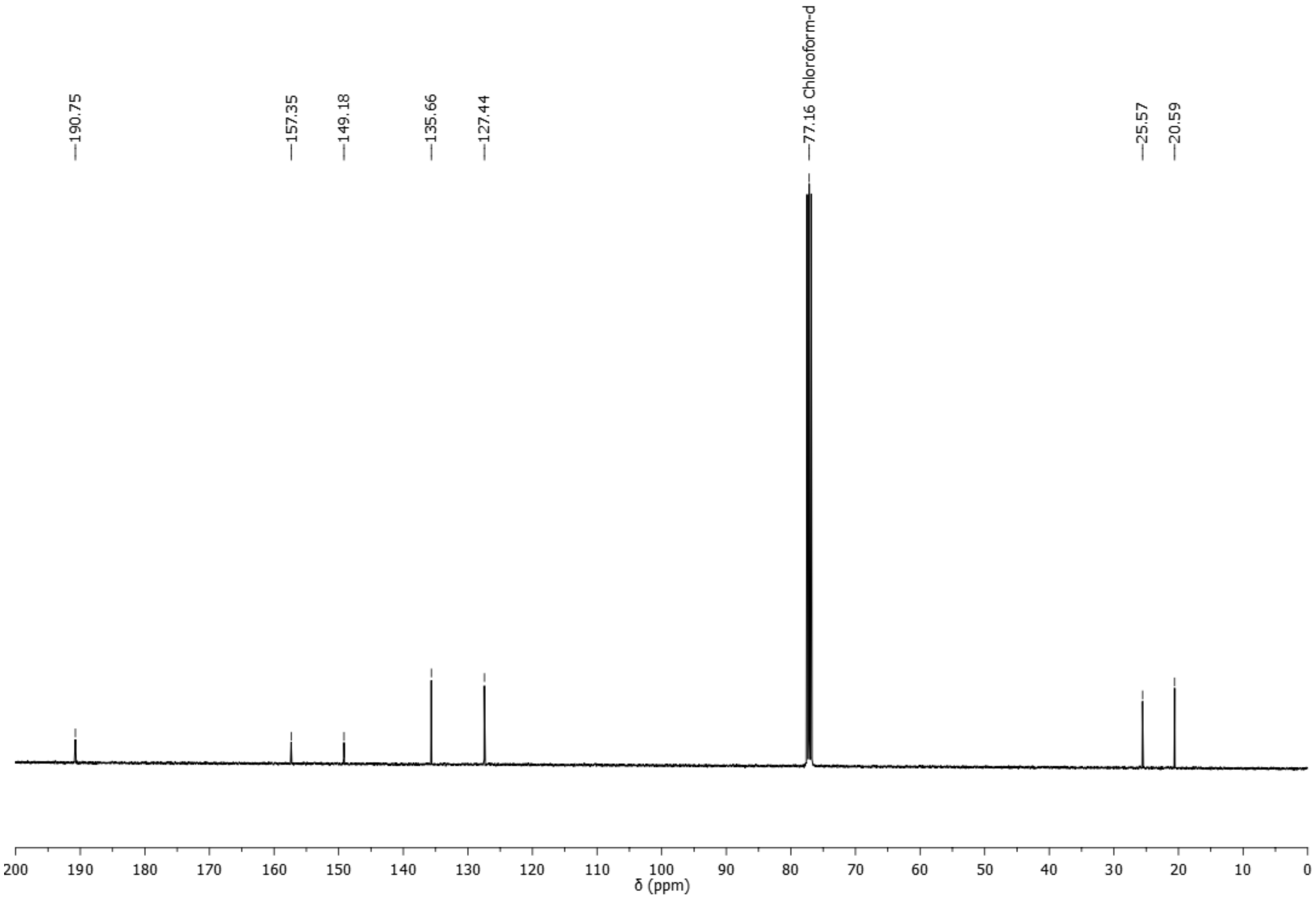

^{1}HNMR 400MHz, $CDCl_3$

3y

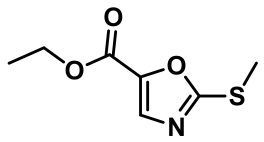

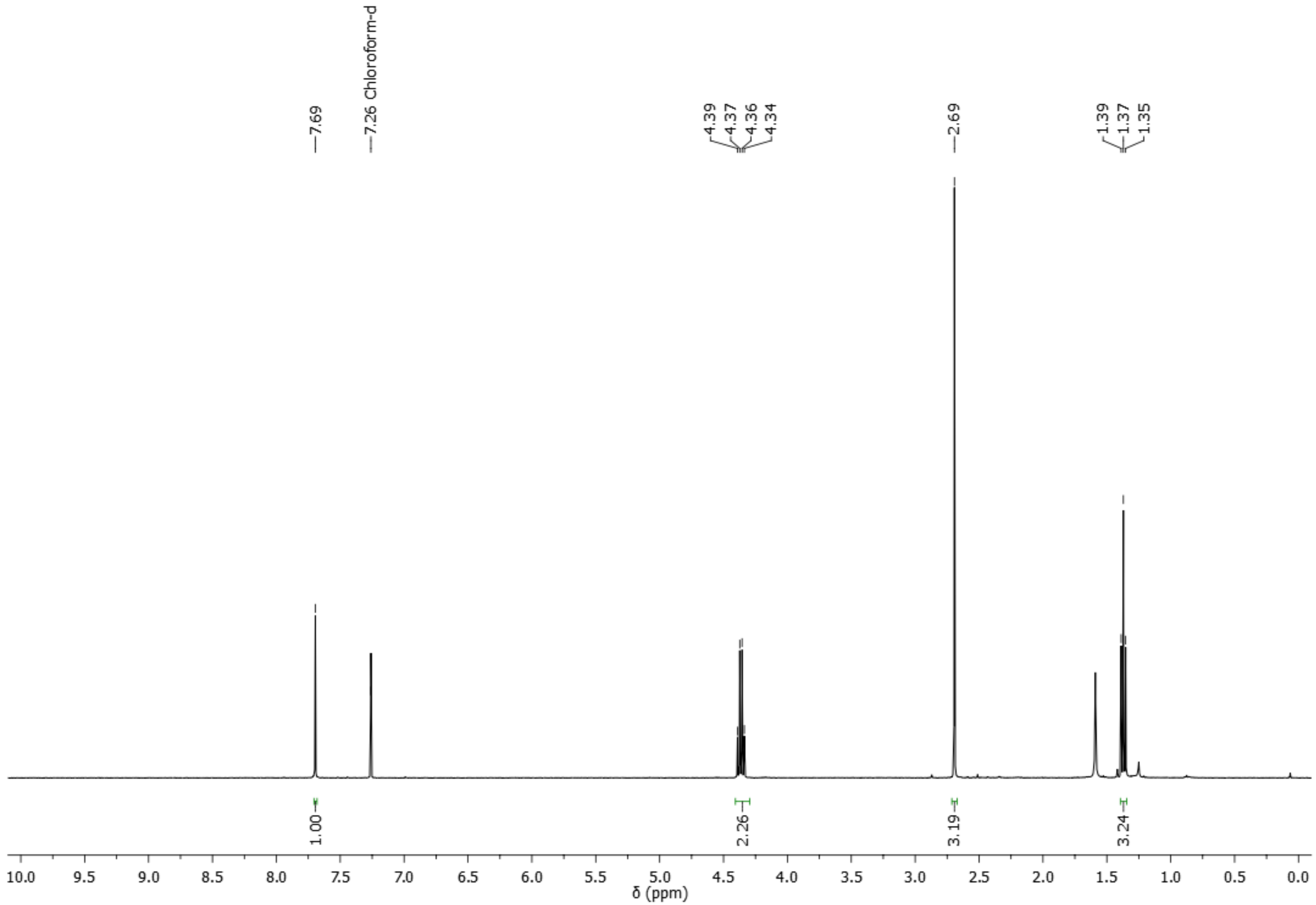

^{13}CNMR 100MHz, $CDCl_3$

3y

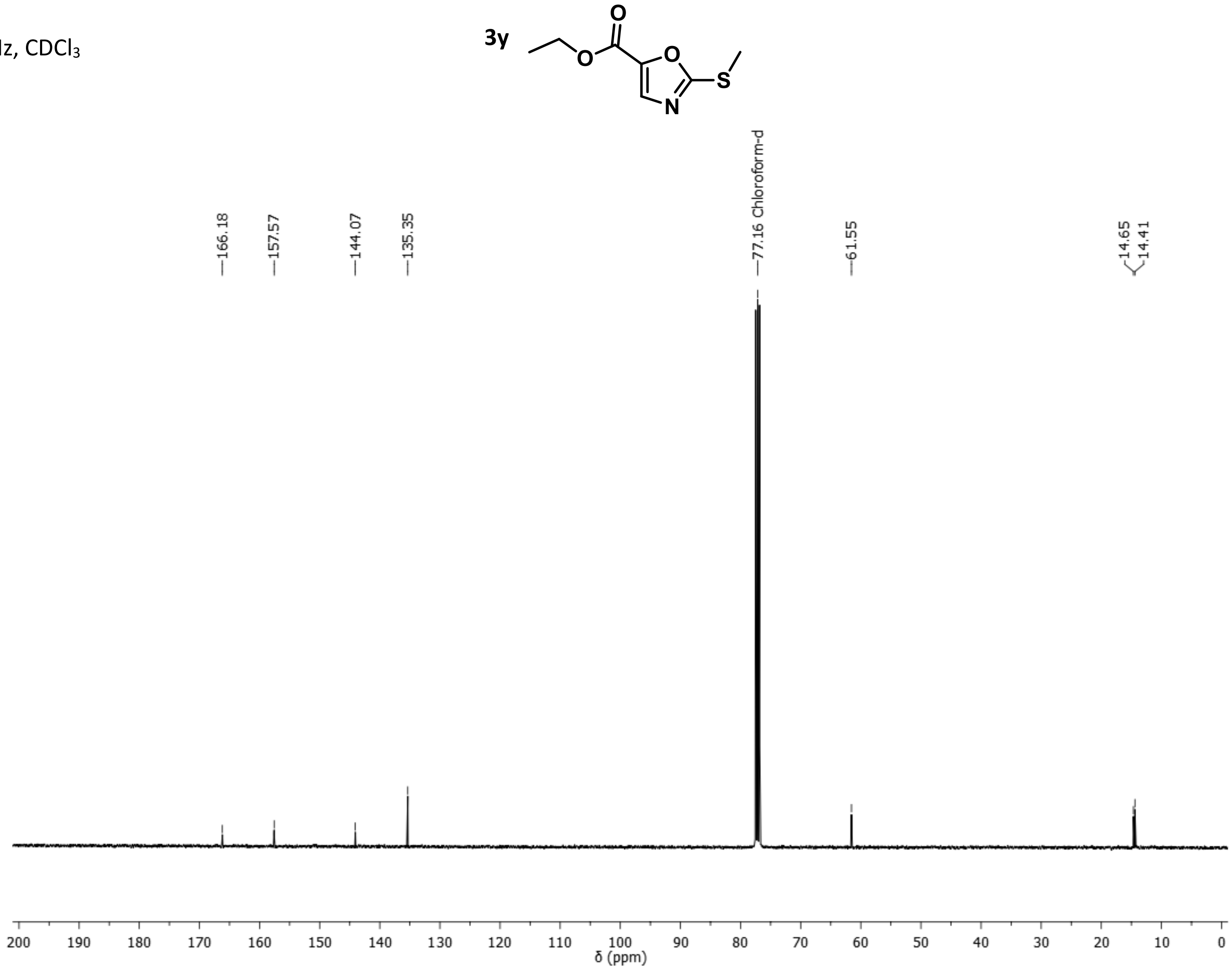

^{1}HNMR 400MHz, $CDCl_3$

3z

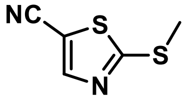

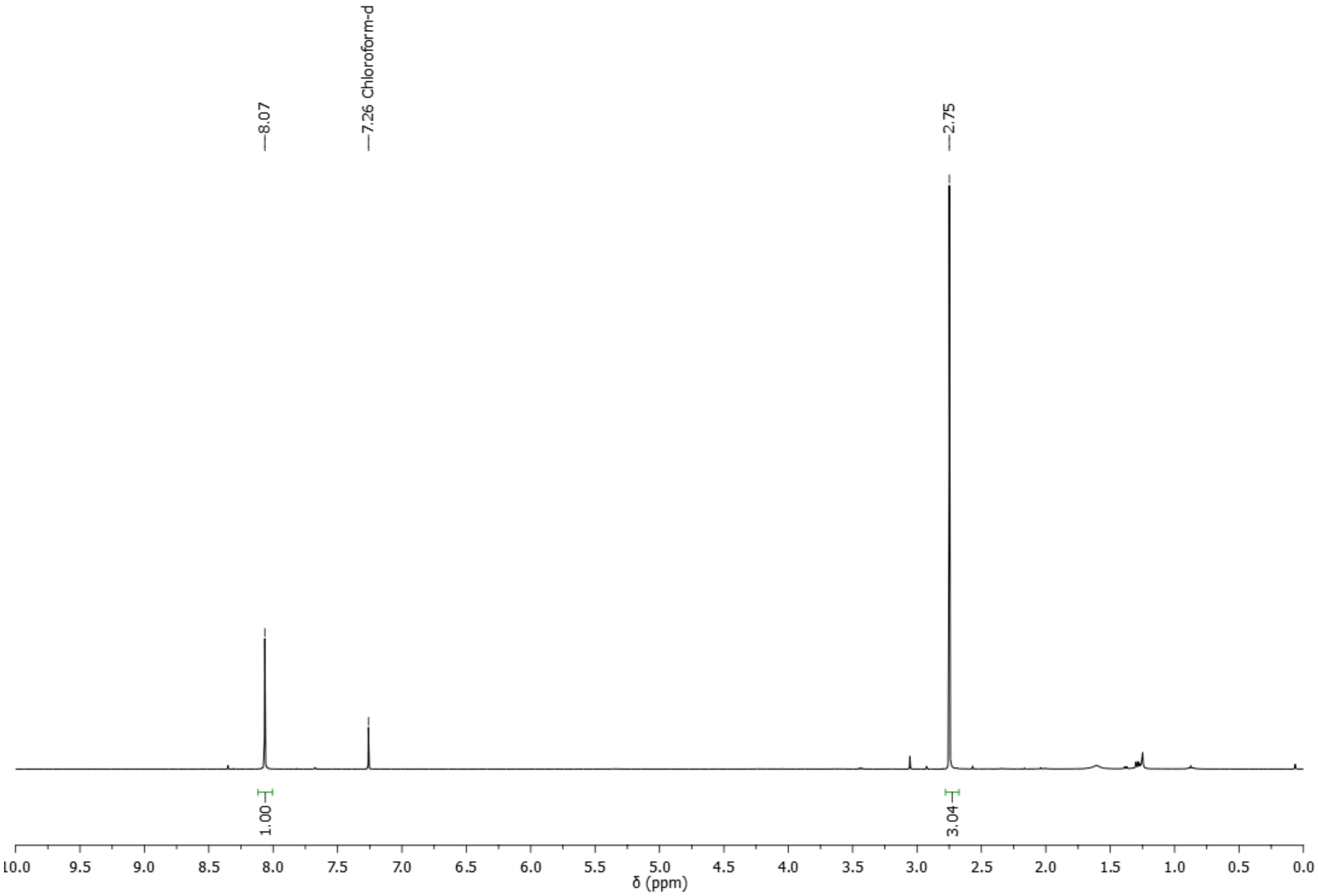

^{13}CNMR 100MHz, $CDCl_3$

3z

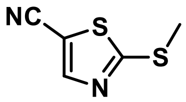

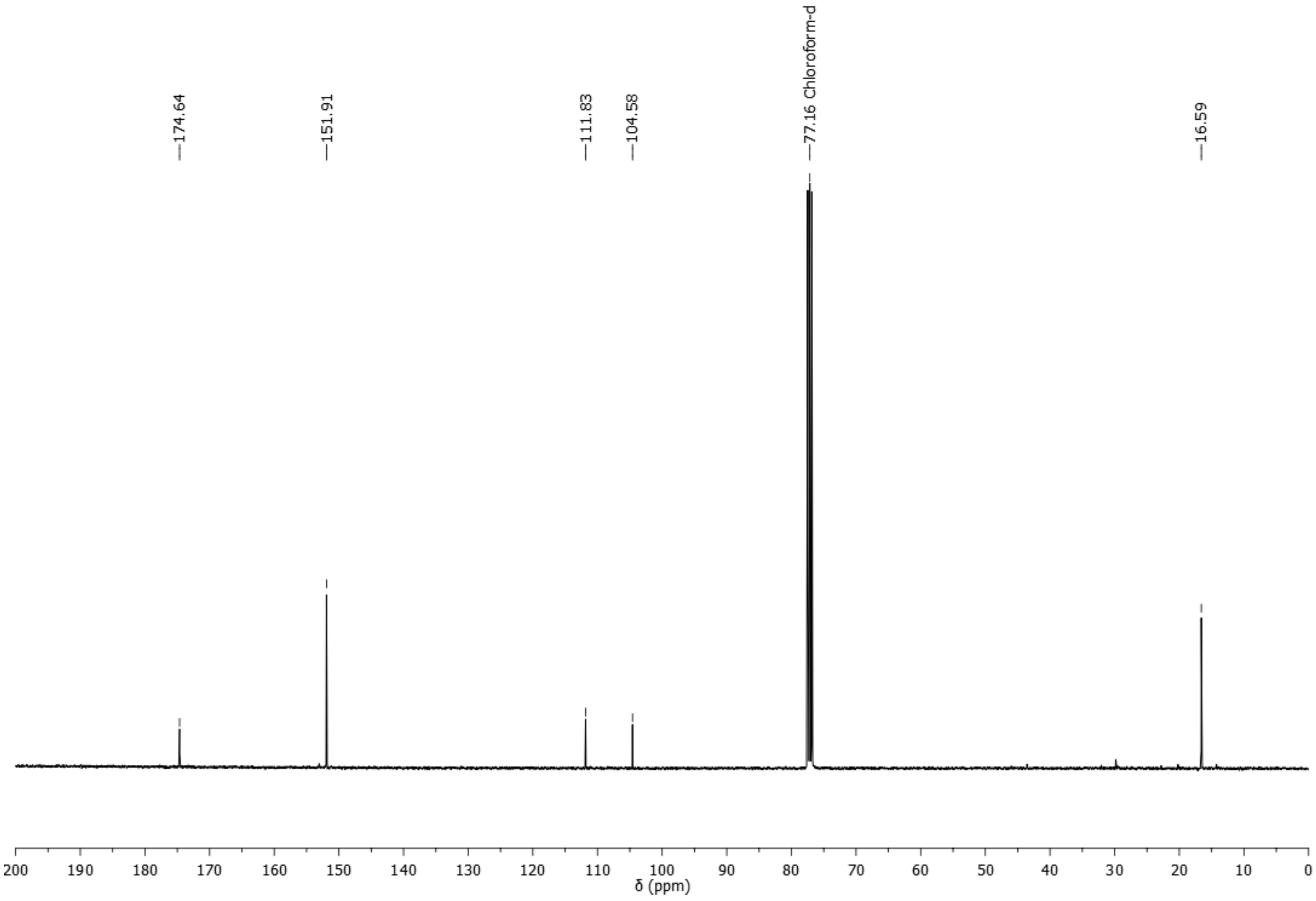

^{1}HNMR 400MHz, $CDCl_3$

3-Riv

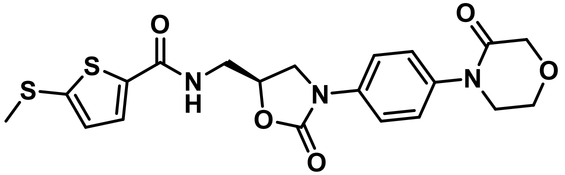

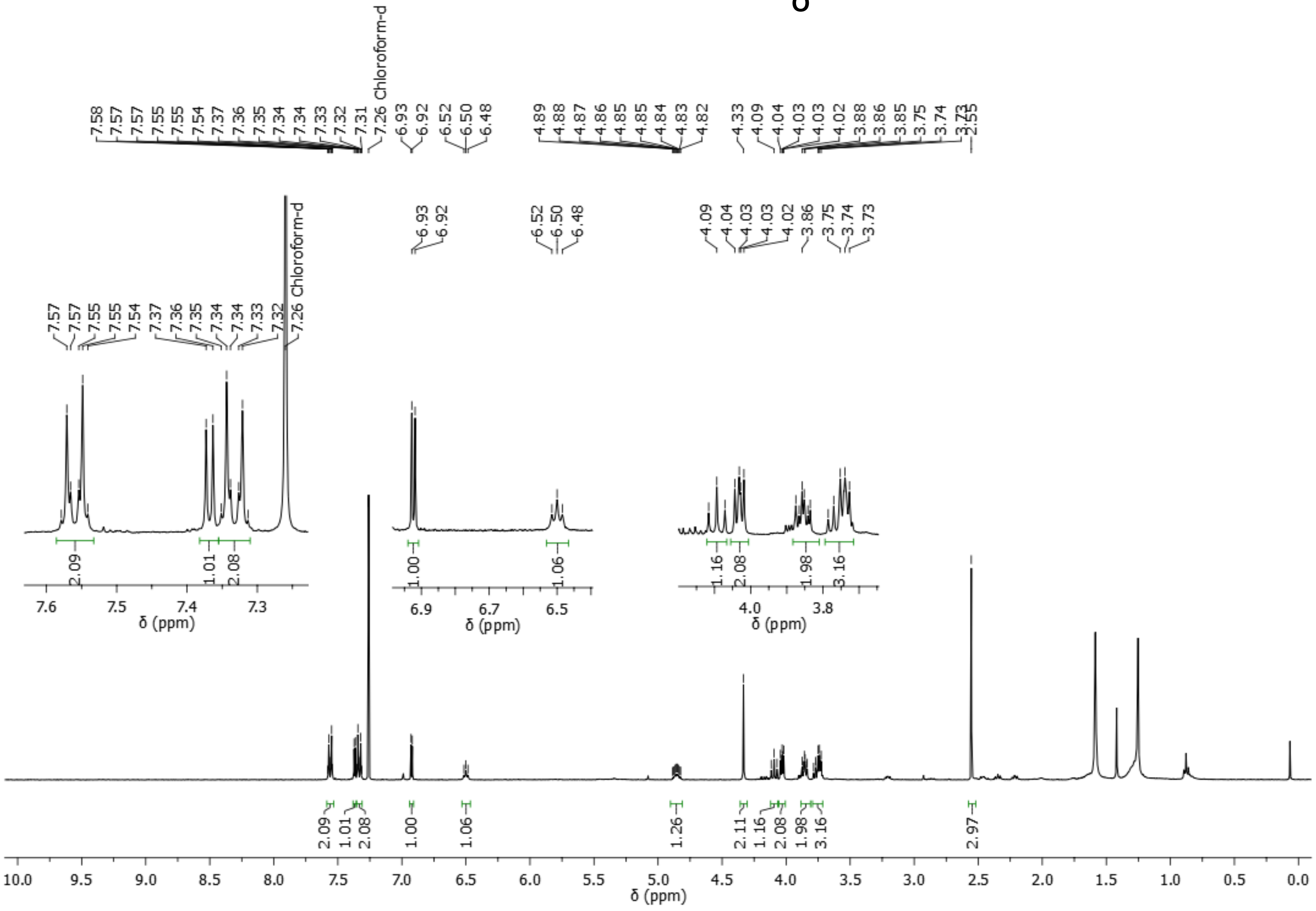

^{13}CNMR 100MHz, $CDCl_3$

3-Riv

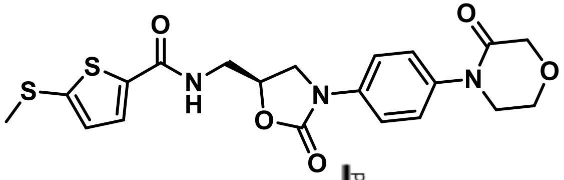

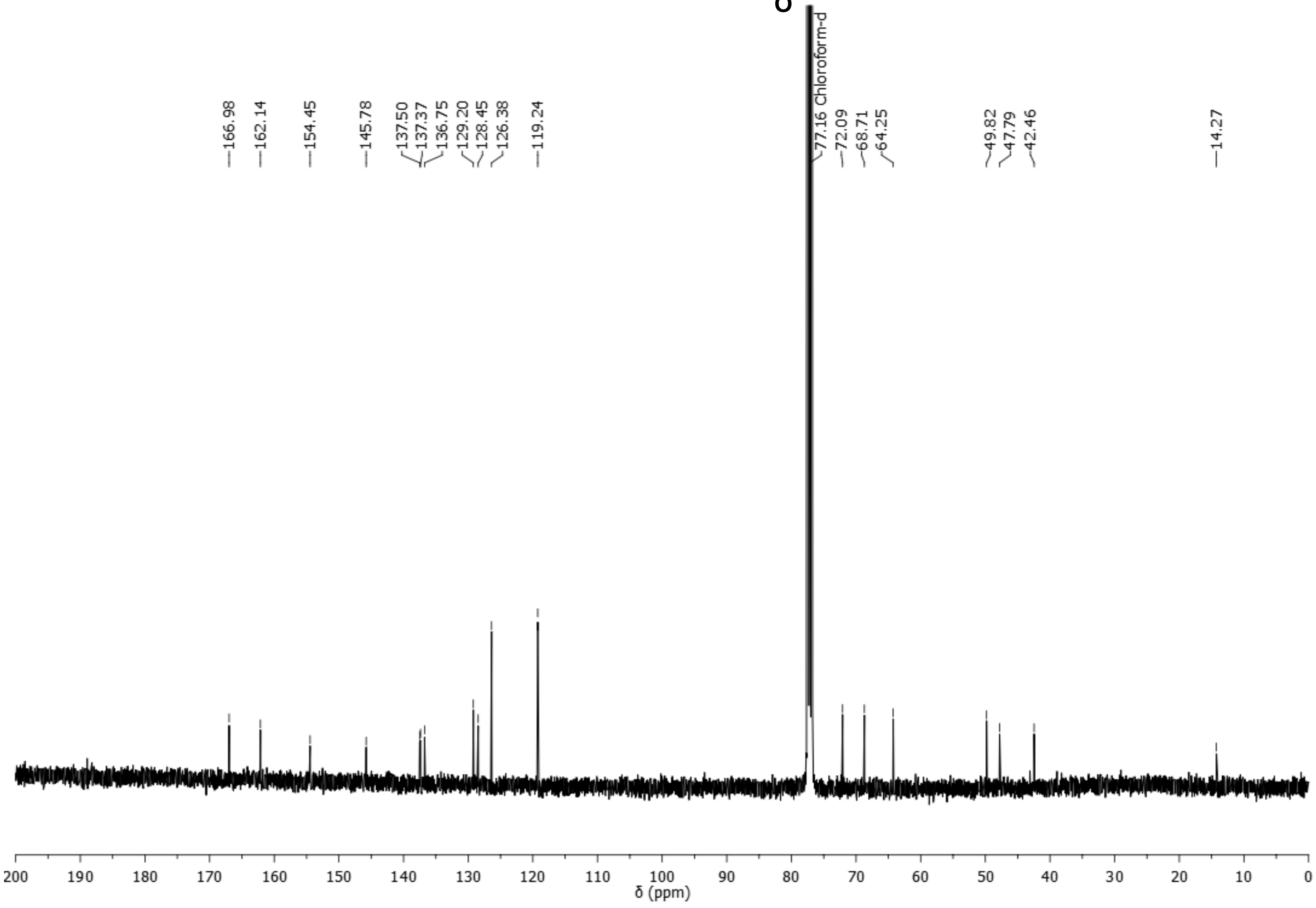

^{1}HNMR 400MHz, $CDCl_3$

4a

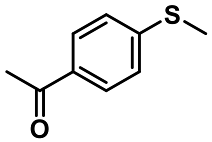

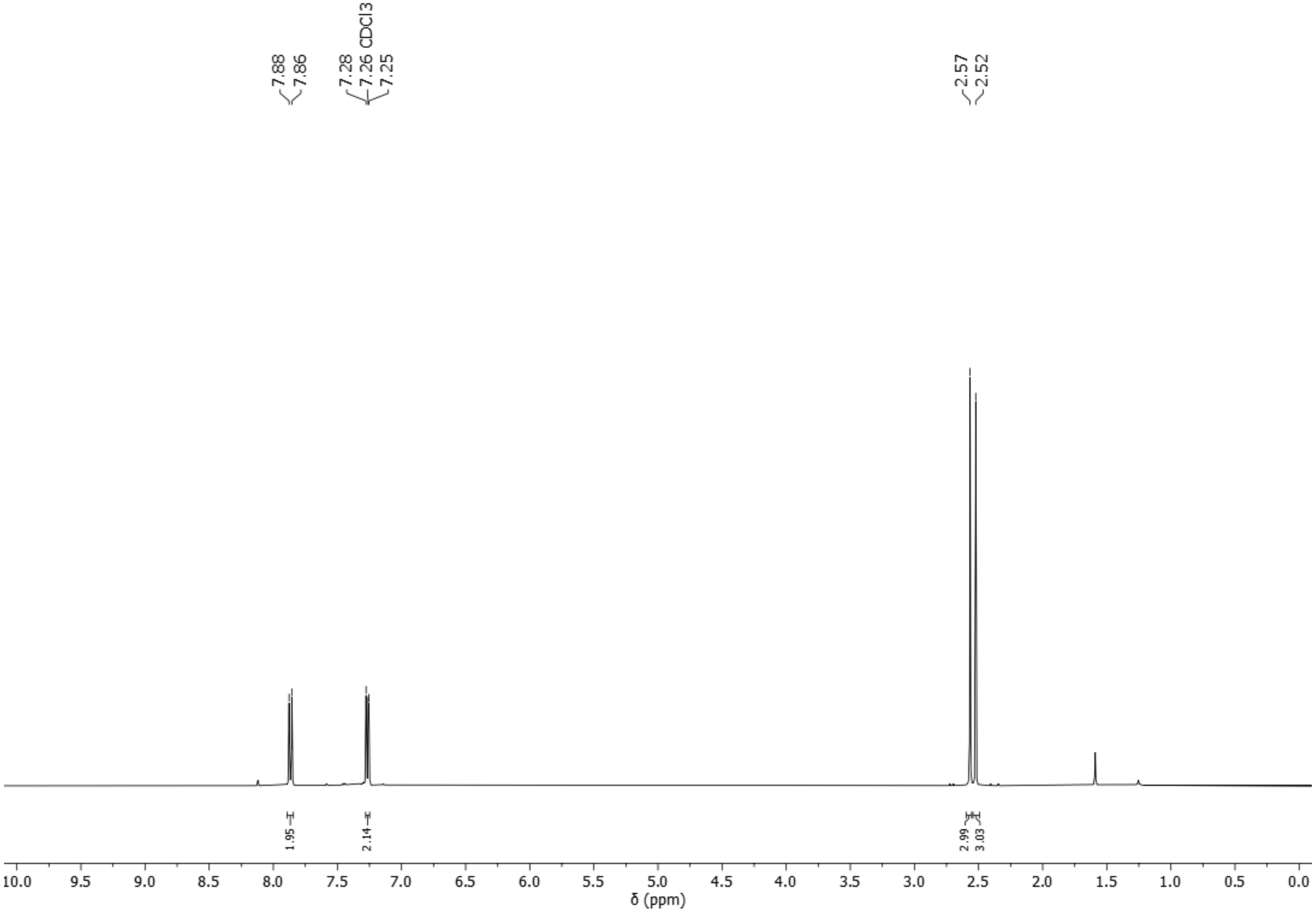

^{13}CNMR 100MHz, $CDCl_3$

4a

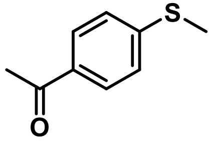

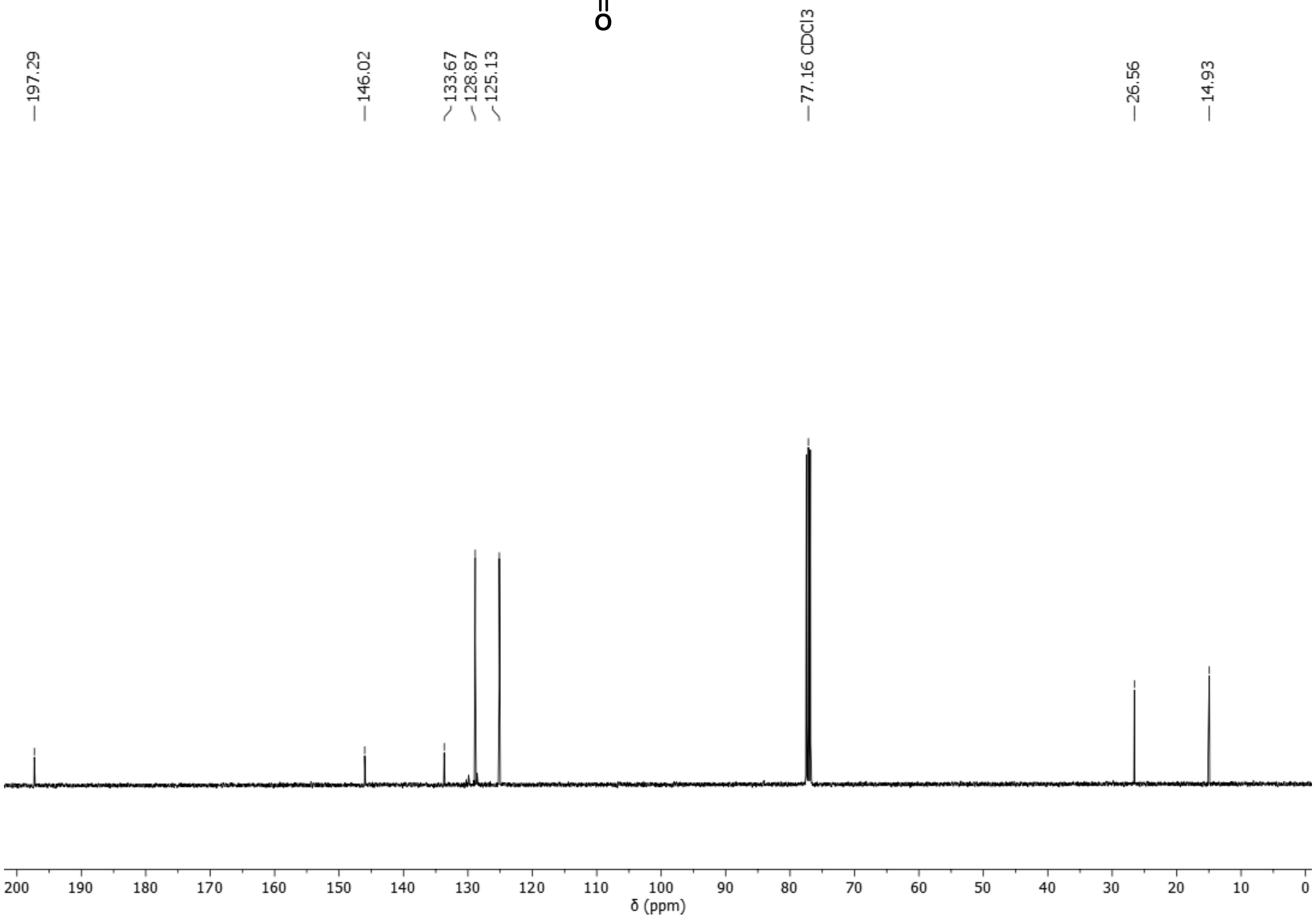

^{1}HNMR 400MHz, CD_2Cl_2

4b

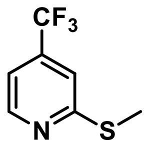

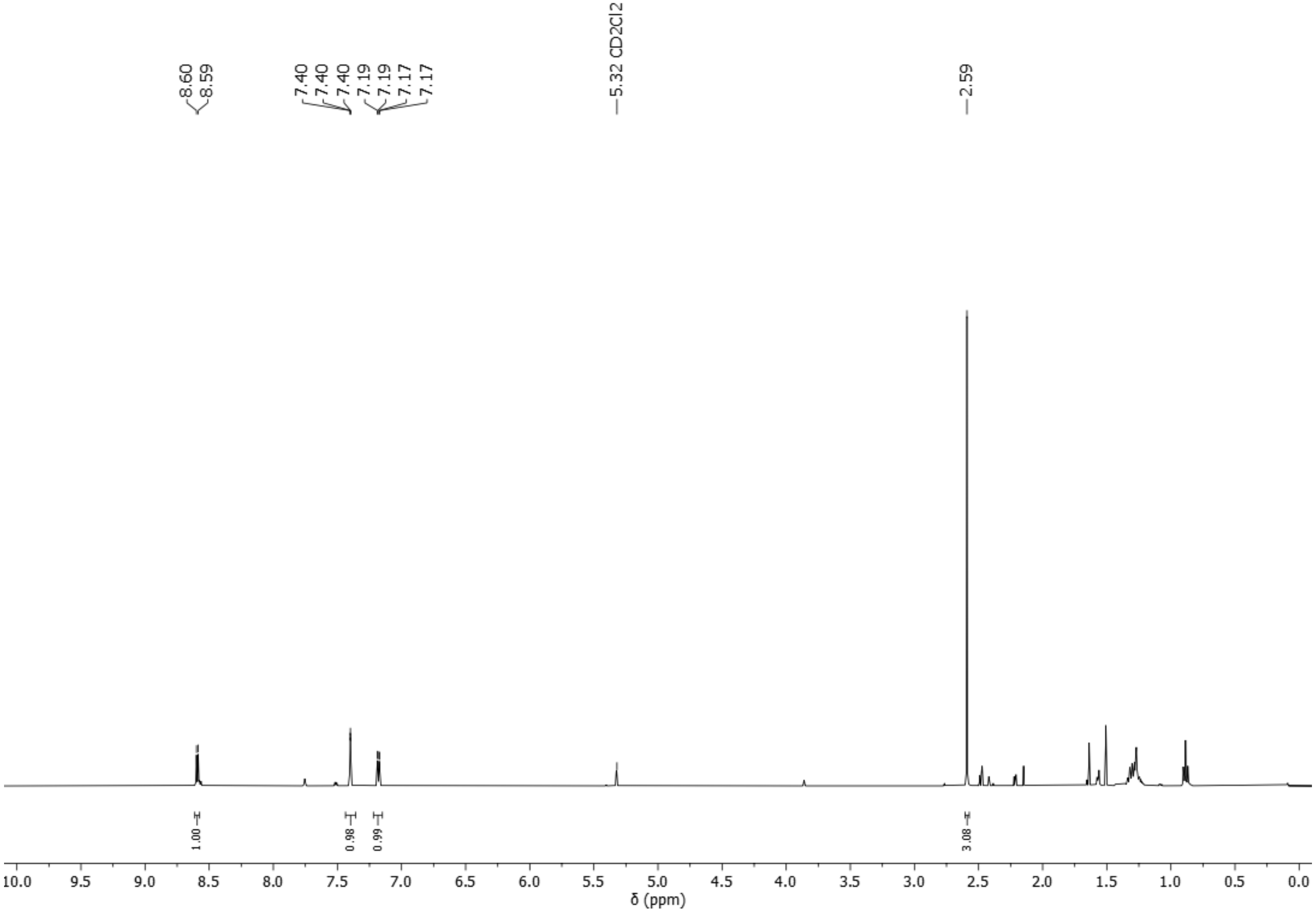

^{19}FNMR 377MHz, CD_2Cl_2

4b

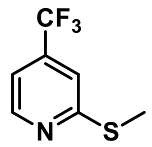

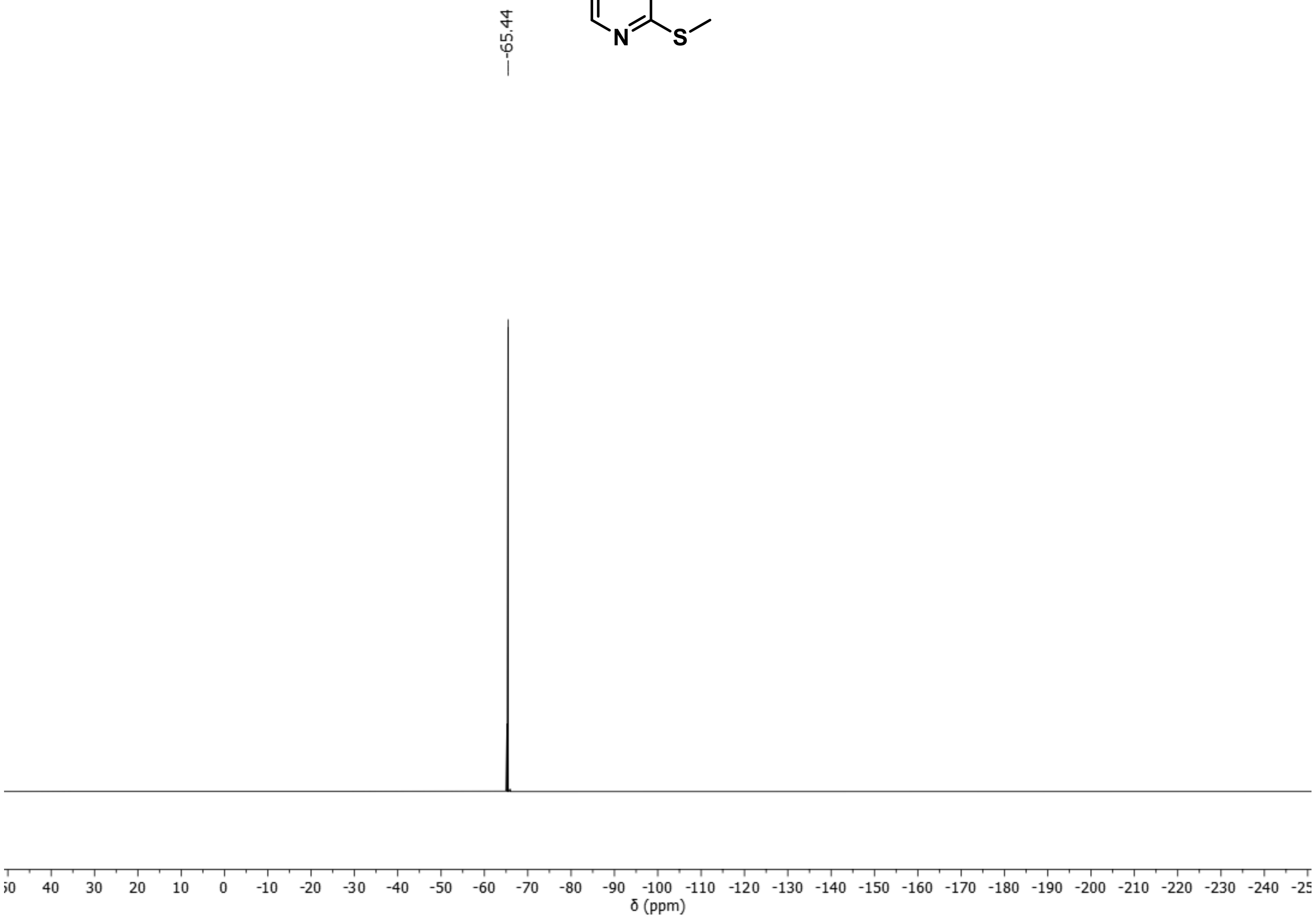

^{1}HNMR 400MHz, CDCl$_3$

4c

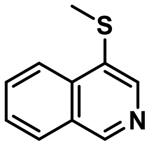

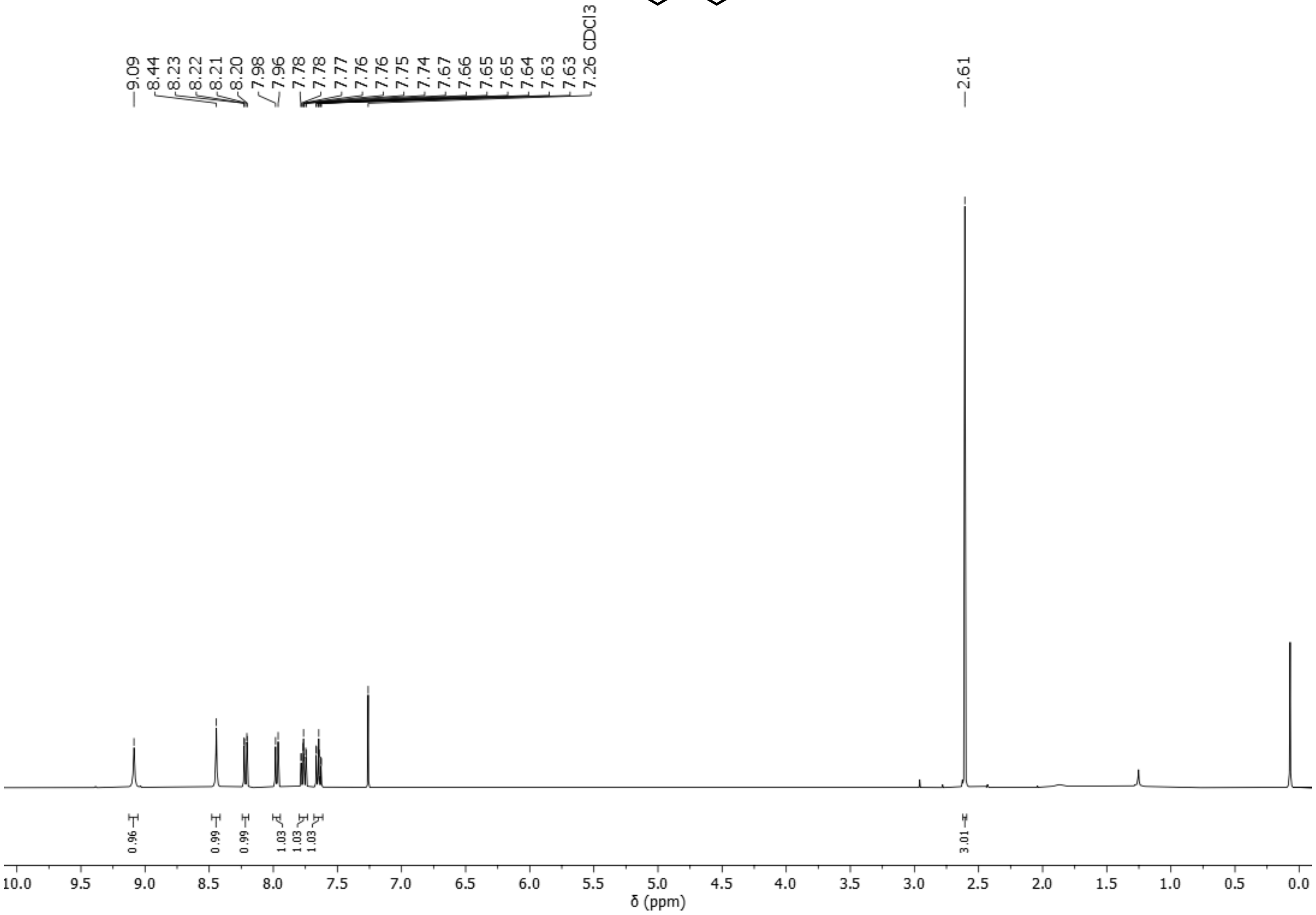

^{13}CNMR 100MHz, $CDCl_3$

4c

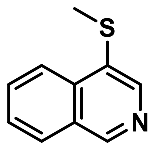

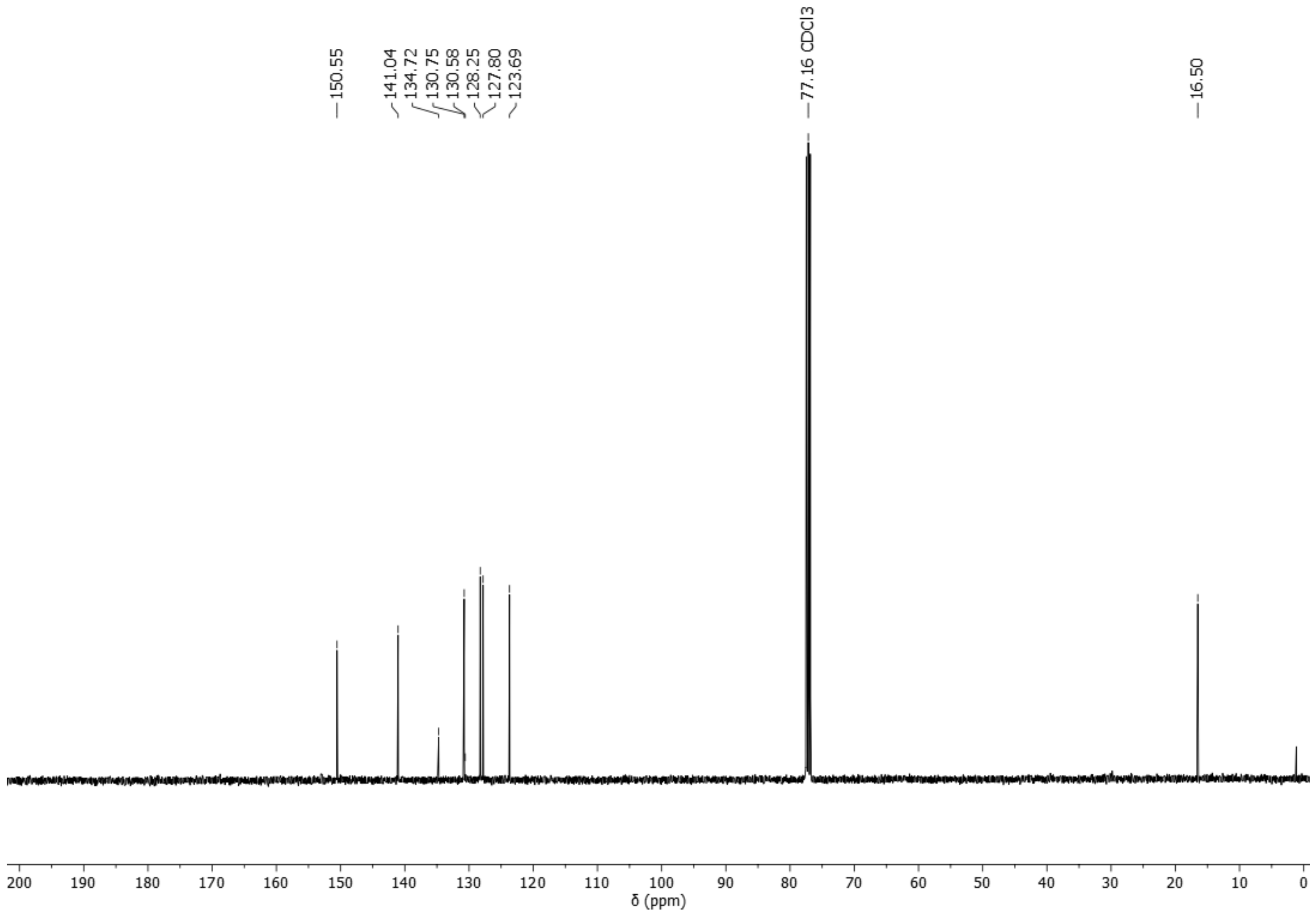

^{1}HNMR 400MHz, $CDCl_3$

4d

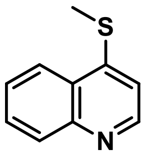

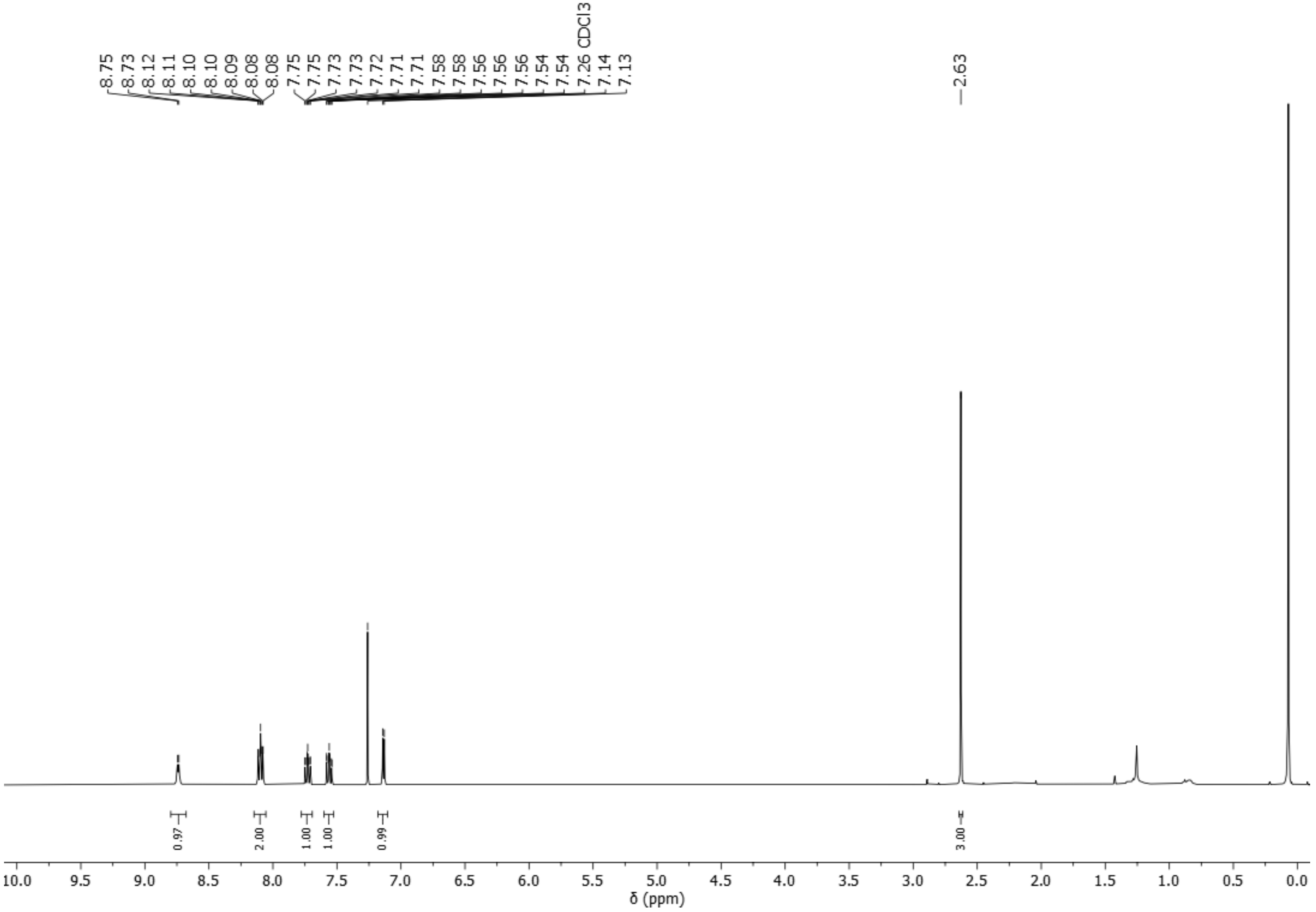

^{13}CNMR 100MHz, $CDCl_3$

4d

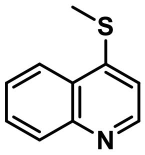

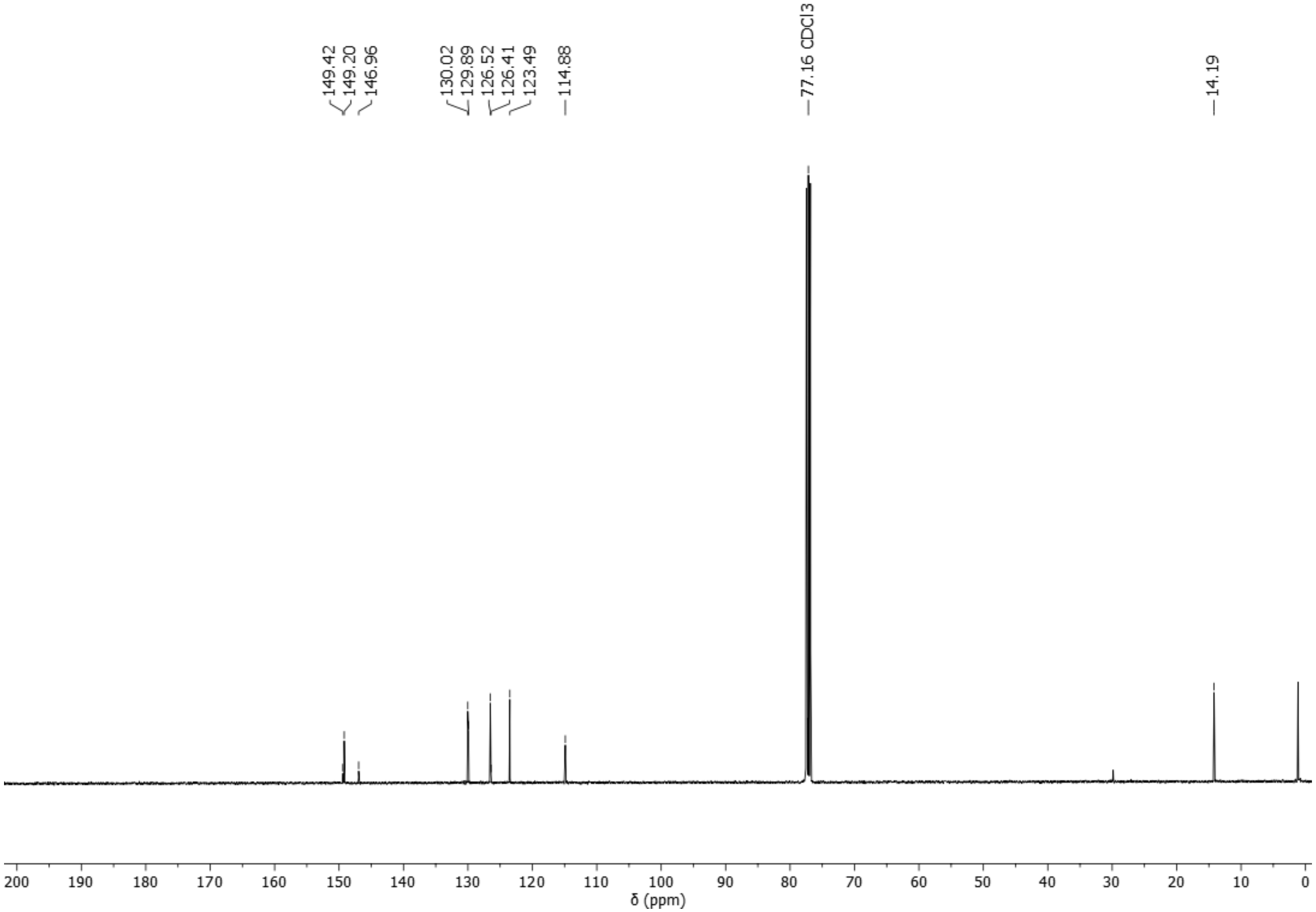

^{1}HNMR 400MHz, $CDCl_3$

4e

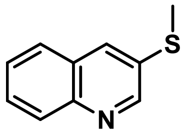

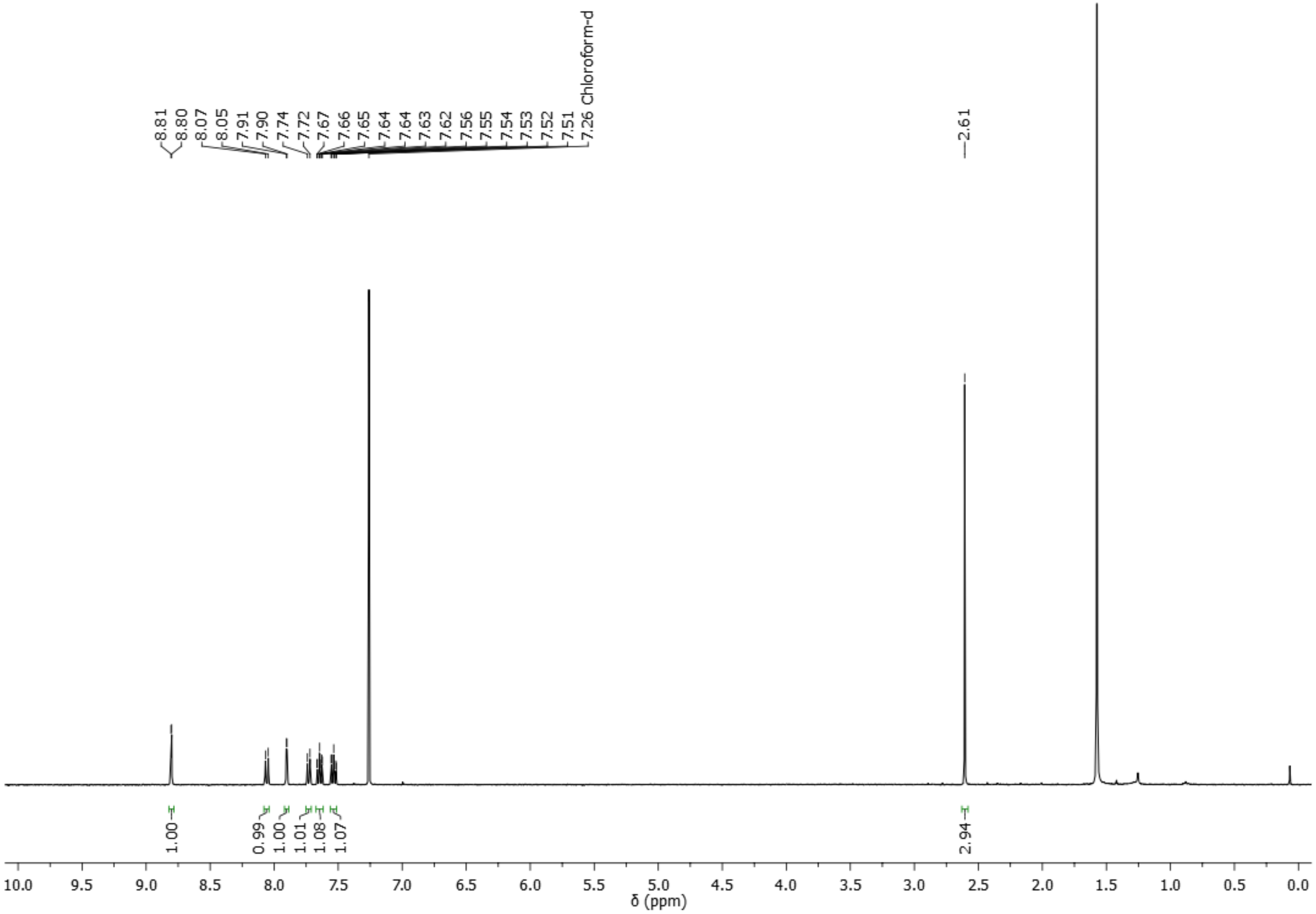

^{13}CNMR 100MHz, $CDCl_3$

4e

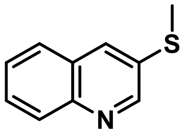

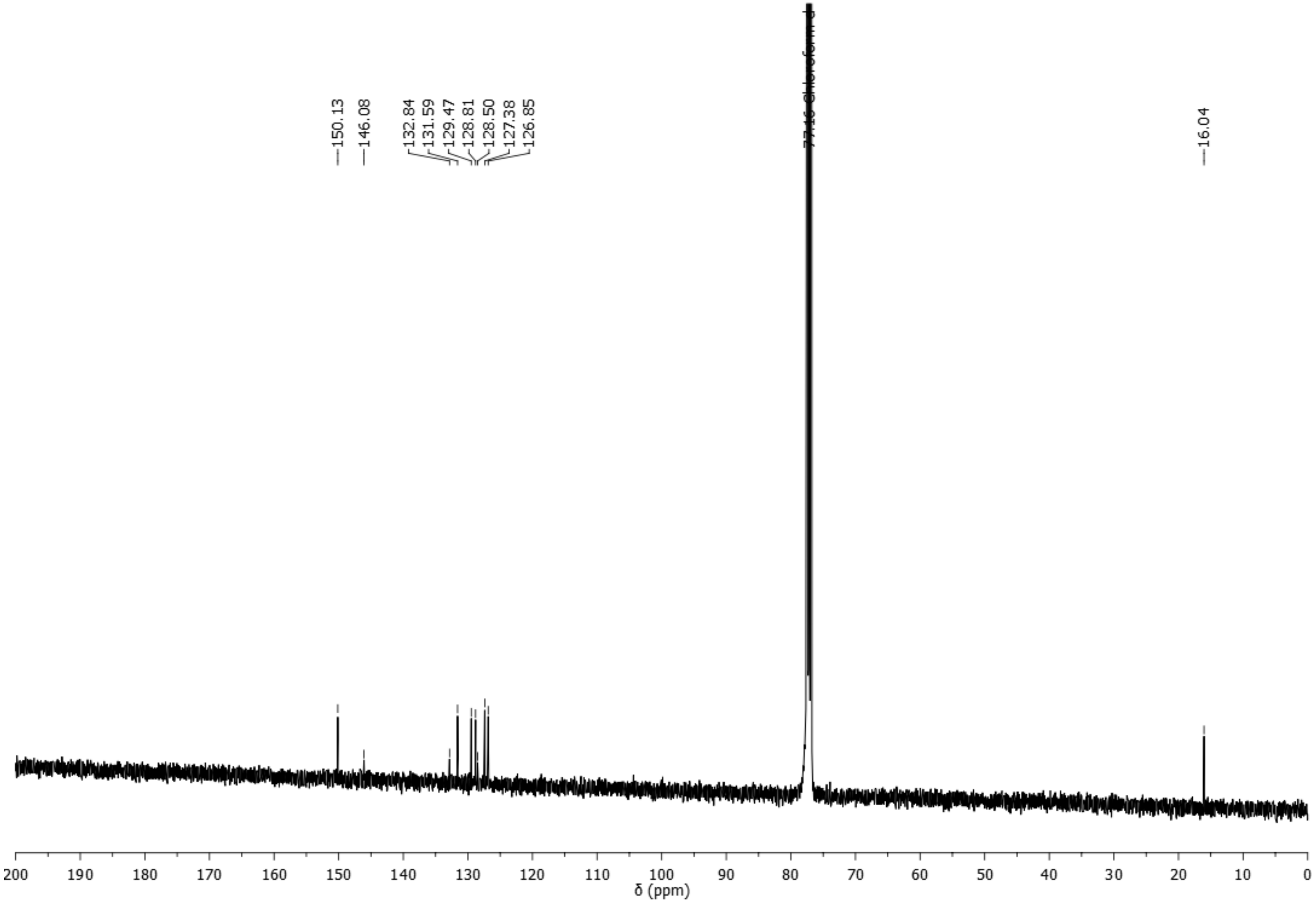

Supplement: Supplementary file 1 — Supporting Information [file CHEM-29-0-s001.pdf]
